# Terpyridine Diphosphine Ruthenium Complexes as Efficient Photocatalysts for the Transfer Hydrogenation of Carbonyl Compounds

**DOI:** 10.1002/chem.202201722

**Published:** 2022-09-27

**Authors:** Maurizio Ballico, Dario Alessi, Christian Jandl, Denise Lovison, Walter Baratta

**Affiliations:** ^1^ Dipartimento di Scienze AgroAlimentari Ambientali e Animali (DI4A) Università di Udine Via Cotonificio 108 33100 Udine Italy; ^2^ Department of Chemistry & Catalysis Research Center TUM Lichtenbergstraße 4 85747 Garching b. München Germany

**Keywords:** carbonyl compounds, photocatalysis, ruthenium, terpyridine, transfer hydrogenation

## Abstract

The cationic achiral and chiral terpyridine diphosphine ruthenium complexes [RuCl(PP)(tpy)]Cl (PP=dppp (**1**), (*R*,*R*)‐Skewphos (**2**) and (*S*,*S*)‐Skewphos (**3**)) are easily obtained in 85–88 % yield through a one‐pot synthesis from [RuCl_2_(PPh_3_)_3_], the diphosphine and 2,2′:6′,2′′‐terpyridine (tpy) in 1‐butanol. Treatment of **1**–**3** with NaPF_6_ in methanol at RT affords quantitatively the corresponding derivatives [RuCl(PP)(tpy)]PF_6_ (PP=dppp (**1 a**), (*R*,*R*)‐Skewphos (**2 a**) and (*S*,*S*)‐Skewphos (**3 a**)). Reaction of [RuCl_2_(PPh_3_)_3_] with (*S*,*R*)‐Josiphos or (*R*)‐BINAP in toluene, followed by treatment with tpy in 1‐butanol and finally with NaPF_6_ in MeOH gives [RuCl(PP)(tpy)]PF_6_ (PP=(*S*,*R*)‐Josiphos (**4 a**), (*R*)‐BINAP (**5 a**)) isolated in 78 % and 86 % yield, respectively. The chiral derivatives have been isolated as single stereoisomers and **3 a**, **4 a** have been characterized by single crystal X‐ray diffraction studies. The tpy complexes with NaO*i*Pr display high photocatalytic activity in the transfer hydrogenation (TH) of carbonyl compounds using 2‐propanol as the only hydrogen donor and visible light at 30 °C, at remarkably high S/C (up to 5000) and TOF values up to 264 h^−1^. The chiral enantiomers **2**, **2 a** and **3**, **3 a** induce the asymmetric photocatalytic TH of acetophenone, affording (*S*)‐ and (*R*)‐1‐phenylethanol with 51 and 52 % *ee*, respectively, in a MeOH/2‐propanol mixture.

## Graphical abstract

**The new cationic ruthenium [RuCl(PP)(tpy)]X (X=Cl, PF_6_) complexes**, prepared from [RuCl_2_(PPh_3_)_3_] a diphosphine and 2,2′:6′,2′′‐terpyridine (tpy), promote the photocatalytical transfer hydrogenation (TH) of carbonyl compounds in 2‐propanol under mild conditions. Unprecedented asymmetric catalytic TH of acetophenone driven by light was obtained with ruthenium tpy complexes containing the chiral Skewphos diphosphine.

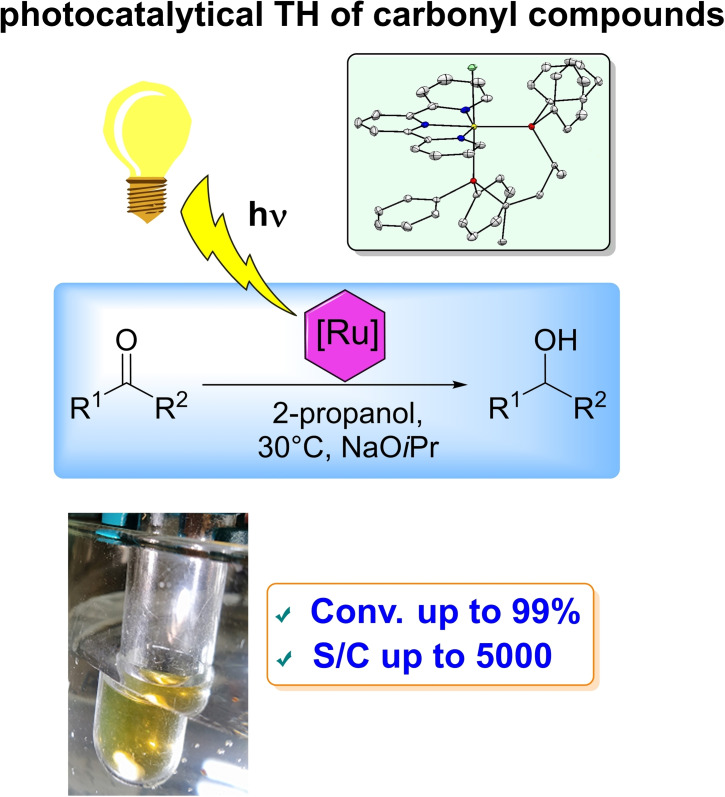

## Introduction

In the recent decades, visible‐light photocatalysis has become a valuable powerful tool for the synthesis of organic compounds via active radicals and radical ions, providing access to new molecular transformations, which cannot be achieved under thermal conditions.^[1]^ Transition‐metal complexes have been successfully employed in photocatalytic organic synthesis and particular attention has been devoted to ruthenium and iridium photosensitizers.^[2]^ Thus, [Ru(bpy)_3_]X_2_ and [Ir(ppy)_3−n_(bpy)_n_]X_n_ (n=0, 1; ppy=2‐phenylpyridine; bpy=2,2’‐bipyridine) type complexes have been used in a number of organic transformations, on account their favorable physical properties (i.e. long excited‐state lifetime, high luminescent efficiency).^[3]^ Mono and polynuclear ruthenium complexes based on polypyridine ligands have been extensively investigated in electron‐ and energy‐transfer processes.^[4]^ In this context, the non‐innocent redox‐active terpyridine (tpy) ligand has been used to obtain robust pincer complexes for catalysis and supramolecular chemistry.^[5]^ Although the [Ru(tpy)_2_]X_2_ species show reduced photophysical properties compared to the related bpy derivatives, on account of the rigidity and narrow bite angle of the NNN ligand,^[6]^ monodentate phosphines tpy complexes show attractive properties as near‐infrared sensitizers.^[7]^ In addition to the use of a photosensitizer as single species or in combination with an additional catalyst (dual photoredox catalysis), a transition metal complex may also play a double role by harvesting photon energy and catalyzing bond breaking/forming reactions via a traditional or a new type of mechanism, avoiding the employment of an exogenous photosensitizer (visible light‐induced transition metal catalysis).^[8]^ Despite the extensive use of [Ru(bpy)_3_]^2+^ in photoredox C−C coupling reactions,^[9]^ only a few examples of visible light‐induced ruthenium catalysts have been recently reported by Ackermann and Greaney, affording the functionalization of heteroarenes.^[10]^

Ruthenium complexes have been efficiently employed in C−C and C−H forming reactions via thermal homogenous catalysis. The transfer hydrogenation (TH)^[11]^ of carbonyl compounds is a widely accepted method in industry for the production of alcohols, using 2‐propanol or formic acid as reducing agents.^[12]^ This highly selective and atom economy process, compared to the classical one with NaBH_4_ or LiAlH_4_, makes TH a sustainable transformation in organic synthesis. Highly efficient catalysts are the arene amino [RuCl(arene)(NN)]^[13]^ developed by Noyori, the ampy‐type *cis*‐[RuCl_2_(ampy)(PP)]^[14]^ (ampy=2‐aminomethylpyridine), including the pincer [RuCl(CNN)(PP)],^[15]^ [RuCl(CNN)(PPh_3_)(CO)]^[16]^ complexes which also display catalytic activity in related C−H activation reactions.^[17]^ On the other hand, the pincer tpy complexes [RuCl_n_(tpy)(PPh_3_)_3−n_]X_2−n_ ^[18]^ (n=1, 2) show moderate catalytic activity in TH, while the diphosphine derivatives [RuY(tpy)(PP)]X and [Ru(L)(tpy)(PP)]X_2_ (PP=dppbz, dmpe)^[19]^ have not been investigated in catalysis.

It is worth noting that TH mediated by light has been reported using heterogeneous TiO_2_‐based, semiconductors, nanoparticles and metal oxides systems,^[20]^ whereas only few examples of homogeneous photocatalytic TH have been described. Thus, [RuCl_2_(tpy)(biq)] (biq=2,2’‐bisquinoline) is found to promote the visible‐light‐driven TH of NAD(P)^+^ to NAD(P)H using HCO_2_Na in water.^[21]^ The [Ru(bpy)_3_]^2+^/viologen couple catalyzes the reduction of 2‐phenyl‐2‐oxoethanoic acid using triethanolamine (TEOA) as a sacrificial donor,^[22]^ while [Ru(bpy)_3_]^2+^ photocatalytically affords hydrogen from TEOA.^[23]^ A light‐induced oxygen evolution from water at a single metal center was described by Milstein for the pincer [RuH(OH)(PNN)(CO)].^[24]^ As regards the group 9 metal complexes, [Cp*IrCl(bpy)]Cl^[25]^ was found to photocatalyze the TH of cyclohexanone with HCO_2_Na in the absence of any additional photosensitizer, while [Cp*RhCl(bpy)]Cl^[26]^ was proven to reduce aldehydes with proflavine as photocatalyst mediator with TEOA. Interestingly, chiral complexes and organocatalysts have demonstrated to induce asymmetric photochemical transformations,^[27]^ the helical chiral [Ru(bpy)_3_]X_2_ displaying a stereoinduced electron‐transfer process.^[28]^

We report herein a straightforward preparation of cationic tpy ruthenium complexes of general formula [RuCl(PP)(tpy)]X (X=Cl, PF_6_) from [RuCl_2_(PPh_3_)_3_], tpy and (chiral) diphosphines. These derivatives are efficient visible light‐induced ruthenium catalysts for the TH of carbonyl compounds using 2‐propanol. Asymmetric TH of acetophenone has been observed with the chiral tpy complexes. Ruthenium alkoxide and hydride species are also formed during catalysis under light irradiation.

## Results and Discussion

### Synthesis of terpyridine and diphosphine ruthenium complexes

**Scheme 1:**
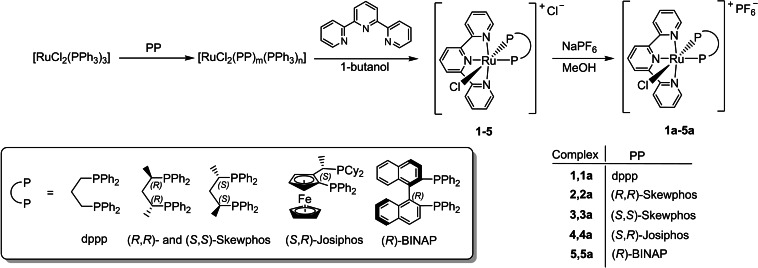
Synthesis of [RuCl(PP)(tpy)]X (X=Cl, PF_6_; PP=diphosphine) complexes.

Reaction of [RuCl_2_(PPh_3_)_3_] with dppp (1 equiv.) in 1‐butanol at 90 °C for 4 h, followed by treatment with tpy at reflux for 12 h affords the cationic ruthenium derivative [RuCl(dppp)(tpy)]Cl (**1**), isolated in 87 % yield, via a [RuCl_2_(dppp)_m_(PPh_3_)_n_] intermediate (Scheme 1).^[29]^ The ^31^P{^1^H} NMR spectrum of **1** in CD_2_Cl_2_ exhibits two doublets at *δ*=34.2 and 20.0 ppm with a ^2^ *J*(P,P) of 39.0 Hz for the P atoms *trans* to Cl and N atoms, respectively. The H6/H6” protons of the terminal pyridines give a doublet at *δ*=7.80 ppm (^3^ *J*(H,H)=6.1 Hz), upfield shifted compared to the free ligand (*δ*=8.69 ppm),^[30]^ possibly due to the anisotropic effect of the phenyls of dppp (for atom‐numbering see Figure 5). The ^13^C{^1^H} NMR resonances of the C6/C6” and C5/C5” carbons are at *δ*=156.7 and 126.3 ppm, downfield shifted with respect to the free tpy (Δ*δ*=7.2 and 5.2 ppm, respectively), whereas the carbon atoms of the central pyridine are less deshielded (Δ*δ*=0.8 ppm for C4’, Figure S4‐S6). Treatment of **1** with NaPF_6_ in methanol (30 min) affords [RuCl(dppp)(tpy)]PF_6_ (**1 a**) as a red precipitate in 93 % yield, by anion exchange (Scheme 1).

**Figure 1:**
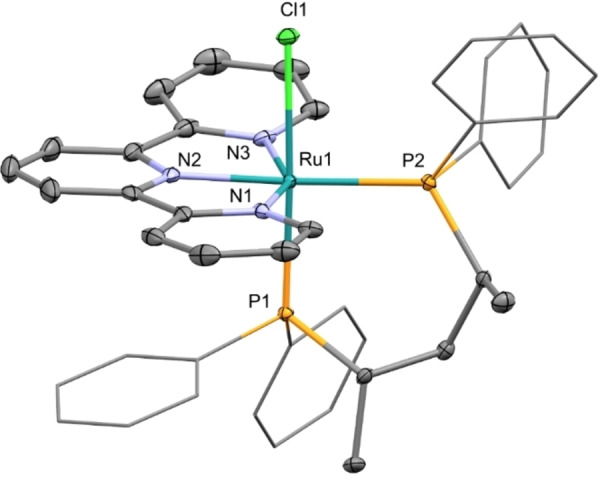
ORTEP style plot of compound **3 a** in the solid state (CCDC 2165467). Ellipsoids are drawn at the 50 % probability level. Hydrogen atoms, co‐crystallized solvent molecules, and the PF_6_ counterion are omitted, and phenyl groups are simplified as wireframes for clarity.

Replacement of the counterion does not cause any significant changes in the ^31^P{^1^H} NMR resonances, whereas the terpyridine H3’/H5’ and H3/H3” proton signals of **1 a** overlap with the phenyl ones. Similarly to **1**, treatment of [RuCl_2_(PPh_3_)_3_] with an equimolar amount of (*R*,*R*)‐Skewphos in 1‐butanol at 90 °C for 4 h, and subsequent reaction with tpy (1 equiv.) at reflux for 12 h, afforded the single stereoisomer [RuCl((*R*,*R*)‐Skewphos)(tpy)]Cl (**2**) as a red product, isolated in 88 % yield (Scheme 1). Complex **2** displays in the ^31^P{^1^H} NMR spectrum two doublets at *δ*=49.3 and 33.9 ppm (^2^ *J*(P,P)=38.5 Hz) for the P atoms *trans* to Cl and N atoms, respectively, as established through the long range ^*4*^ *J*(H,P) coupling between the terminal *ortho* H6 and H6” of tpy (at *δ*_H_=8.83 and 6.63 ppm) and the P *trans* to N (^31^P‐^1^H HMBC 2D NMR experiment, Figure S14). While the H6 resonance is slightly deshielded with respect to the free tpy, the H6” signal is strongly upfield (Δ*δ*=2.06 ppm), on account of the interaction with a phenyl of the Skewphos, showing a NOE with the *ortho* phenyl protons at *δ*=6.29 ppm (Figure S15). In addition, the CHCH_3_ moiety linked to the P *trans* to the chloride gives two signals at *δ*=2.55 and 0.63 ppm for CH and CH_3_ groups, while the resonances at *δ*=3.78 and 1.44 ppm are for the other CHCH_3_ unity, respectively. As for **1 a**, reaction of **2** with an excess of NaPF_6_ (2.5 equiv.) in methanol at RT led to the complex [RuCl((*R*,*R*)‐Skewphos)(tpy)]PF_6_ (**2 a**) in quantitative yield. By using (*S,S*)‐Skewphos, in place of (*R,R*)‐Skewphos and following the same procedures for the synthesis of **2** and **2 a**, the corresponding enantiomers **3** and **3 a** have been isolated in 85 and 95 % yield. The molecular structure of **3 a** has been confirmed by a single crystal X‐ray diffraction experiment, which allows to identify the stereochemistry at the ruthenium center (Figure 1).

The diphosphine complex **3 a** exhibits a pseudo‐octahedral geometry with the chloride orthogonal to the tpy ligand, which has one pyridyl group slightly bent upwards. The Ru1‐N2 distance for the internal nitrogen of tpy is 2.020(2) Å, which is shorter with respect to the terminal Ru1‐N1 and Ru1‐N2 lengths (2.107(2) and 2.148(2) Å), as observed in [Ru(MeCN)(tpy)(dppbz)](PF_6_)_2_,^[19b]^ while the N1‐Ru1‐N3 angle is 154.97(10)°, in line with that for other tpy ruthenium complexes.[^18a^, ^30^, ^31^] The Ru1‐P2 distance (2.3745(8) Å) is significantly longer than the Ru1‐P1 (2.2893(8) Å) one, but both are in the typical range of phosphine ruthenium complexes (2.26–2.41 Å).^[32]^ The crystal structure analysis shows the presence of additional intramolecular π‐π interactions between the phenyl groups of the Skewphos and the pyridine rings of the tpy ligand, also in line with the behavior of **3 a** in solution with one phenyl displaying high‐field shifted ^1^H NMR signals.

**Figure 2:**
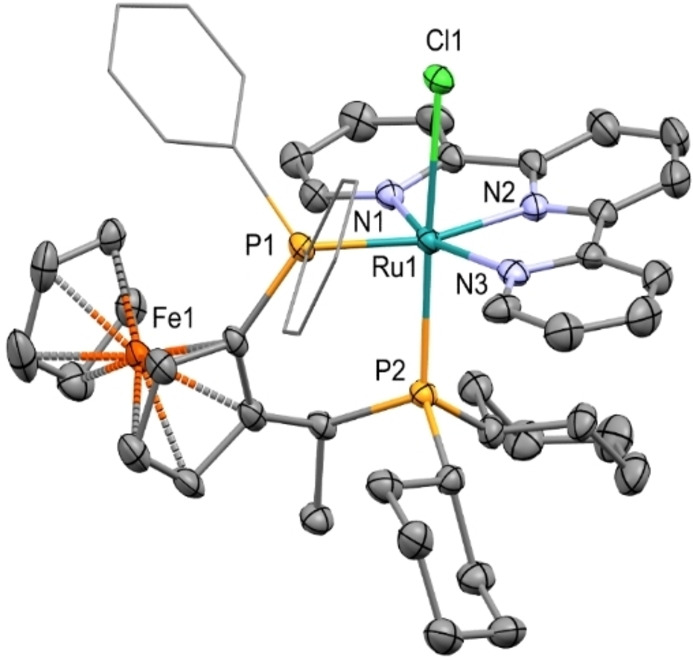
ORTEP style plot of compound **4 a** in the solid state (CCDC 2165468). Ellipsoids are drawn at the 50 % probability level. Hydrogen atoms, co‐crystallized solvent molecules, and the PF_6_ counterion are omitted, and phenyl groups are simplified as wireframes for clarity.

Finally, the cationic derivatives [RuCl((*S*,*R*)‐Josiphos)(tpy)]PF_6_ (**4 a**) and [RuCl((*R*)‐BINAP)(tpy)]PF_6_ (**5 a**) have been obtained through a one‐pot reaction from [RuCl_2_(PPh_3_)_3_] and the corresponding chiral diphosphine, followed by treatment with tpy in 1‐butanol at reflux, giving the chloride complexes **4** and **5** which have been characterized in solution by NMR spectroscopy (see experimental part). Addition of NaPF_6_ allow the precipitation of **4 a** and **5 a** in pure form as single stereoisomers in 78 and 86 % yield, respectively, (Scheme 1). The ^31^P{^1^H} NMR spectrum of **4 a** and **5 a** give two doublets at *δ*=47.6 and 29.0 ppm (^2^ *J*(P,P)=35.0 Hz) and two very close doublets at *δ*=40.6 and 39.5 ppm (^2^ *J*(P,P)=30.3 Hz), respectively. For **4 a**, the terminal H6 pyridine proton is downfield shifted at *δ*_H_=9.42 ppm, whereas the corresponding H6” is upfield at *δ*_H_=6.41 ppm due the anisotropic shielding of a diphosphine cyclohexyl rings showing a methylene proton upfield shifted at *δ*_H_=−0.10 ppm. The interaction of a cyclohexyl ring with the tpy has been evidenced also by the crystal structure of **4 a**, showing that the tpy plane is slightly tilted out of the equatorial plane due to the steric hindrance of a Cy group (Figure 2).

As a matter of fact the N2−Ru1−P2 and N2−Ru1−Cl1 angles in **4 a** are 97.83(9)° and 81.11(9)°, respectively, with a higher distortion with respect to **3 a** (91.14(7)° and 86.44(7)°) and with a Ru1−N2 distance of 2.009(3) Å. Conversely, the *mer‐*RuCl(PP) arrangement has the expected geometrical features, with a Ru−P2 distance of 2.3287(10) Å similar to that of **3 a** and the related [RuCl_2_((*R*,*S*)‐Josiphos)(*S*)‐MePyme] derivative.^[14a]^ The H6/H6” pyridine protons of tpy of **5 a** are at *δ*=9.43 and 6.23 ppm, significantly shifted with respect to the free ligand (Δ*δ*=+0.74 and −2.46 ppm, respectively), while the corresponding C6/C6’ carbon atoms show Δ*δ*_C_ values up to 11.0 ppm compared to the free tpy, as inferred from COSY, NOESY and HSQC experiments.

### Photocatalytic TH of carbonyl compounds promoted by terpyridine ruthenium complexes

**Scheme 2:**
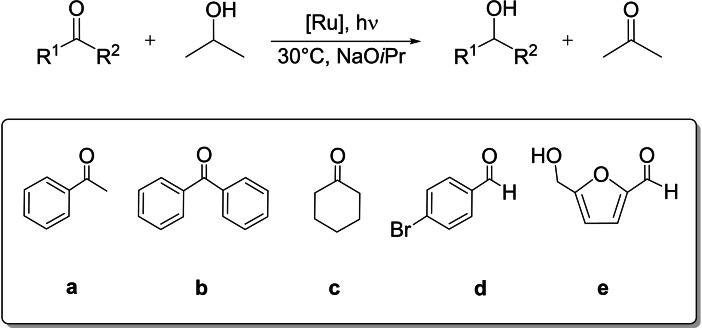
TH of carbonyl compounds photocatalyzed by terpyridine ruthenium complexes.

The catalytic activity of the pincer complexes **1**–**3** and **2 a**–**5 a** has been investigated in the TH of ketones and aldehydes to alcohols under light irradiation with a solar simulator (300 W Xenon Arc Lamp). Interestingly, the tpy ruthenium derivatives are found active at remarkably high S/C up to 5000 with 2‐propanol as the only hydrogen donor and without the use of sacrificial reductants (e.g. triethanolamine) or photosensitizers (Scheme 2). In addition, asymmetric photocatalytic TH of acetophenone **a** has been observed with the chiral pincer derivatives in 2‐propanol/MeOH mixtures.

Complex **1** (S/C=1000), bearing the dppp diphosphine, catalyzes the TH of **a** (0.1 M) in 2‐propanol with NaO*i*Pr (2 mol %) at 30 °C, affording 94 % conversion into 1‐phenylethanol in 16 h and with TOF of 74 h^−1^ (entry 1 of Table 1), while in the dark negligible conversion of **a** (1 %) is observed.

**Table 1:** Photocatalytic TH of **a** (0.1 M) with **1–3** and **2 a**‐**5 a** (S/C=1000) at 30 °C in the presence of NaO*i*Pr.

Entry	Complex	Solvent	NaO*i*Pr [mol %]	Time^[a]^ [h]	Conv.^[b]^ [%]	TOF^[c]^ [h^−1^]	*ee* ^[b]^ [%]
1	**1**	iPrOH	2.0	16	94	74	–
2	**2**	iPrOH	2.0	8	96	148	–
3	**2 a**	iPrOH	2.0	8	98	141	–
4	**2** ^[d]^	iPrOH	2.0	24	86	205	*–*
5	**2**	iPrOH	1.0	15	95	66	–
6	**2**	iPrOH	5.0	8	92	122	–
7	**2**	iPrOH	10.0	15	94	92	–
8	**2**	iPrOH/MeOH (1 : 1)	2.0	24	89	54	51 *S*
9	**2 a**	iPrOH/MeOH (1 : 1)	2.0	24	84	51	51 *S*
10	**3**	iPrOH	2.0	8	98	139	–
11	**3 a**	iPrOH	2.0	10	99	144	–
12	**3**	iPrOH/MeOH (1 : 1)	2.0	24	87	54	52 *R*
13	**3 a**	iPrOH/MeOH (1 : 1)	2.0	27	90	56	52 *R*
14	**4 a**	iPrOH	2.0	14	95	94	–
15	**4 a**	iPrOH/MeOH (1 : 1)	2.0	24	88	39	14 *S*
16	**5 a**	iPrOH	2.0	14	98	88	–
17	**5 a**	iPrOH/MeOH (1 : 1)	2.0	30	89	38	18 *S*
18	**2** ^[e]^	iPrOH	2.0	8	94	135	–
19	[Ru(bpy)_3_](PF_6_)_2_	iPrOH	2.0	8	61	73	–

[a] Irradiation hours. [b] The conversions and *e.e*. were determined by GC analysis. [c] Turnover frequency (moles of ketone converted to alcohol per mole of catalyst per hour) at 50 % conversion. [d] S/C=5000. [e] Hg poisoning test (Hg/**2**=400).

By using **2**, bearing (*R*,*R*)‐Skewphos, a complete reduction of **a** occurs in 8 h, with TOF of 148 h^−1^, affording the alcohol as racemic mixture (entry 2) and much of the same activity has been observed with **2 a**, indicating that the type of counterions (Cl^−^ vs. PF_6_^−^) does not affect the photocatalysis (entry 3). Interestingly, **2** catalyzes the TH of **a** at S/C=5000, with 86 % conversion in 24 h and high TOF=205 h^−1^ (entry 4). A decrease of rate has been observed for different amounts of NaO*i*Pr (1, 5 and 10 mol %) (entries 5–7) with respect to 2 mol %, whereas no photocatalysis occurs in the absence of the base.

Surprisingly, by using a 1/1 (v/v) mixture of 2‐propanol and MeOH, the complex **2** promotes the photocatalytic TH of acetophenone (S/C=1000), affording (*S*)‐1‐phenylethanol (89 % conv) with 51 % *ee* after 24 h of irradiation, but with a lower rate (TOF=54 h^−1^; entry 8) and a similar result has been obtained with **2 a** (entry 9). Using methanol as solvent, the same enantioselectivity has been achieved, but with only 6 % conversion in 10 h, due to the poor hydrogen donor capability of MeOH with respect to 2‐propanol.^[33]^ Conversely, **3** and **3 a** (S/C=1000) in 2‐propanol afford the TH of **a** in 10 h (TOF up to 144 h^−1^, entries 10 and 11), whereas in MeOH/*i*PrOH (1/1 in volume), 52 % *ee* of (*R*)‐1‐phenylethanol with 87 % and 90 % conv. is attained (TOF=54‐56 h^−1^, entries 12 and 13).

The *ee* values of the *S* and *R* alcohols are much the same, within the experimental error, and are consistent with the use of enantiomer catalysts. The derivatives **4 a** and **5 a**, bearing the diphosphine (*S*,*R*)‐Josiphos and (*R*)‐BINAP, respectively, lead to 95 and 98 % photocatalytic conversion of **a** in 14 h with TOF=94 and 88 h^−1^, respectively (entries 14 and 16), whereas using a MeOH/2‐propanol (1/1) mixture, leads to poor chiral induction (14 and 18 % *ee* of the *S* alcohol; entries 15 and 17). The comparison of the TH catalyzed by the pincer **4 a** in MeOH/2‐propanol (upon irradiation) with [RuCl(CNN)(*S*,*R*)‐Josiphos)]^[34]^ in 2‐propanol (under thermal conditions) containing the same diphosphine, affords the *S* alcohol as predominant enantiomer. Conversely, **3** and **3 a** afford the *R* enantiomer, while the corresponding [RuCl(CNN)(*S*,*S*)‐Skewphos)] derivatives^[35]^ give the *S* one. A mercury poisoning test^[36]^ carried out with **2** shows the same performance, suggesting that the catalysis occurs in homogeneous phase (entry 18). It is worth pointing out that no decrease of the enantioselectivity or deactivation of the ruthenium catalyst has been observed during irradiation. To the best of our knowledge, this is the first example of an asymmetric TH of a ketone promoted by light. A solvent effect on the catalytic asymmetric hydrogenation of C=O and C=N bonds has been previously reported,^[37]^ leading in same cases to a reversal of enantioselectivity.^[37b]^ Thus, the influence of methanol suggests that the asymmetric catalysis occurs within the chiral environment of the photocatalyst, possibly through π‐stacking interactions between the tpy rings and the phenyl ring of the substrate,^[38]^ favored by the hydrogen bonding of methanol vs. 2‐propanol media. Notably, the well‐known photosensitizer [Ru(bpy)_3_](PF_6_)_2_ has been shown to photocatalyze the TH of **a** leading to incomplete reduction (61 % conv.) in 8 h at 30 °C and with a TOF of 73 h^−1^ under the same catalytic conditions (entry 19).

To enlarge the scope of the photocatalytic TH, the NNN pincer complexes have been studied in the reduction of (bulky) ketones and aldehydes following the optimized protocol. Thus, **1** photocatalyzes the complete reduction of benzophenone **b** to benzhydrol at S/C=1000 after 12 h of irradiation, with a TOF=115 h^−1^ (entry 1, Table 2). With **2** at S/C=1000 and 5000, the reaction occurs faster with 99 % and 95 % conversions in 6 and 22 h and TOF up to 264 h^−1^, respectively (entries 2 and 3). Complex **1** catalyzes the TH of cyclohexanone **c** to cyclohexanol (91 % conv.) at S/C=1000 in 18 h (TOF=82 h^−1^, entry 4), while with **2** at S/C=1000 and 5000, **c** is quantitatively reduced in 8 and 25 h, respectively (TOF up to 207 h^−1^; entries 5, 6). The (*S*,*R*)‐Josiphos derivative **4 a** affords 99 % conversion of **c** in 21 h at S/C=500 (entries 7). The aldehyde 4‐bromo‐benzaldehyde **d** is reduced with **1** (S/C=1000) to the corresponding alcohol (88 % conv.) in 21 h, whereas with **2** quantitative reduction occurs within a shorter time frame (18 h) (entries 8, 9).

**Table 2:** Photocatalytic TH of ketones and aldehydes (0.1 M) in 2‐propanol with complexes **1**, **2** and **4 a** at 30 °C in the presence of 2 mol% NaO*i*Pr.

Entry	Complex	Substrate	S/C	Time^[a]^ [h]	Conv.^[b]^ [%]	TOF^[c]^ [h^−1^]
1	**1**	**b**	1000	12	98	115
2	**2**	**b**	1000	6	99	264
3	**2**	**b**	5000	22	95	256
4	**1**	**c**	1000	18	91	82
5	**2**	**c**	1000	8	98	138
6	**2**	**c**	5000	25	97	207
7	**4 a**	**c**	500	21	99	25
8	**1**	**d**	1000	21	88	46
9	**2**	**d**	1000	18	99	75
10	**2**	**d** ^[d]^	1000	18	88	49
11	**2**	**e**	1000	24	96	40

[a] Irradiation hours. [b] The conversions were determined by GC analysis. [c] Turnover frequency (moles of ketone converted to alcohol per mole of catalyst per hour) at 50 % conversion. [d] Reactions performed using 5 mol% K_2_CO_3_ as base.

Employment of K_2_CO_3_ (5 mol%) as base leads to a lower rate, with 86 % conversion in 18 h (entry 10). Finally, the biomass derivative 5‐(hydroxymethyl)furfural (5‐HMF) **e** is efficiently reduced to 2,5‐bis(hydroxymethyl)furan (BHMF) (96 % conv.) in 24 h with **2** at S/C=1000 (entry 11).

Attempts to reduce 2‐Me‐acetophenone, 2‐MeO‐acetophenone with **3 a** in iPrOH/MeOH (1 : 1) failed, while 4‐Me‐acetophenone affords 16 % of *R* alcohol (4 % *ee*) in 36 h (Table S1). A diastereoselective reduction was observed with Cyrene (dihydrolevoglucosenone) leading to levoglucosanol with an *erythro*/*threo* ratio of 1 : 1.7 (22 % conv.). Finally, *trans*‐cinnamaldehyde has been reduced (31 %) with low selectivity for the C=O vs. C=C bond (Table S1).

### Photocatalytic TH studies promoted by terpyridine ruthenium complexes

**Figure 3:**
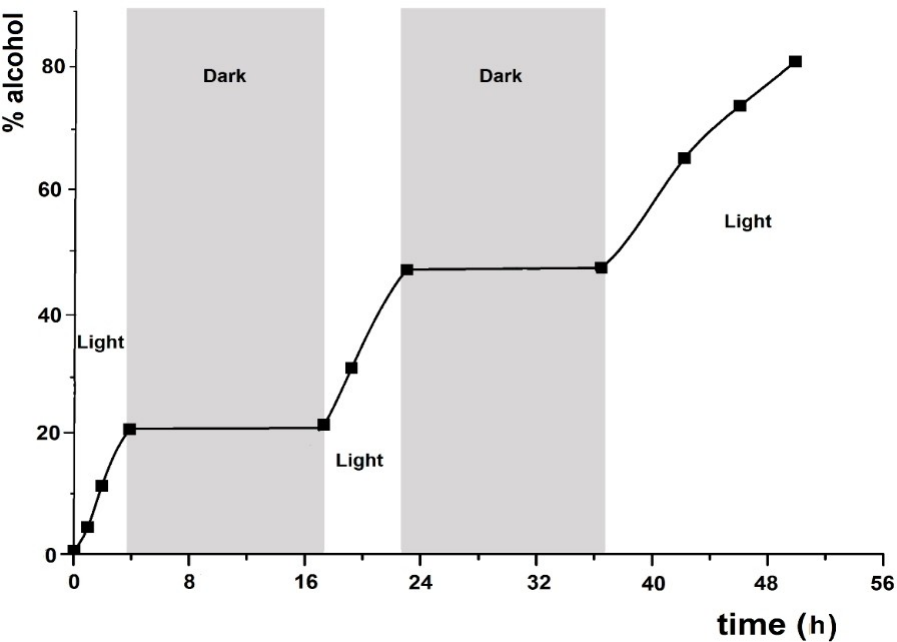
TH of acetophenone **a** (0.1 M) in 2‐propanol with **2** (S/C=5000) and NaO*i*Pr (2 mol%) at 30 °C, over time, with or without light irradiation.

To confirm the catalytic light‐driven process, control experiments have been carried out with the pincer complexes under alternating light and dark conditions. Upon irradiation, the conversion of **a** to 1‐phenylethanol with **2** (S/C=5000) in 2‐propanol at 30 °C increases linearly, while in the dark the alcohol is formed in tiny amount (<1%), resulting in a clear “on/off” process and indicating that the pincer photocatalyst is active for days (Figure 3).

**Figure 4:**
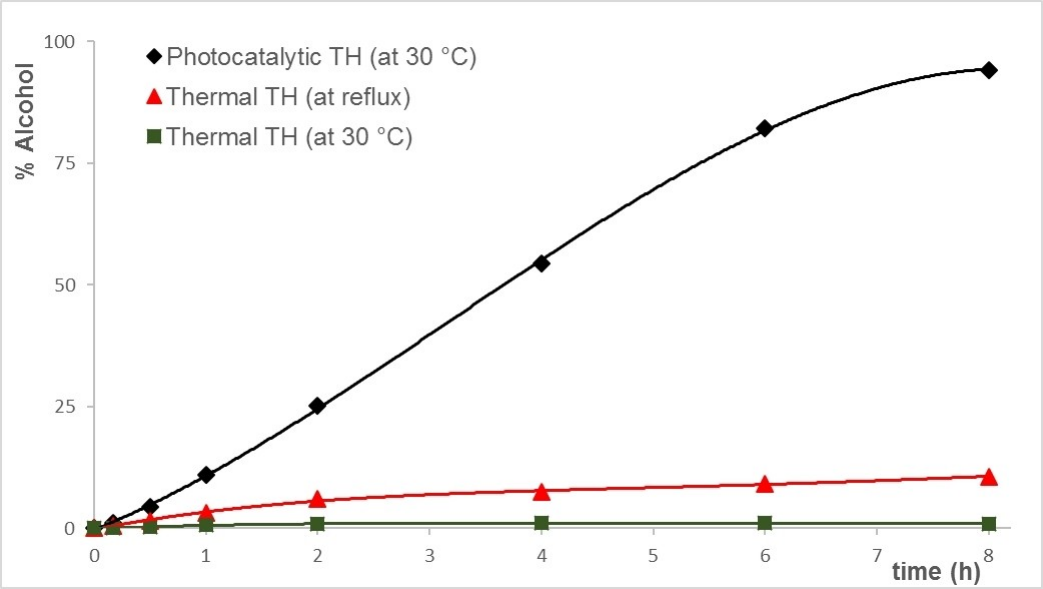
Comparison between the photocatalytic and thermal TH of acetophenone **a** (0.1 M) catalyzed by **2** (S/C=1000) and 2 mol% NaO*i*Pr.

After an induction period of about 15 min in which the color of the reaction mixture changes from orange to dark purple and finally to light yellow under light (Figure S39 in the Supporting Information), the reduction of **a** with **2** (S/C=1000) follows a zero order kinetic till about 80 % conversion and with complete formation of 1‐phenylethanol in 8 h. Conversely, in the dark at 30 °C the conversion is less than 2 % while at refluxing conditions (82 °C) only 11 % of alcohol is formed in 8 h (Figure 4).

**Scheme 3:**
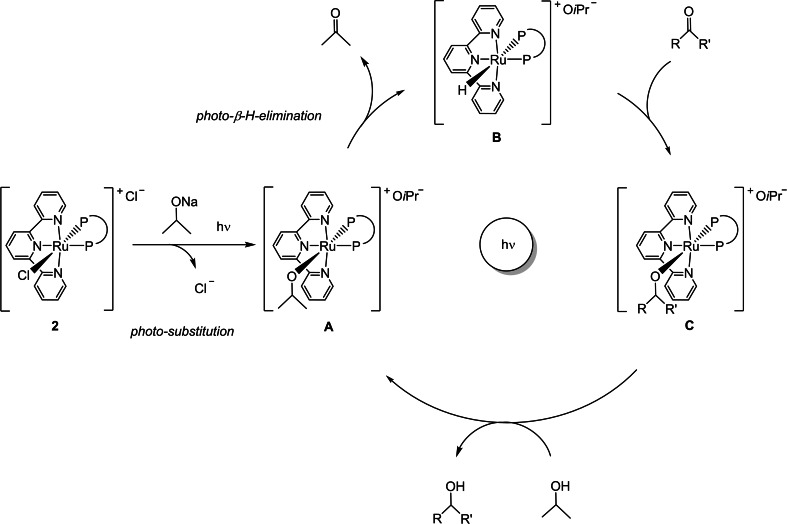
Proposed mechanism for the photocatalytic TH of carbonyl compounds promoted by the terpyridine ruthenium complexes.

To shed light on the steps involved in the photocatalysis, NMR studies have been carried out on the single reactions. Treatment of **2** with NaOiPr (3 equiv.) in 2‐propanol‐*d* ^8^ at room temperature in the dark leads to a tiny amount of the ruthenium isopropoxide [Ru(O*i*Pr)((*R*,*R*)‐Skewphos)(tpy)](O*i*Pr) (**A**) species (Figure S45). Upon irradiation (30 min.) the complex **A** forms quantitatively through photo‐displacement of the chloride and has been characterized by NMR in solution (Scheme 3 and Figure S46–S49).

The alkoxide **A** shows two doublets at *δ*_P_=50.5 and 38.4 ppm (^2^ *J*(P,P)=35.2 Hz) for the P atoms *trans* to the O and N atoms, whereas the terminal tpy H6 and H6” resonances are at *δ*_H_=8.80 and 6.70 ppm. In addition, the CH and CH_3_ signals of the RuO*i*Pr moiety are at *δ*_H_=3.93, 1.14 ppm and *δ*_C_=63.1 and 25.3 ppm, respectively, as inferred from ^1^H‐^13^C HSQC NMR measurements (Figure S50–S51). After a longer irradiation period (>2 h) the red‐orange solution of **A** turns dark brown, affording the ruthenium hydride [RuH((*R*,*R*)‐Skewphos)(tpy)](O*i*Pr) (**B**), through a light‐induced *β*‐hydrogen elimination with extrusion of acetone, in the presence of uncharacterized species (Scheme 3). The ^1^H NMR spectrum of **B** reveals a doublet of doublets at *δ*=−7.49 ppm with ^2^ *J*(H,P)=78.2 and 25.3 Hz, for two P atoms *trans* and *cis* to the hydride, in agreement with the related CNN pincer ruthenium hydride complexes containing a diphosphine,^[39]^ and the peaks at *δ*_H_ = 8.30, 8.27, 8.11 and 7.86 ppm for tpy ligand (Figure S53–S54). NMR experiments performed in 2‐propanol/toluene‐*d* ^8^ (1/1 in volume) as solvent leads to similar results, with formation of **B** in lower amount (Figure S55–S56) and attempts to isolate the hydride **B** by treatment of **2** with NaO*i*Pr in 2‐propanol failed. Reaction of the substrate **b** (1 equiv.) with **B** in 2‐propanol/ toluene‐*d* ^8^ (1/1 in volume), followed by irradiation for 1 h, leads to the quantitative conversion to benzhydrol and acetone in the presence of **B**. Addition of a second equivalent of **b** give complete reduction after 1 h under light, indicating that the hydride **B** is involved in the photocatalytic TH (Figure S57‐S58). Finally, irradiation of **b** with **B** in 2‐propanol‐*d* ^8^ leads to partial deuteration of benzhydrol at the C_α_ position, via the formation of a RuD species (Figure S59‐S64).

Based on these results, it is likely that photocatalytic TH with the pincer complexes involves a light‐driven substitution of the chloride with formation of the O*i*Pr derivative **A**, followed by a *β*‐H‐elimination of acetone, a process which is also induced by light (photo‐*β*‐H‐elimination), leading to ruthenium hydride **B**.^[40]^ Subsequent reduction of the carbonyl compound under irradiation affords the alkoxide **C** which is protonated by 2‐propanol with formation of the alcohol product and the isopropoxide **A** that closes the cycle (Scheme 3). It is worth noting that under thermal conditions the C−H activation of the metal‐alkoxides (type **A**) generally requires a *cis* vacant site,^[41]^ even though a facile *β*‐hydrogen elimination in 18‐electron Ir^III^ complexes through hydrogen bonding with the alcohol media has been claimed by Milstein.^[42]^ Under light irradiation we have found that the 18‐electron complex **A** undergoes a *β*‐hydrogen elimination and that the metal‐hydride **B** is involved in the ketone reduction, possibly through the formation of a Ru‐alkoxide, on account of the microscopic reversibility. The asymmetric TH of **a** with chiral pincer derivatives (**2**‐**5**) in methanol/2‐propanol can be ascribed to the favorable chiral environment of the photocatalyst possibly by π‐stacking interaction of the aromatic rings. The asymmetric TH of acetophenone by pincer ruthenium complexes indicates that this process occurs through a well‐defined chiral photocatalyst without dissociation of the NNN and PP ligands. Therefore, this represents a rare example of visible light‐induced transition metal catalysis with ruthenium, which combines the catalyst‐substrate interaction with the photoinduced processes.

## Conclusions

In summary, the cationic terpyridine diphosphine ruthenium complexes [RuCl(diphosphine)(tpy)]X (X=Cl and PF_6_) have been easily prepared in high yield through a one‐pot synthesis from [RuCl_2_(PPh_3_)_3_], a diphosphine, terpyridine (tpy) and additional NaPF_6_. By using the chiral diphosphines Skewphos, Josiphos and BINAP, single stereoisomers are formed. The reported tpy ruthenium complexes display high catalytic activity in the transfer hydrogenation (TH) of ketones and aldehydes to alcohols at 30 °C induced by light irradiation, using 2‐propanol as the only hydrogen donor. These pincer complexes allow a remarkably high S/C up to 5000 and rate (TOFs up to 264 h^−1^), while poor activity is found under thermal conditions. The chiral complexes [RuCl((*R*,*R*)‐Skewphos)(tpy)]X (X=Cl and PF_6_) catalyze the TH of acetophenone in methanol/2‐propanol, affording (*S*)‐1‐phenylethanol in 52 % *ee*, while the enantiomers [RuCl((*S*,*S*)‐Skewphos)(tpy)]X give the *R* alcohol. This is the first example of asymmetric catalytic TH of a ketone driven by light, indicating that this reaction occurs at a well‐defined and robust visible light‐induced ruthenium catalyst. For these tpy complexes, photo‐dissociation and photo‐*ß*‐H‐elimination reactions have been observed and are likely to occur during catalysis. Studies are ongoing to rationalize the mechanism of the photocatalytic reduction and to apply these catalysts in other C−H activation reactions, including asymmetric transformations under light irradiation.

## Experimental Section

**Figure 5:**
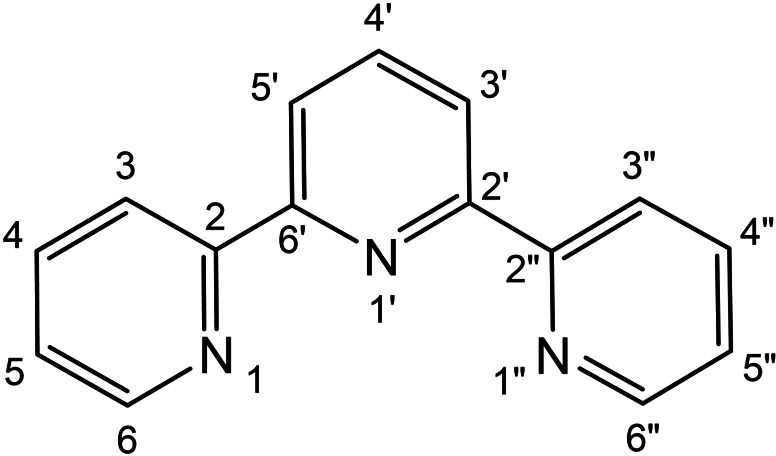
NMR numbering scheme of the tpy ligand in the [RuCl(PP)(tpy)]X complexes.

**General**: All reactions were carried out under an argon atmosphere using standard Schlenk techniques. The solvents were carefully dried by standard methods and distilled under argon before use. The ruthenium complex [RuCl_2_(PPh_3_)_3_]^[43]^ was prepared according to literature procedures, whereas all other chemicals were purchased from Aldrich and Strem and used without further purification. NMR measurements were recorded on an Avance III HD NMR 400 spectrometer. Chemical shifts (ppm) are relative to TMS for ^1^H and ^13^C{^1^H}, whereas H_3_PO_4_ was used for ^31^P{^1^H}. The atom‐numbering scheme for the NMR assignment of terpyridine ligand in the ruthenium complexes is presented in Figure 5. Elemental analyses (C, H, N) were carried out with a Carlo Erba 1106 analyzer, whereas GC analyses were performed with a Varian CP‐3380 gas chromatograph equipped with a 25 m length MEGADEX‐ETTBDMS‐β chiral column with hydrogen (5 psi) as the carrier gas and flame ionization detector (FID). The injector and detector temperature was 250 °C, with initial T=95 °C ramped to 140 °C at 3 °C/min for a total of 20 min of analysis. The t_R_ of acetophenone was 7.59 min, while the t_R_ of (*R*)‐ and (*S*)‐1‐phenylethanol were 10.49 min and 10.76 min, respectively.

**Synthesis of [RuCl(dppp)(tpy)]Cl (1)**: [RuCl_2_(PPh_3_)_3_] (100.0 mg, 0.104 mmol) and dppp (43.9 mg, 0.106 mmol, 1.02 equiv.) were suspended in anhydrous 1‐butanol (5 mL), and stirred at 90 °C for 4 h, until a light‐yellow precipitate of the PPh_3_‐diphosphine mixed species was formed.^[29]^ The ligand tpy (26.0 mg, 0.111 mmol, 1.07 equiv.) was added and the mixture was heated at reflux for 12 h until a dark‐red solution was formed. The solvent was evaporated under reduced pressure and the residue was dissolved in dichloromethane (1 mL) and stirred for 30 min. Addition of diethyl ether (10 mL) afforded the precipitation of the complex as a red solid that was filtered, washed with diethyl ether (5×10 mL), *n*‐pentane (2×10 mL) and dried under reduced pressure. Yield: 74 mg (87 %); ^1^H NMR (400.1 MHz, CD_2_Cl_2_, 25 °C): *δ*=8.29 (d, ^3^ *J*(H,H)=8.1 Hz, 2H; tpy (H3’, H5’)), 8.20 (dd, ^3^ *J*(H,H)=8.4 Hz, ^4^ *J*(H,H)=0.9 Hz, 2H; tpy (H3, H3”)), 8.05 (tt, ^3^ *J*(H,H)=8.8 Hz, ^4^ *J*(H,H)=1.3 Hz, 4H; Ph), 7.98 (t, ^3^ *J*(H,H)=8.1 Hz, 1H; tpy (H4’)), 7.83 (td, ^3^ *J*(H,H)=7.7 Hz, ^4^ *J*(H,H)=1.0 Hz, 2H; tpy (H4, H4”)), 7.80 (d, ^3^ *J*(H,H)=6.1 Hz, 2H; tpy (H6, H6”)), 7.54 (td, ^3^ *J*(H,H)=7.4 Hz, ^4^ *J*(H,H)=1.3 Hz, 2H; Ph), 7.46 (td, ^3^ *J*(H,H)=7.5 Hz, ^4^ *J*(H,H)=1.8 Hz, 4H; Ph), 7.24 (td, ^3^ *J*(H,H)=7.3 Hz, ^4^ *J*(H,H)=1.2 Hz, 2H; Ph), 7.08 (ddd, ^3^ *J*(H,H)=7.2 Hz, ^4^ *J*(H,H)=5.6 Hz, ^5^ *J*(H,H)=1.2 Hz, 2H; tpy (H5, H5”)), 6.96 (td, ^3^ *J*(H,H)=8.0 Hz, ^4^ *J*(H,H)=2.1 Hz, 4H; Ph), 6.58 (ddd, ^3^ *J*(H,H)=8.5 Hz, ^4^ *J*(H,H)=7.3 Hz, ^5^ *J*(H,H)=1.1 Hz, 4H; Ph), 3.02 (*pseudo‐*q, *J*(H,H)=8.2 Hz, 2H; PCH_2_), 2.66‐2.46 (m, 2H; CH_2_), 2.30 ppm (m, 2H; PCH_2_); ^13^C{^1^H} NMR (100.6 MHz, CD_2_Cl_2_, 25 °C): *δ*=158.9 (d, ^3^ *J*(C,P)=2.2 Hz; *ipso* tpy (C2, C2”)), 156.7 (s; tpy (C6, C6”)), 155.0 (s; *ipso* tpy (C2’, C6’)), 137.9 (s; tpy (C4, C4”)), 137.4 (s; tpy (C4’)), 137.5‐128.2 (m; Ph), 126.3 (s; tpy (C5, C5”)), 124.2 (s; tpy (C3, C3”)), 122.9 (s; tpy (C3’, C5’)), 30.2 (dd, ^1^ *J*(C,P)=26.0 Hz, ^3^ *J*(C,P)=4.0 Hz; P*C*H_2_), 23.7 (dd, ^1^ *J*(C,P)=31.2 Hz, ^3^ *J*(C,P)=4.0 Hz; P*C*H_2_), 20.4 ppm (br s; *C*H_2_); ^31^P{^1^H} NMR (162.0 MHz, CD_2_Cl_2_, 25 °C): *δ*=34.1 (d, ^2^ *J*(P,P)=39.0 Hz), 20.0 ppm (d, ^2^ *J*(P,P)=39.0 Hz); elemental analysis calcd (%) for C_42_H_37_Cl_2_N_3_P_2_Ru (817.70): C 61.69, H 4.56, N 5.14; found: C 61.75, H 4.60, N 5.20.

**Synthesis of [RuCl(dppp)(tpy)]PF_6_ (1 a)**: [RuCl(dppp)(tpy)]Cl (70.0 mg, 0.086 mmol) was dissolved in methanol (1 mL), NaPF_6_ (40.0 mg, 0.238 mmol, 2.77 equiv.) was added and the mixture was stirred at room temperature for 2 h until a red precipitate was formed. The solid was filtered, washed with diethyl ether (2×5 mL), *n*‐pentane (2×5 mL) and dried under reduced pressure. Yield 74.2 mg (93 %); ^1^H NMR (400.1 MHz, CD_2_Cl_2_, 25 °C): *δ*=8.12‐7.97 (m, 6H; tpy (H3’, H5’)), tpy (H3, H3”), Ph), 7.93 (d, ^3^ *J*(H,H)=8.0 Hz, 2H; tpy (H6, H6”)), 7.85 (t, ^3^ *J*(H,H)=7.8 Hz, 1H; tpy (H4’)), 7.82‐7.78 (m, 2H; tpy (H4, H4”)), 7.59–7.52 (m, 2H; Ph), 7.51‐7.42 (m, 4H; Ph), 7.28 (t, ^3^ *J*(H,H)=7.4 Hz, 2H; Ph), 7.15‐7.04 (m, 2H; tpy (H5, H5”)), 7.00 (td, ^3^ *J*(H,H)=8.0 Hz, ^4^ *J*(H,H)=2.1 Hz, 4H; Ph), 6.92‐6.79 (m, 2H; Ph), 6.59 (t, ^3^ *J*(H,H)=8.8 Hz, 4H; Ph), 2.97 (*pseudo‐*q, *J*(H,H)=8.0 Hz, 2H; PCH_2_), 2.68‐2.46 (m, 2H; CH_2_), 2.23 ppm (m, 2H; PCH_2_); ^31^P{^1^H} NMR (162.0 MHz, CD_2_Cl_2_, 25 °C): *δ*=34.1 (d, ^2^ *J*(P,P)=39.1 Hz), 19.6 (d, ^2^ *J*(P,P)=39.1 Hz), −144.4 ppm (hept, ^1^ *J*(P,F)=711.8 Hz; *P*F_6_); elemental analysis calcd (%) for C_42_H_37_ClF_6_N_3_P_3_Ru (927.21): C 54.41, H 4.02, N 4.53; found: C 54.45, H 4.0, N 4.60.

**Synthesis of [RuCl((*R***, ***R*** **)‐Skewphos)(tpy)]Cl (2)**: [RuCl_2_(PPh_3_)_3_] (100.0 mg, 0.104 mmol) and (*R*,*R*)‐Skewphos (47.0 mg, 0.107 mmol, 1.03 equiv.) were suspended in anhydrous 1‐butanol (5 mL) and stirred at 90 °C for 4 h, until an orange precipitate was formed. The ligand tpy (25.0 mg, 0.107 mmol, 1.03 equiv.) was added and the mixture was heated at reflux for 12 h until a red solution was formed. The solvent was evaporated under reduced pressure, and the residue was dissolved in dichloromethane (1 mL) and stirred at RT for 30 min. Addition of diethyl ether (10 mL) afforded the precipitation of the complex as a red solid that was filtered, washed with of diethyl ether (5×10 mL), *n*‐pentane (2×10 mL) and dried under reduced pressure. Yield: 77.5 mg (88 %); ^1^H NMR (400.1 MHz, CD_2_Cl_2_, 25 °C): *δ*=8.83 (d, ^3^ *J*(H,H)=5.7 Hz, 1H; tpy (H6)), 8.39 (ddd, ^3^ *J*(H,H)=7.1 Hz, ^4^ *J*(H,H)=2.1 Hz, ^5^ *J*(H,H)=1.1 Hz, 1H; tpy (H3’)), 8.14 (dd, ^3^ *J*(H,H)=8.1 Hz, ^4^ *J*(H,H)=1.5 Hz, 1H; tpy (H3”)), 8.06 (br t, ^3^ *J*(H,H)=8.0 Hz, 1H; Ph), 8.03–7.97 (m, 2H; tpy (H4’) and (H3)), 7.97‐7.91 (m, 2H; tpy (H4) and (H5’)), 7.90‐7.58 (m, 4H; Ph), 7.62 (td, 1H, ^3^ *J*(H,H)=7.7 Hz, ^4^ *J*(H,H)=1.5 Hz; tpy (H4”)), 7.47 (td, ^3^ *J*(H,H)=7.7 Hz, ^4^ *J*(H,H)=2.0 Hz, 1H; Ph), 7.41–7.24 (m, 6H; Ph and tpy (H5)), 7.16 (m, 1H; Ph), 7.08 (td, ^3^ *J*(H,H)=7.9 Hz, ^3^ *J*(H,H)=2.1 Hz, 2H; Ph), 6.99 (ddd, ^3^ *J*(H,H)=9.5 Hz, ^4^ *J*(H,H)=8.2 Hz, ^5^ *J*(H,H)=1.4 Hz, 2H; Ph), 6.88 (td, ^3^ *J*(H,H)=8.1 Hz, ^4^ *J*(H,H)=2.3 Hz, 2H; Ph), 6.77 (ddd, ^3^ *J*(H,H)=7.4 Hz, ^4^ *J*(H,H)=5.8 Hz, ^5^ *J*(H,H)=1.5 Hz, 1H; tpy (H5”)), 6.63 (d, ^3^ *J*(H,H)=5.7 Hz, 1H; tpy (H6”)), 6.29 (td, ^3^ *J*(H,H)=8.6 Hz, ^4^ *J*(H,H)=1.4 Hz, 2H; Ph), 3.84‐3.70 (m, 1H; PC*H*CH_3_), 2.82 (dtt, ^2^ *J*(H,H)=15.0 Hz, ^3^ *J*(H,P)=11.3 Hz, ^3^ *J*(H,H)=3.7 Hz; 1H; CHC*H*_2_), 2.61–2.49 (m, 1H; PC*H*CH_3_), 2.27–2.04 (m, 1H; CHC*H*_2_), 1.44 (dd, ^3^ *J*(H,P)=12.1 Hz, ^3^ *J*(H,H)=7.5 Hz, 3H; CHC*H*_3_), 0.63 ppm (dd, ^3^ *J*(H,P)=12.1 Hz, ^3^ *J*(H,H)=6.8 Hz, 3H; CHC*H*_3_); ^13^C{^1^H} NMR (100.6 MHz, CD_2_Cl_2_, 25 °C): *δ*=158.9 (d, ^3^ *J*(C,P)=2.8 Hz; tpy (C6)), 158.8 (d, ^3^ *J*(C,P)=2.2 Hz; *ipso* tpy (C2)), 158.0 (d, ^3^ *J*(C,P)=2.6 Hz; *ipso* tpy (C2’)), 155.4 (s; *ipso* tpy (C2”)), 155.1 (s; tpy (C6”)), 154.7 (s; *ipso* tpy (C6’)), 141.8 (d, ^1^ *J*(C,P)=35.6 Hz; ipso Ph), 137.8 (s; tpy (C4”)), 137.6 (s; tpy (C4)), 137.2 (s; tpy (C4’)), 135.1–126.9 (m; Ph), 126.2 (s; tpy (C5)), 125.4 (s; tpy (C5”)), 124.6 (s; tpy (C5’)), 123.4 (s; tpy (C3)), 123.0 (s; tpy (C3”)), 122.6 (s; tpy (C3’)), 37.3 (t, ^2^ *J*(C,P)=6.1 Hz; CH*C*H_2_), 33.3 (d, ^1^ *J*(C,P)=22.2 Hz; P*C*HCH_3_), 20.1 (dd, ^1^ *J*(C,P)=27.6 Hz, ^3^ *J*(C,P)=4.7 Hz; P*C*HCH_3_), 19.0 (d, ^2^ *J*(C,P)=6.4 Hz; PCH*C*H_3_), 18.0 ppm (br s; PCH*C*H_3_); ^31^P{^1^H} NMR (162.0 MHz, CD_2_Cl_2_, 25 °C): *δ*=49.3 (d, ^2^ *J*(P,P)=38.5 Hz), 33.9 ppm (d, ^2^ *J*(P,P)=38.5 Hz); elemental analysis calcd (%) for C_44_H_41_Cl_2_N_3_P_2_Ru (845.75): C 62.49, H 4.89, N 4.97; found: C 62.45, H 4.90, N 5.00.

**Synthesis of [RuCl((*R***, ***R*** **)‐Skewphos)(tpy)]PF_6_ (2 a)**: [RuCl((*R*,*R*)‐Skewphos)(tpy)]Cl (75.0 mg, 0.089 mmol) was dissolved in methanol (0.5 mL) and NaPF_6_ (45.0 mg, 0.268 mmol, 3.01 equiv.) was added. The mixture was stirred at room temperature for 20 min and the red precipitate was filtered, washed with diethyl ether (3×5 mL), *n*‐pentane (2×5 mL) and dried under reduced pressure. Yield 82.5 mg (97 %); ^1^H NMR (400.1 MHz, CD_2_Cl_2_, 25 °C): *δ*=8.86 (dd, ^3^ *J*(H,H)=5.7 Hz, ^4^ *J*(H,H)=1.5 Hz, 1H; tpy (H6)), 8.11–8.04 (m, 3H; tpy (H3), (H3”) and Ph), 7.96 (t, ^3^ *J*(H,H)=8.0 Hz, 1H; Ph), 7.90 (td, ^3^ *J*(H,H)=7.8 Hz, ^4^ *J*(H,H)=1.4 Hz, 1H; tpy (H4’)), 7.86–7.74 (m, 3H; tpy (H4), (H3) and (H5’)), 7.73–7.64 (m, 3H; Ph), 7.60 (td, ^3^ *J*(H,H)=7.7 Hz, ^4^ *J*(H,H)=1.5 Hz, 1H; tpy (H4”)), 7.51–7.46 (m, 1H; Ph), 7.42–7.26 (m, 7H; Ph and tpy (H5)), 7.09 (td, ^3^ *J*(H,H)=7.8 Hz, ^3^ *J*(H,H)=2.2 Hz, 2H; Ph), 7.00 (ddd, ^3^ *J*(H,H)=9.5 Hz, ^4^ *J*(H,H)=8.2 Hz, ^5^ *J*(H,H)=1.4 Hz, 2H; Ph), 6.92–6.86 (m, 2H; Ph), 6.80 (ddd, ^3^ *J*(H,H)=7.5 Hz, ^4^ *J*(H,H)=5.8 Hz, ^5^ *J*(H,H)=1.5 Hz, 1H; tpy (H5”)), 6.66 (dd, ^3^ *J*(H,H)=5.8 Hz, ^4^ *J*(H,H)=1.6 Hz, 1H; tpy (H6”)), 6.29 (td, ^3^ *J*(H,H)=8.6 Hz, ^4^ *J*(H,H)=1.3 Hz, 2H; Ph), 3.86–3.70 (m, 1H; PC*H*CH_3_)), 2.82 (ddt, 1H, ^2^ *J*(H,H)=15.1 Hz, ^3^ *J*(H,P)=11.3 Hz, ^3^ *J*(H,H)=3.6 Hz; 1H; CHC*H*_2_), 2.60–2.49 (m, 1H; PC*H*CH_3_), 2.28–2.04 (m, 1H; CHC*H*_2_), 1.44 (dd, ^3^ *J*(H,P)=12.1 Hz, ^3^ *J*(H,H)=7.5 Hz, 3H; CHC*H*_3_), 0.64 ppm (dd, ^3^ *J*(H,P)=12.2 Hz, ^3^ *J*(H,H)=6.8 Hz, 3H; CHC*H*_3_); ^31^P{^1^H} NMR (162.0 MHz, CD_2_Cl_2_, 25 °C): *δ*=49.0 (d, ^2^ *J*(P,P)=38.8 Hz), 33.8 (d, ^2^ *J*(P,P)=38.8 Hz), −144.4 ppm (hept, ^1^ *J*(P,F)=711.4 Hz; *P*F_6_); elemental analysis calcd (%) for C_44_H_41_ClF_6_N_3_P_3_Ru (955.27): C 55.32, H 4.33, N 4.40; found: C 55.30, H 4.30, N 4.45.

**Synthesis of [RuCl((*S***, ***S*** **)‐Skewphos)(tpy)]Cl (3)**: Complex **3** was prepared following the procedure used for **2** employing (*S*,*S*)‐Skewphos (47.0 mg, 0.107 mmol, 1.03 equiv.) in place of (*R*,*R*)‐Skewphos. Yield: 75.0 mg (85 %). NMR data of **3** were identical to those of the enantiomer **2**; elemental analysis calcd (%) for C_44_H_41_Cl_2_N_3_P_2_Ru (845.75): C 62.49, H 4.89, N 4.97; found: C 62.35, H 4.95, N 4.80.

**Synthesis of [RuCl((*S***, ***S*** **)‐Skewphos)(tpy)]PF_6_ (3 a)**: Complex **3 a** was prepared following the procedure used for **2 a** employing [RuCl((*S*,*S*)‐Skewphos)(tpy)]Cl (**3**) (75.0 mg, 0.089 mmol) in place of [RuCl((*R*,*R*)‐Skewphos)(tpy)]Cl (**2**). Yield 81.0 mg (95 %). NMR data of **3 a** were identical to those of the enantiomer **2 a**; elemental analysis calcd (%) for C_44_H_41_ClF_6_N_3_P_3_Ru (955.27): C 55.32, H 4.33, N 4.40; found: C 55.51, H 4.45, N 4.34.

**Synthesis of [RuCl((*S***, ***R*** **)‐Josiphos)(tpy)]PF_6_ (4 a)**: [RuCl_2_(PPh_3_)_3_] (100.0 mg, 0.104 mmol) and (*S*,*R*)‐Josiphos (80.0 mg, 0.125 mmol, 1.2 equiv.) were suspended in anhydrous toluene (2 mL) and stirred at 105 °C for 4 h. The dark‐red solution was cooled at room temperature and evaporated to dryness under reduced pressure. The residue was dissolved in anhydrous 1‐butanol (5 mL), tpy (25.0 mg, 0.107 mmol, 1.03 equiv.) was added and the mixture was heated at reflux for 12 h until a red solution was formed. The solvent was evaporated under reduced pressure and the residue was dissolved in dichloromethane (1 mL) and stirred at RT for 30 min. Addition of diethyl ether (10 mL) afforded [RuCl((*S*,*R*)‐Josiphos)(tpy)]Cl (**4**) as red‐brown precipitate that was filtered, washed with of diethyl ether (5×10 mL), *n*‐pentane (2×10 mL) and dried under reduced pressure. The product was dissolved in methanol (2 mL) and NaPF_6_ (50.0 mg, 0.298 mmol, 2.90 equiv.) was added. The mixture was stirred at RT for 30 min and the red‐brown precipitate was filtered, washed with diethyl ether (3×5 mL), *n*‐pentane (2×5 mL) and dried under reduced pressure. Yield: 90.0 mg (78 %); ^1^H NMR (400.1 MHz, CD_2_Cl_2_, 25 °C): *δ*=9.42 (br s, 1H; tpy (H6)), 8.52 (d, ^3^ *J*(H,H)=6.6 Hz, 1H; tpy (H3’)), 8.45 (t, ^3^ *J*(H,H)=8.4 Hz, 1H; tpy (H3)), 8.30–8.21 (m, 2H; tpy (H4’) and (H5’)), 8.17 (t, ^3^ *J*(H,H)=7.9 Hz, 1H; tpy (H4)), 8.11 (d, ^3^ *J*(H,H)=7.9 Hz, 1H; tpy (H3”)), 8.03 (t, ^3^ *J*(H,H)=8.8 Hz, 2H; Ph), 7.76 (t, ^3^ *J*(H,H)=7.7 Hz, 1H; tpy (H4”)), 7.52–7.44 (m, 2H; Ph), 7.41–7.32 (m, 4H; Ph and tpy (H5)), 7.20–7.06 (m, 3H; Ph), 6.72 (t, ^3^ *J*(H,H)=6.6 Hz, 1H; tpy (H5”)), 6.41 (br s, 1H; tpy (H6”)), 5.14 (br s, 1H; C_5_H_3_), 4.87 (br s, 1H; C_5_H_3_), 4.81 (br t, *J*(H,H)=2.2 Hz, 1H; C_5_H_3_), 3.81 (br s, 5H; C_5_H_5_), 3.45 (m, 1H; C*H*CH_3_), 2.02 (dd, ^2^ *J*(H,P)=24.3 Hz, ^3^ *J*(H,H)=11.5 Hz, 1H; C*H* of Cy)), 1.63–−0.18 ppm (m, 24H; CH, CH_2_ (Cy) and CHC*H*_3_); ^13^C{^1^H} NMR (100.6 MHz, CD_2_Cl_2_, 25 °C): *δ*=160.9 (br s; *ipso* tpy (C2)), 160.0 (br s; tpy (C6)), 158.3 (s; *ipso* tpy (C2’)), 157.1 (s; *ipso* tpy (C2”)), 156.9 (s; *ipso* tpy (C6’)), 154.5 (s; tpy (C6”)), 138.3 (s; tpy (C4)), 137.9 (br s; ipso Ph), 137.6 (br s; tpy (C4’) and (C4”)), 136.6–126.9 (m; Ph), 126.0 (s; tpy (C5”)), 125.8 (s; tpy (C5)), 123.8 (s; tpy (C3)), 123.1 (s; tpy (C3”)), 122.3 (s; tpy (C5’)), 122.2 (s; tpy (C3’)), 98.6 (m; *ipso*‐C_5_H_3_), 75.9 (s; C_5_H_3_), 70.7 (s; C_5_H_3_), 70.5 (br s; C_5_H_5_), 70.0 (d, *J*(C,P)=6.5 Hz; C_5_H_3_), 37.7 (d, ^1^ *J*(C,P)=39.3 Hz; *C*H of Cy), 37.3 (d, ^1^ *J*(C,P)=36.4 Hz; *C*H of Cy), 30.8 (s; *C*H_2_ of Cy), 29.4 (d, *J*(C,P)=13.1 Hz; *C*H_2_ of Cy), 27.9 (d, *J*(C,P)=14.6 Hz; *C*H_2_ of Cy), 27.4 (d, ^1^ *J*(C,P)=23.3 Hz; P*C*HCH_3_), 27.2–26.8 (m; *C*H_2_ of Cy), 26.6–26.2 (m; *C*H_2_ of Cy), 25.8 (s; *C*H_2_ of Cy), 15.4 ppm (d, ^1^ *J*(C,P)=13.1 Hz; PCH*C*H_3_); ^31^P{^1^H} NMR (162.0 MHz, CD_2_Cl_2_, 25 °C): *δ*=47.6 (d, ^2^ *J*(P,P)=35.0 Hz), 29.0 (d, ^2^ *J*(P,P)=35.0 Hz), −144.4 ppm (hept, ^1^ *J*(P,F)=710.0 Hz; *P*F_6_).

^1^H NMR (400.1 MHz, CD_3_OD, 25 °C): *δ*=9.39 (br s, 1H; tpy (H6)), 8.82 (d, ^3^ *J*(H,H)=8.1 Hz, 1H; tpy (H3’)), 8.76 (dd, ^3^ *J*(H,H)=8.2 Hz, ^4^ *J*(H,H)=1.1 Hz, 1H; tpy (H3)), 8.57 (d, ^3^ *J*(H,H)=8.0 Hz, 1H; tpy (H5’)), 8.42–8.34 (m, 2H; tpy (H3”) and (H4)), 8.26 (t, ^3^ *J*(H,H)=7.5 Hz, 1H tpy (H4’)), 8.07 (br t, ^3^ *J*(H,H)=8.7 Hz, 2H; Ph), 7.86 (td, ^3^ *J*(H,H)=7.9 Hz, ^4^ *J*(H,H)=1.2 Hz, 1H; tpy (H4”)), 7.53–7.43 (m, 3H; Ph and tpy (H5)), 7.34 (br t, ^3^ *J*(H,H)=6.9 Hz, 2H: Ph), 7.31–7.24 (m, 1H; Ph), 7.22–7.15 (m, 2H; Ph), 7.11 (br t, ^3^ *J*(H,H)=7.5 Hz, 1H; Ph), 6.80 (dt, ^3^ *J*(H,H)=6.8 Hz, 1.0 Hz, 1H; tpy (H5”)), 6.45 (br s, 1H; tpy (H6”)), 5.26 (br s, 1H; C_5_H_3_), 4.97 (br t, *J*(H,H)=2.7 Hz, 1H; C_5_H_3_), 4.86 (br s, 1H; C_5_H_3_ overlapped with H_2_O signal), 3.85 (br s, 5H; C_5_H_5_), 3.55–3.46 (m, 1H; C*H*CH_3_), 2.09 (dd, ^2^ *J*(H,P)=24.1 Hz, ^3^ *J*(H,H)=11.6 Hz, 1H; CH of Cy)), 1.66–−0.14 (m, 24H; CH, CH_2_ of Cy and CHC*H*_3_); ^13^C{^1^H} NMR (100.6 MHz, CD_3_OD, 25 °C): *δ*=161.2 (br s; *ipso* tpy (C2)), 160.1 (d, ^3^ *J*(C,P)=4.8 Hz; tpy (C6)), 158.7 (br s; *ipso* tpy (C2’)), 157.4 (s; *ipso* tpy (C2”)), 157.2 (s; *ipso* tpy (C6’)), 154.4 (s; tpy (C6”)), 138.6 (s; tpy (C4’)), 138.5 (br s; ipso Ph), 138.0 (br s; ipso Ph), 137.9 (s; tpy (C4)), 137.8 (s; tpy (C4”)), 137.0–126.4 (m; Ph), 126.1 (s; tpy (C5)), 126.0 (s; tpy (C5”)), 124.0 (s; tpy (C3)), 123.4 (s; tpy (C3”)), 122.9 (d, ^4^ *J*(C,P)=1.5 Hz; tpy (C5’)), 122.7 (d, ^4^ *J*(C,P)=1.7 Hz; tpy (C3’)), 95.1 (dd, ^1^ *J*(C,P)=21.6 Hz, ^3^ *J*(C,P)=3.4 Hz; *ipso*‐C_5_H_3_), 76.0 (s; C_5_H_3_), 70.6 (d, *J*(C,P)=7.3 Hz; C_5_H_3_), 70.4 (br s; C_5_H_5_), 69.7 (d, *J*(C,P)=4.4 Hz; C_5_H_3_), 37.5 (d, ^1^ *J*(C,P)=20.2 Hz; *C*H of Cy), 37.3 (d, ^1^ *J*(C,P)=18.2 Hz; *C*H of Cy), 30.7 (s; *C*H_2_ of Cy), 29.2 (d, *J*(C,P)=6.6 Hz; *C*H_2_ of Cy), 27.6 (d, *J*(C,P)=8.1 Hz; *C*H_2_ of Cy), 27.5 (d, ^1^ *J*(C,P)=5.9 Hz; P*C*HCH_3_), 27.0 (s; *C*H_2_ of Cy), 26.7 (d, *J*(C,P)=9.5 Hz; *C*H_2_ of Cy), 26.5 (d, *J*(C,P)=10.3 Hz; *C*H_2_ of Cy), 26.4–26.0 (m; *C*H_2_ of Cy), 25.6 (s; *C*H_2_ of Cy), 14.6 ppm (d, ^1^ *J*(C,P)=7.3 Hz; PCH*C*H_3_); ^31^P{^1^H} NMR (162.0 MHz, CD_3_OD, 25 °C): *δ*=49.2 (d, ^2^ *J*(P,P)=33.3 Hz), 28.9 (d, ^2^ *J*(P,P)=33.3 Hz), −144.3 ppm (hept, ^1^ *J*(P,F)=708.8 Hz; *P*F_6_); elemental analysis calcd (%) for C_51_H_55_ClF_6_FeN_3_P_3_Ru (1109.30): C 55.22, H 5.00, N 3.79; found: C 55.20, H 5.05, N 3.85.

**NMR data for compound 4**: ^1^H NMR (400.1 MHz, CD_2_Cl_2_, 25 °C): *δ*=9.37 (br s, 1H; tpy (H6)), 8.86 (d, ^3^ *J*(H,H)=7.9 Hz, 1H; tpy (H3’)), 8.80 (d, ^3^ *J*(H,H)=8.0 Hz, 1H; tpy (H3)), 8.59 (d, ^3^ *J*(H,H)=7.9 Hz, 1H; tpy (H4’)), 8.41 (d, 1H, ^3^ *J*(H,H)=7.9 Hz, 1H; tpy (H5’)), 8.25 (m, 2H, tpy (H4) and (H3”)), 8.05 (t, ^3^ *J*(H,H)=8.6 Hz, 2H; Ph), 7.78 (t, ^3^ *J*(H,H)=8.2 Hz, 1H; tpy (H4”)), 7.56–7.22 (m, 7H; Ph and tpy (H5)), 7.19–7.07 (m, 2H; Ph), 6.68 (t, ^3^ *J*(H,H)=6.7 Hz, 1H; tpy (H5”)), 6.38 (br s, 1H; tpy (H6”)), 5.13 (br s, 1H; C_5_H_3_), 4.85 (br s, 1H; C_5_H_3_), 4.80 (br s, 1H; C_5_H_3_), 3.80 (br s, 5H; C_5_H_5_), 3.45 (m, 1H; C*H*CH_3_), 2.05 (m, 1H; C*H* of Cy)), 1.68–−0.20 ppm (m, 24H; CH, CH_2_ (Cy) and CHC*H*_3_); ^31^P{^1^H} NMR (162.0 MHz, CD_2_Cl_2_, 25 °C): *δ*=47.9 (d, ^2^ *J*(P,P)=34.4 Hz), 29.2 ppm (d, ^2^ *J*(P,P)=34.4 Hz).

**Synthesis of [RuCl((*R*)‐BINAP)(tpy)]PF_6_ (5 a)**: [RuCl_2_(PPh_3_)_3_] (100.0 mg, 0.104 mmol) and (*R*)‐BINAP (78.0 mg, 0.125 mmol, 1.2 equiv.) were suspended in dichloromethane (10 mL), and stirred at room temperature for 12 h. The obtained orange solution was concentrated to almost 1 mL by evaporation of the solvent under reduced pressure. Addition of diethyl ether (10 mL) afforded the precipitation of [RuCl_2_((*R*)‐BINAP)(PPh_3_)]^[44]^ as an orange‐brown solid that was washed with diethyl ether (3×10 mL) and *n*‐pentane (2×10 mL) to remove the excess of the diphosphine. The solid was dissolved in anhydrous 1‐butanol (5 mL), tpy (25.0 mg, 0.107 mmol, 1.03 equiv.) was added and the mixture was stirred at reflux for 12 h until a red solution was formed. The solvent was evaporated under reduced pressure, the residue was dissolved in dichloromethane (1 mL) and stirred at RT for 30 min. Addition of diethyl ether (10 mL) afforded [RuCl((*R*)‐BINAP)(tpy)]Cl (**5**) as orange‐red precipitate that was filtered, washed with diethyl ether (5×10 mL), *n*‐pentane (2×10 mL) and dried under reduced pressure. The product was dissolved in methanol (2 mL), NaPF_6_ (50.0 mg, 0.298 mmol, 2.90 equiv.) was added and the mixture was stirred at RT for 30 min obtaining a red‐orange precipitate that was filtered, washed with diethyl ether (3×5 mL), *n*‐pentane (2×5 mL) and dried under reduced pressure. Yield: 101.7 mg (86 %); ^1^H NMR (400.1 MHz, CD_2_Cl_2_, 25 °C): *δ*=9.43 (d, ^3^ *J*(H,H)=5.7 Hz, 1H; tpy (H6)), 8.19 (dd, ^3^ *J*(H,H)=8.1 Hz, ^4^ *J*(H,H)=1.4 Hz, 1H; naphthyl proton), 8.15 (dd, ^3^ *J*(H,H)=8.1 Hz, ^4^ *J*(H,H)=1.6 Hz, 1H; tpy (H3)), 8.08 (td, ^3^ *J*(H,H)=7.8 Hz, ^4^ *J*(H,H)=1.4 Hz, 1H; tpy (H4)), 8.03–7.84 (m, 5H; naphthyl protons and tpy (H4’, H3’ and H5’)), 7.76–7.60 (m, 5H; naphthyl protons, tpy (H3”) and (H4”)), 7.57–7.49 (m, 2H; tpy (H5) and aromatic proton), 7.48–7.43 (m, 2H; aromatic proton and tpy (H5”)), 7.38 (br t, ^3^ *J*(H,H)=7.4 Hz, 1H; aromatic proton), 7.33–7.24 (m, 2H; aromatic protons), 7.21–7.04 (m, 7H; naphthyl protons and Ph), 6.92–6.73 (m, 4H; naphthyl proton and Ph), 6.52–6.42 (m, 2H; naphthyl protons), 6.37–6.30 (m, 2H; aromatic protons), 6.28–6.20 (m, 3H; aromatic protons and tpy (H6”)), 6.08 (d, ^3^ *J*(H,H)=8.8 Hz, 1H; Ph), 6.02 ppm (t, ^3^ *J*(H,H)=8.6 Hz, 2H; Ph); ^13^C{^1^H} NMR (100.6 MHz, CD_2_Cl_2_, 25 °C): *δ*=160.5 (s; tpy (C6)), 159.8 (s; *ipso* tpy (C2)), 159.6 (br s; *ipso* tpy (C2’)), 158.4 (br s; tpy (C6”)), 156.7 (s; *ipso*‐tpy (C2”)), 155.0 (s; *ipso* tpy (C6’)), 138.8 (s; tpy (C4)), 138.5 (s; tpy (C4’)), 137.0 (s; tpy (C4”)), 135.0–125.9 (m, aromatic carbon atoms), 126.8 (s; tpy (C5), 125.7 (s; tpy (C5”)), 125.0 (s; naphthyl), 123.8 (s; tpy (C3), 122.9 (s; tpy (C3”)), 122.4 (s; tpy (C5’)), 121.3 ppm (s; tpy (C3’)); ^31^P{^1^H} NMR (162.0 MHz, CD_2_Cl_2_, 25 °C): *δ*=40.6 (d, ^2^ *J*(P,P)=30.3 Hz), 39.5 (d, ^2^ *J*(P,P)=30.3 Hz), −144.4 ppm (hept, ^1^ *J*(P,F)=709.7 Hz; *P*F_6_); elemental analysis calcd (%) for C_59_H_43_ClF_6_N_3_P_3_Ru (1137.45): C 62.30, H 3.81, N 3.69; found: C 62.25, H 3.85, N 3.75.

**NMR data for compound 5**: ^1^H NMR (400.1 MHz, CD_2_Cl_2_, 25 °C): *δ*=9.35 (d, ^3^ *J*(H,H)=5.2 Hz, 1H; tpy (H6)), 8.46 (d, ^3^ *J*(H,H)=7.9 Hz, 1H; tpy (H3’)), 8.40 (d, ^3^ *J*(H,H)=7.8 Hz, 1H; tpy (H3)), 8.21 (t, ^3^ *J*(H,H)=8.6 Hz, 2H; naphthyl protons), 8.11 (t, ^3^ *J*(H,H)=7.6 Hz, 1H; tpy (H4)), 8.03–7.86 (m, 4H; naphthyl protons and tpy (H4’ and H5’)), 7.85–7.76 (m, 2H; naphthyl protons), 7.76–7.65 (m, 2H; naphthyl protons), 7.63–7.52 (m, 3H; aromatic proton, tpy (H3”) and (H4”)), 7.52–7.39 (m, 3H; tpy (H5) and aromatic proton), 7.33 (t, ^3^ *J*(H,H)=6.9 Hz, 2H; aromatic protons), 7.31–7.20 (m, 3H; aromatic proton and tpy (H5”)), 7.15–6.97 (m, 6H; naphthyl protons and Ph), 6.91–6.69 (m, 3H; naphthyl proton and Ph), 6.55–6.38 (m, 2H; naphthyl protons), 6.35–6.24 (m, 1H; Ph), 6.24–6.14 (m, 3H; aromatic protons and tpy (H6”)), 6.07 (d, ^3^ *J*(H,H)=8.8 Hz, 1H; Ph), 5.98 ppm (t, ^3^ *J*(H,H)=8.5 Hz, 2H; Ph); ^31^P{^1^H} NMR (162.0 MHz, CD_2_Cl_2_, 25 °C): δ=40.8 (d, ^2^ *J*(P,P)=30.4 Hz), 39.8 ppm (d, ^2^ *J*(P,P)=30.4 Hz).

**Typical procedure for the photocatalytic TH of aldehydes and ketones**: The ruthenium catalyst solution used for the photocatalytic TH was prepared by dissolving the complexes (0.02 mmol) in 2‐propanol (5 mL). The catalyst solution (250 μL, 1.0 μmol) and a 0.1 m solution of NaO*i*Pr (200 μL, 20 μmol) in 2‐propanol were added subsequently to the carbonyl compound solution (1.0 mmol) in 2‐propanol or a 2‐propanol/MeOH (1 : 1 v/v) mixture (final volume 10 mL). The resulting solutions were stirred in a thermostated water bath at 30 °C. Irradiation of the samples was carried out by using a 300 W Xenon Arc Lamp (LSB530 A, LOT‐Oriel, Darmstadt, Germany), emitting in the range 200–2500 nm (solar simulator). Samples were purged with Ar at least 15 minutes before irradiation. The reaction was sampled by removing an aliquot of the reaction mixture, which was quenched by addition of diethyl ether (1 : 1 v/v), filtered over a short silica pad and submitted to GC analysis. The base addition was considered as the start time of the reaction. The S/C molar ratio was 1000/1, whereas the base concentration was 2 mol% respect to the ketone substrates (0.1 m). The same procedure was followed for TH reactions with other S/C (in the range 1000–5000) or with different base concentrations (1–10 mol%), using the appropriate amount of catalysts, base and 2‐propanol.

**X‐ray crystallography**: Single crystals of the complex **3 a** were obtained by slow cooling of a concentrated solution of the species in MeOH, whereas **4 a** crystallizes from CH_2_Cl_2_. X‐ray diffraction data were collected on a Bruker D8 Venture equipped with a CMOS detector (Photon‐100), a Mo TXS rotating anode (*λ*=0.71073 Å), and a Helios optic monochromator (**3 a**) and a Bruker D8 Venture equipped with a CPAD detector (Photon II), a Mo IMS microsource, and a Helios optic monochromator (**4 a**). For additional details for collection and refining of data, see the Supporting Information.

Deposition Numbers 2165467 (for **3 a**), 2165468 (for **4 a**) contain the supplementary crystallographic data for this paper. These data are provided free of charge by the joint Cambridge Crystallographic Data Centre and Fachinformationszentrum Karlsruhe Access Structures service.

**Supporting Information available**: NMR data of the isolated complexes, x‐ray crystallographic details of **3 a** and **4 a** and further data for the photocatalytic TH of carbonyl compounds promoted by the ruthenium derivatives.

## Conflict of interest

The authors declare no conflict of interest.

1

## Supporting information

As a service to our authors and readers, this journal provides supporting information supplied by the authors. Such materials are peer reviewed and may be re‐organized for online delivery, but are not copy‐edited or typeset. Technical support issues arising from supporting information (other than missing files) should be addressed to the authors.

Supporting Information

## Data Availability

The data that support the findings of this study are available from the corresponding author upon reasonable request.
