# Supplementary material for: Terpyridine Diphosphine Ruthenium Complexes as Efficient Photocatalysts for the Transfer Hydrogenation of Carbonyl Compounds
Source: Chemistry. 2022 Sep 27;28(65):e202201722. doi: 10.1002/chem.202201722 (PMC9828271; doi:10.1002/chem.202201722)
Supplement: Supplementary file 1 — Supporting Information [file CHEM-28-0-s001.pdf]

Chemistry–A European Journal

Supporting Information

Terpyridine Diphosphine Ruthenium Complexes as Efficient Photocatalysts for the Transfer Hydrogenation of Carbonyl Compounds

Maurizio Ballico,* Dario Alessi, Christian Jandl, Denise Lovison, and Walter Baratta*

Chemistry—A European Journal

Supporting Information

Table of Contents:

Figure S1. $^{31}P\{^1H\}$ NMR spectrum of [RuCl(dppp)(tpy)]Cl (**1**) Pag. S7

Figure S2. ^1H NMR spectrum of [RuCl(dppp)(tpy)]Cl (**1**) Pag. S8

Figure S3. $^{13}C\{^1H\}$ DEPTQ NMR spectrum of [RuCl(dppp)(tpy)]Cl (**1**) Pag. S9

Figure S4. ^1H-^1H COSY 2D NMR spectrum of [RuCl(dppp)(tpy)]Cl (**1**) Pag. S10

Figure S5. ^1H-^{13}C HSQC 2D NMR spectrum of [RuCl(dppp)(tpy)]Cl (**1**)) Pag. S11

Figure S6. ^1H-^{31}P HMBC 2D NMR spectrum of [RuCl(dppp)(tpy)]Cl (**1**) Pag. S12

Figure S7. $^{31}P\{^1H\}$ NMR spectrum of [RuCl(dppp)(tpy)]PF_6 (**1a**) Pag. S13

Figure S8. ^1H NMR spectrum of [RuCl(dppp)(tpy)]PF_6 (**1a**) Pag. S14

Figure S9. $^{31}P\{^1H\}$ NMR spectrum of [RuCl((*R*,*R*)-Skewphos)(tpy)]Cl (**2**) Pag. S15

Figure S10. ^1H NMR spectrum of [RuCl((*R*,*R*)-Skewphos)(tpy)]Cl (**2**) Pag. S16

Figure S11. $^{13}C\{^1H\}$ DEPTQ NMR spectrum of [RuCl((*R*,*R*)-Skewphos)(tpy)]Cl (**2**) Pag. S17

Figure S12. ^1H-^1H COSY 2D NMR spectrum of [RuCl((*R*,*R*)-Skewphos)(tpy)]Cl (**2**) Pag. S18

Figure S13. ^1H-^{13}C HSQC 2D NMR spectrum of [RuCl((*R*,*R*)-Skewphos)(tpy)]Cl (**2**) Pag. S19

Figure S14. ^1H-^{31}P HMBC 2D NMR spectrum of [RuCl((*R*,*R*)-Skewphos)(tpy)]Cl (**2**) Pag. S20

Figure S15. ^1H-^1H NOESY 2D NMR spectrum of [RuCl((*R*,*R*)-Skewphos)(tpy)]Cl (**2**) Pag. S21

Figure S16. $^{31}P\{^1H\}$ NMR spectrum of [RuCl((*R*,*R*)-Skewphos)(tpy)]PF_6 (**2a**) Pag. S22

Figure S17. ^1H NMR spectrum of [RuCl((*R*,*R*)-Skewphos)(tpy)]PF_6 (**2a**) Pag. S23

Figure S18. $^{31}P\{^1H\}$ NMR spectrum of [RuCl((*S*,*R*)-Josiphos)(tpy)]PF_6 (**4a**) in CD_2Cl_2 Pag. S24

Figure S19. ^1H NMR spectrum of [RuCl((*S*,*R*)-Josiphos)(tpy)]PF_6 (**4a**) in CD_2Cl_2 Pag. S25

Figure S20. $^{13}C\{^1H\}$ DEPTQ NMR spectrum of [RuCl((*S*,*R*)-Josiphos)(tpy)]PF_6 (**4a**) in CD_2Cl_2 Pag. S26

Figure S21. ^1H-^1H COSY 2D NMR spectrum of [RuCl((*S*,*R*)-Josiphos)(tpy)]PF_6 (**4a**) in CD_2Cl_2 Pag. S27

Figure S22. ^{1}H-^{13}C HSQC 2D NMR spectrum of [RuCl((*S*,*R*)-Josiphos)(tpy)]PF_6 (**4a**) in CD_2Cl_2 Pag. S28

Figure S23. ^{1}H-^{31}P HMBC 2D NMR spectrum of [RuCl((*S*,*R*)-Josiphos)(tpy)]PF_6 (**4a**) in CD_2Cl_2 Pag. S29

Figure S24. ^{1}H-^{1}H NOESY 2D NMR spectrum of [RuCl((*S*,*R*)-Josiphos)(tpy)]PF_6 (**4a**) in CD_2Cl_2 Pag. S30

Figure S25. ^{31}P{^{1}H} NMR spectrum of [RuCl((*S*,*R*)-Josiphos)(tpy)]PF_6 (**4a**) in CD_3OD Pag. S31

Figure S26. ^{1}H NMR spectrum of [RuCl((*S*,*R*)-Josiphos)(tpy)]PF_6 (**4a**) in CD_3OD Pag. S32

Figure S27. ^{13}C{^{1}H} DEPTQ NMR spectrum of [RuCl((*S*,*R*)-Josiphos)(tpy)]PF_6 (**4a**) in CD_3OD Pag. S33

Figure S28. ^{1}H-^{13}C HSQC 2D NMR spectrum of [RuCl((*S*,*R*)-Josiphos)(tpy)]PF_6 (**4a**) in CD_3OD Pag. S34

Figure S29. ^{31}P{^{1}H} NMR spectrum of [RuCl((*R*)-BINAP)(tpy)]Cl (**5**) in CD_2Cl_2 Pag. S35

Figure S30. ^{1}H NMR spectrum of [RuCl((*R*)-BINAP)(tpy)]Cl (**5**) in CD_2Cl_2 Pag. S36

Figure S31. ^{31}P{^{1}H} NMR spectrum of [RuCl((*R*)-BINAP)(tpy)]Cl (**5**) in CD_3OD Pag. S37

Figure S32. ^{31}P{^{1}H} NMR spectrum of [RuCl((*R*)-BINAP)(tpy)]PF_6 (**5a**) Pag. S38

Figure S33. ^{1}H NMR spectrum of [RuCl((*R*)-BINAP)(tpy)]PF_6 (**5a**) Pag. S39

Figure S34. ^{13}C{^{1}H} DEPTQ NMR spectrum of [RuCl((*R*)-BINAP)(tpy)]PF_6 (**5a**) Pag. S40

Figure S35. ^{1}H-^{1}H COSY 2D NMR spectrum of [RuCl((*R*)-BINAP)(tpy)]PF_6 (**5a**) Pag. S41

Figure S36. ^{1}H-^{13}C HSQC 2D NMR spectrum of [RuCl((*R*)-BINAP)(tpy)]PF_6 (**5a**) Pag. S42

Figure S37. ^{1}H-^{31}P HMBC 2D NMR spectrum of [RuCl((*R*)-BINAP)(tpy)]PF_6 (**5a**) Pag. S43

Figure S38. ^{1}H-^{1}H NOESY 2D NMR spectrum of [RuCl((*R*)-BINAP)(tpy)]PF_6 (**5a**) Pag. S44

Figure S39. Photographs of the color evolution of the reaction mixtures during the photocatalytic TH of carbonyl compounds Pag. S45

Table S1. Further data regarding the Photocatalytic TH of ketones and aldehydes with complexes **1**, **2**, and **3a** at 30 °C Pag. S46

Figure S40. GC-FID chromatogram of the reaction mixture of the enantioselective photocatalytic TH of acetophenone promoted by complex **3** at S/C 1000 Pag. S47

Figure S41. Comparison between the GC-FID chromatograms of the enantioselective photocatalytic TH of acetophenone promoted by complex **2** (A) and **3** (B) at S/C 1000 Pag. S48

Figure S42. GC-FID chromatogram (A) of the reaction mixture of the enantioselective photocatalytic TH of 4'-methyl-acetophenone promoted by complex **3** at S/C 1000, and detailed of the peaks of (*R*)- and (*S*)-1-(tolyl)ethanol (B). Pag. S49

Figure S43. $^{31}P\{^1H\}$ NMR spectrum of [RuCl((*R*,*R*)- Skewphos)(tpy)]Cl (**2**) in 2-propanol-d^8 Pag. S50

Figure S44. ^1H NMR spectrum of [RuCl((*R*,*R*)- Skewphos)(tpy)]Cl (**2**) in 2-propanol-d^8 Pag. S51

Figure S45. $^{31}P\{^1H\}$ NMR spectrum of the mixture obtained from [RuCl((*R*,*R*)- Skewphos)(tpy)]Cl (**2**) by addition of NaO*i*Pr (3 equiv) in 2-propanol-d^8 in the dark Pag. S52

Figure S46. $^{31}P\{^1H\}$ NMR spectrum of [Ru(O*i*Pr)((*R*,*R*)- Skewphos)(tpy)](O*i*Pr) (**A**) in 2-propanol-d^8 Pag. S53

Figure S47. ^1H NMR spectrum of [Ru(O*i*Pr)((*R*,*R*)- Skewphos)(tpy)](O*i*Pr) (**A**) in 2-propanol-d^8 Pag. S54

Figure S48. $^{13}C\{^1H\}$ DEPTQ NMR spectrum of [Ru(O*i*Pr)((*R*,*R*)- Skewphos)(tpy)](O*i*Pr) (**A**) in 2-propanol-d^8 Pag. S55

Figure S49. $^{13}C\{^1H\}$ DEPT135 NMR spectrum of [Ru(O*i*Pr)((*R*,*R*)- Skewphos)(tpy)](O*i*Pr) (**A**) in 2-propanol-d^8 Pag. S56

Figure S50. ^1H-^{13}C HSQC 2D NMR spectrum of [Ru(O*i*Pr)((*R*,*R*)- Skewphos)(tpy)](O*i*Pr) (**A**) in 2-propanol-d^8 Pag. S57

Figure S51. Aliphatic region of the ^1H-^{13}C HSQC 2D NMR spectrum of [Ru(O*i*Pr)((*R*,*R*)-Skewphos)(tpy)](O*i*Pr) (**A**) in 2-propanol-d^8 Pag. S58

Figure S52. ^1H-^{31}P HMBC 2D NMR spectrum of [Ru(O*i*Pr)((*R*,*R*)- Skewphos)(tpy)](O*i*Pr) (**A**) in 2-propanol-d^8 Pag. S59

Figure S53. $^{31}P\{^1H\}$ NMR spectrum with evidences of the formation of [RuH((*R*,*R*)-Skewphos)(tpy)](O*i*Pr) (**B**) in 2-propanol-d^8 Pag. S60

Figure S54. ^1H NMR spectrum with evidences of the formation of [RuH((*R*,*R*)-Skewphos)(tpy)](O*i*Pr) (**B**) in 2-propanol-d^8 Pag. S61

Figure S55. $^{31}P\{^1H\}$ NMR spectrum with evidences of the formation of [RuH((*R*,*R*)-Skewphos)(tpy)](O*i*Pr) (**B**) in 2-propanol/toluene-d^8 1:1 (v/v) Pag. S62

Figure S56. ^1H NMR spectrum with evidences of the formation of [RuH((*R*,*R*)-Skewphos)(tpy)](O*i*Pr) (**B**) in 2-propanol/toluene-d^8 1:1 (v/v) Pag. S63

Figure S57. ^1H NMR spectrum with evidences of the reduction of benzophenone to benzhydrol promoted by the mixture containing [RuH((*R*,*R*)- Skewphos)(tpy)](O*i*Pr) (**B**) in 2-propanol/toluene-d^8 1:1 (v/v) Pag. S64

Figure S58. $^{31}P\{^1H\}$ NMR spectrum with evidences of the reduction of benzophenone to benzhydrol promoted by the mixture containing [RuH((*R*,*R*)- Skewphos)(tpy)](O*i*Pr) (**B**) in 2-propanol/toluene-d^8 1:1 (v/v) Pag. S65

Figure S59. ^1H NMR spectrum with evidences of the reduction of benzophenone to benzhydrol promoted by the mixture containing [RuH((*R*,*R*)- Skewphos)(tpy)](O*i*Pr) (**B**) in 2-propanol-d^8, obtained after visible light irradiation Pag. S66

Figure S60. $^{31}P\{^1H\}$ NMR spectrum obtained after the addition of benzophenone to the mixture containing [RuH((*R*,*R*)- Skewphos)(tpy)](O*i*Pr) (**B**) in 2-propanol-d^8 and visible light irradiation Pag. S67

Figure S61. $^{13}C\{^1H\}$ DEPTQ NMR spectrum with evidences of the reduction of benzophenone to benzhydrol promoted by the mixture containing [RuH((*R*,*R*)- Skewphos)(tpy)](O*i*Pr) (**B**) in 2-propanol-d^8, obtained after visible light irradiation Pag. S68

Figure S62. Aromatic region of the ^1H-^{13}C HSQC 2D NMR spectrum with evidences of the reduction of benzophenone to benzhydrol promoted by the mixture containing [RuH((*R*,*R*)-

Skewphos)(tpy)](O*i*Pr) (**B**) in 2-propanol-*d*^{8}, obtained after visible light irradiation Pag. S69

Figure S63. Effect of the addition of benzophenone (1-2 equiv) to the mixture containing [RuH((*R*,*R*)- Skewphos)(tpy)](O*i*Pr) (**B**) and after visible light irradiation in the ^{31}P{^{1}H} NMR spectrum (162.0 MHz) in 2-propanol-*d*^{8} Pag. S70

Figure S64. Effect of the addition of benzophenone (1-2 equiv) to the mixture containing [RuH((*R*,*R*)- Skewphos)(tpy)](O*i*Pr) (**B**) and after visible light irradiation in the aromatic region of the ^{1}H NMR spectrum (400.1 MHz) in 2-propanol-*d*^{8} Pag. S71

Single crystal X-ray structure determination of Compounds 3a and 4a. General data. Pag. S72

Figure S65. Molecular structure of complex **3a** Pag. S73

Single crystal X-ray structure determination of complex 3a. Detailed crystallographic data. Pag. S74

Figure S66. Molecular structure of complex **4a** Pag. S76

Single crystal X-ray structure determination of complex 4a. Detailed crystallographic data. Pag. S77

References Pag. S79

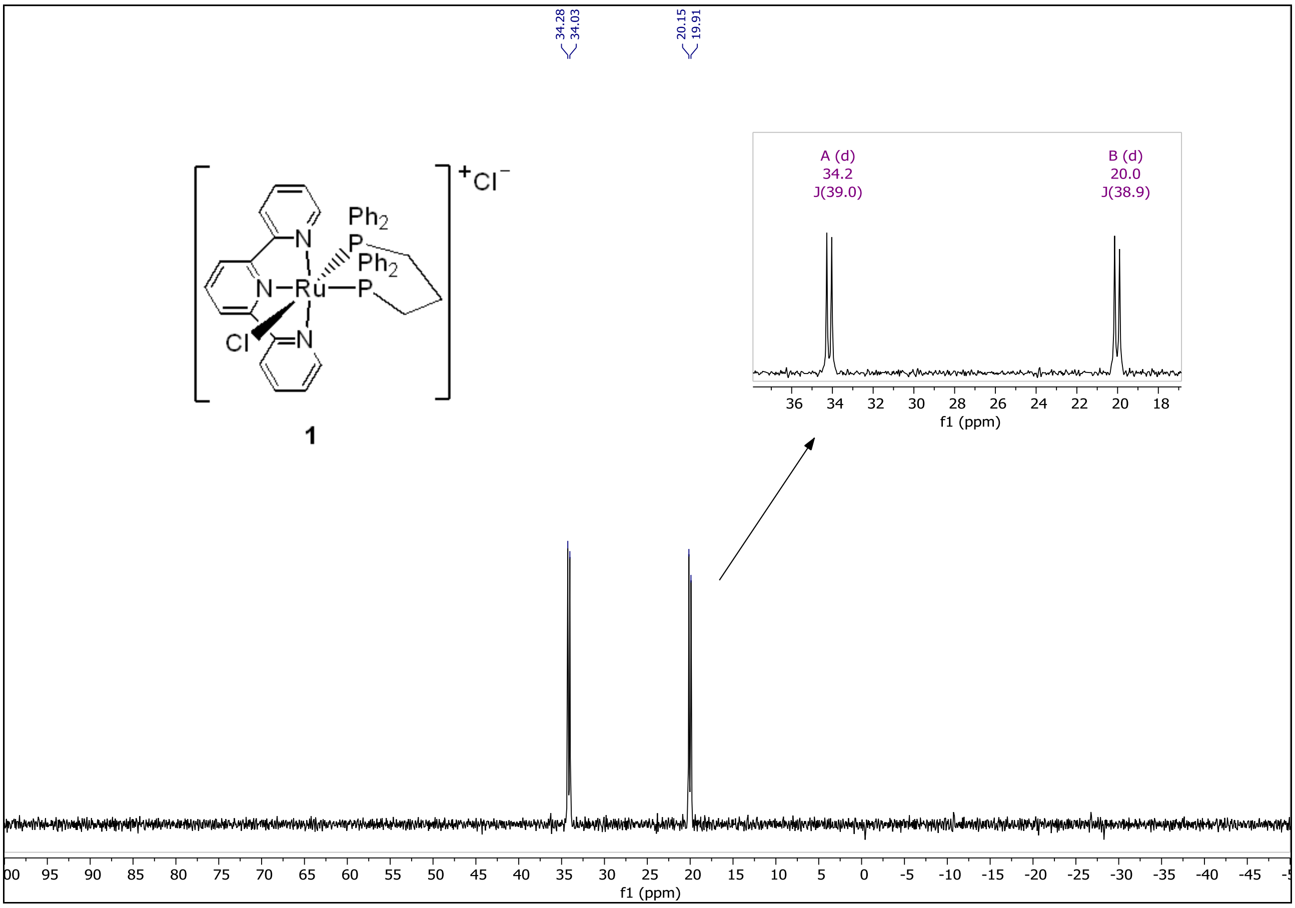

Figure S1. $^{31}P\{^{1}H\}$ NMR spectrum (162.0 MHz) of [RuCl(dppp)(tpy)]Cl (**1**) in CD_2Cl_2 at 25 °C.

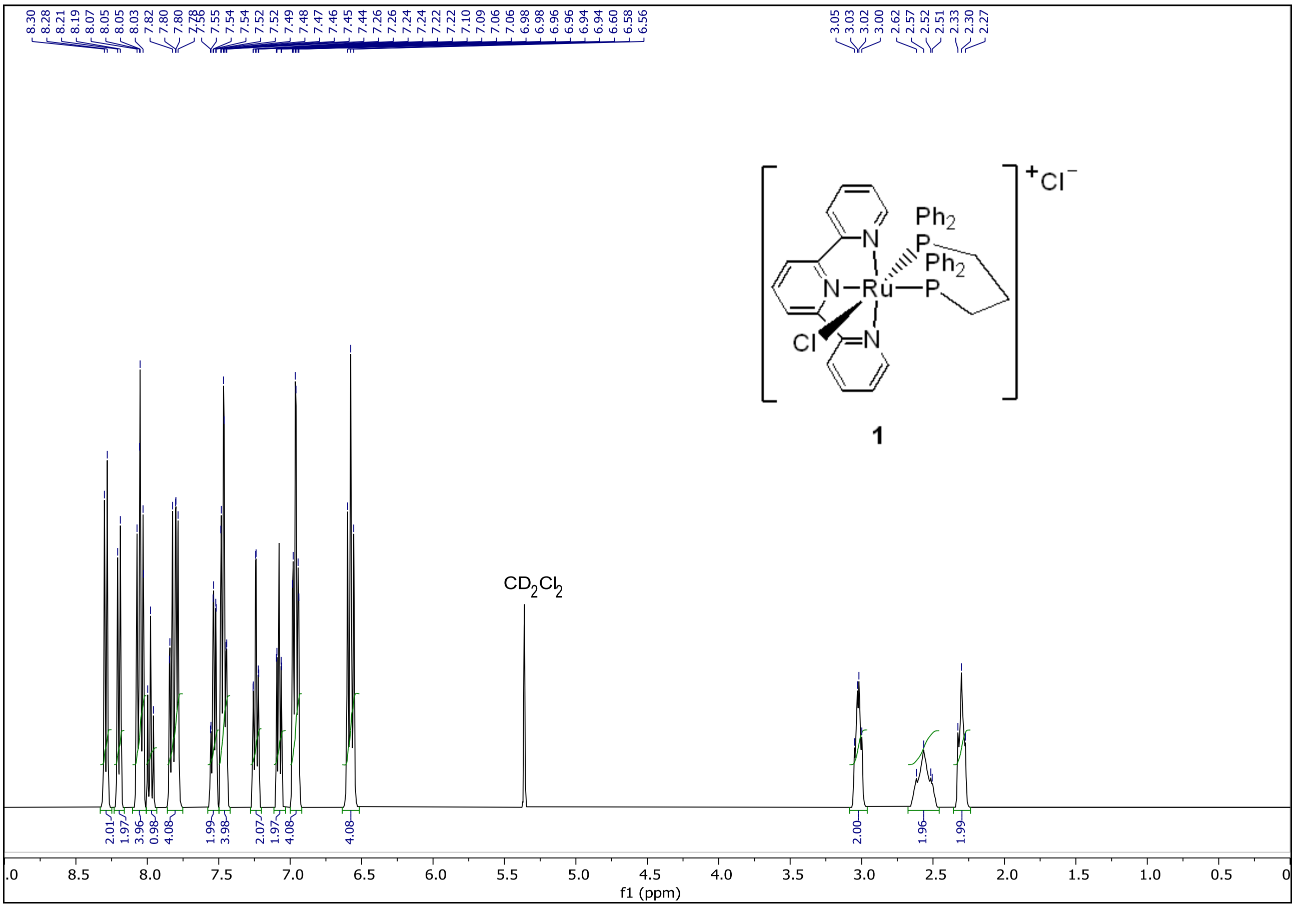

Figure S2. ^{1}H NMR spectrum (400.1 MHz) of [RuCl(dppp)(tpy)]Cl (**1**) in CD_2Cl_2 at 25 °C.

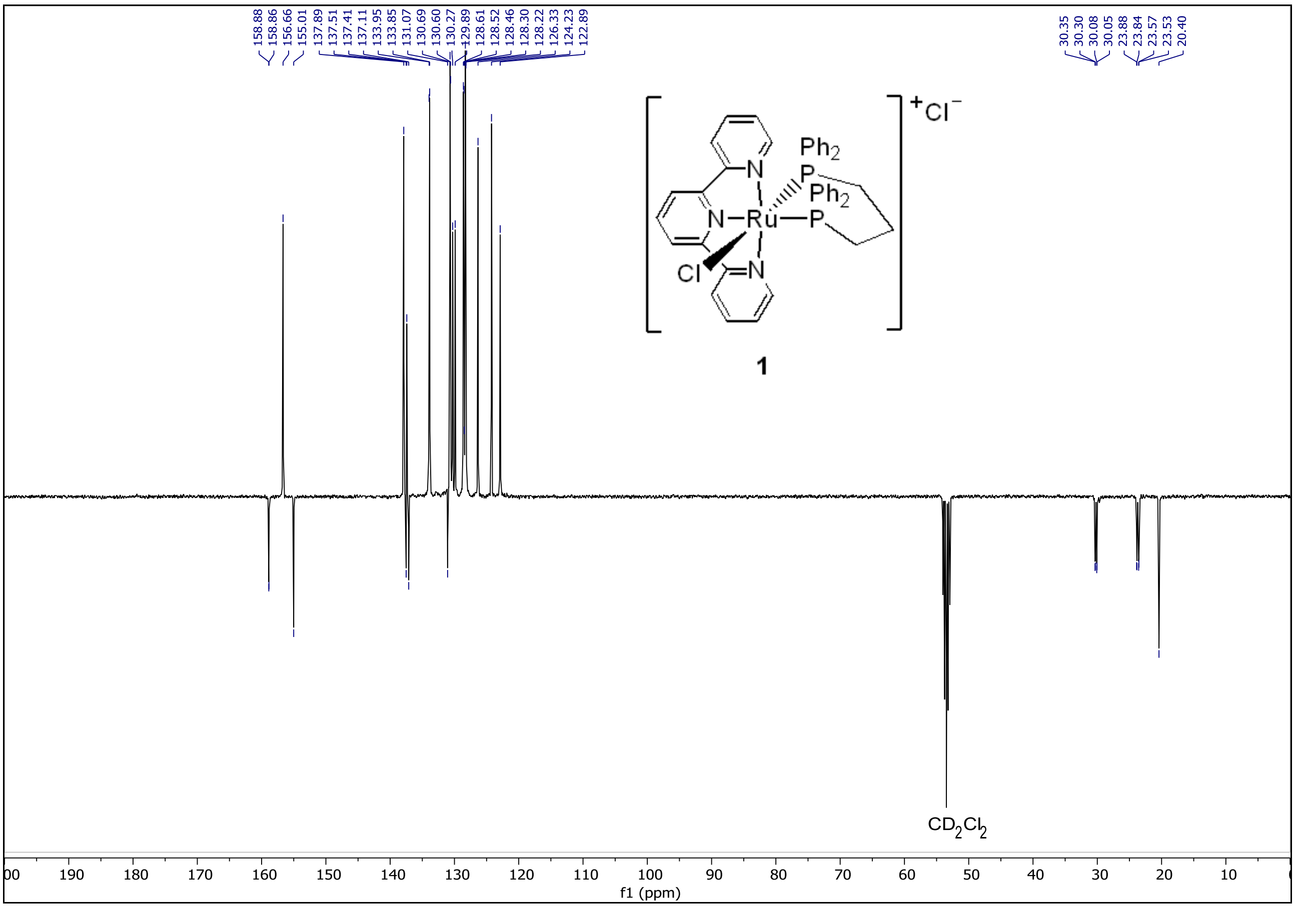

Figure S3. $^{13}C\{^1H\}$ DEPTQ NMR spectrum (100.6 MHz) of [RuCl(dppp)(tpy)]Cl (**1**) in CD_2Cl_2 at 25 °C.

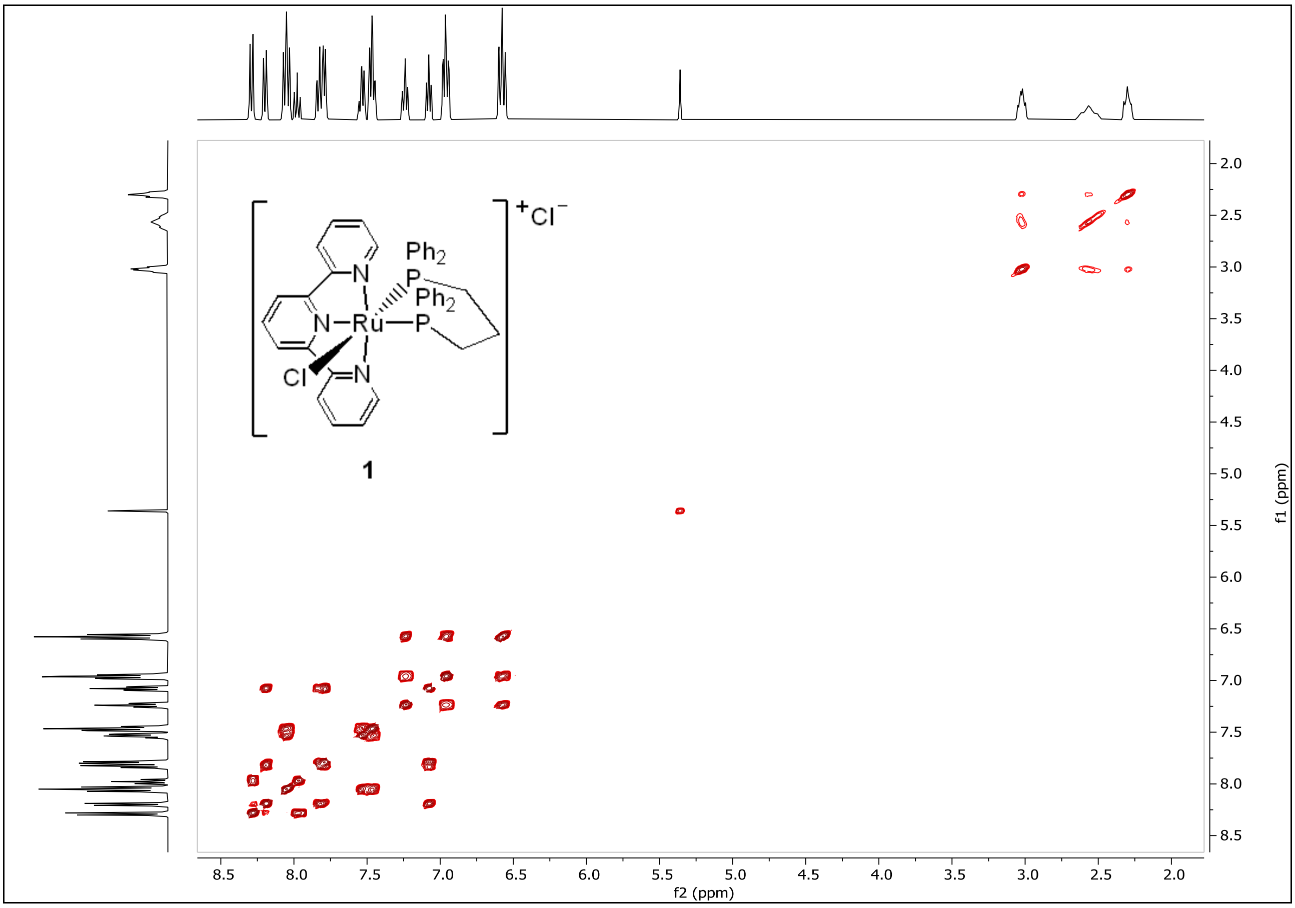

Figure S4. ^{1}H-^{1}H COSY 2D NMR spectrum of [RuCl(dppp)(tpy)]Cl (**1**) in CD_2Cl_2 at 25 °C.

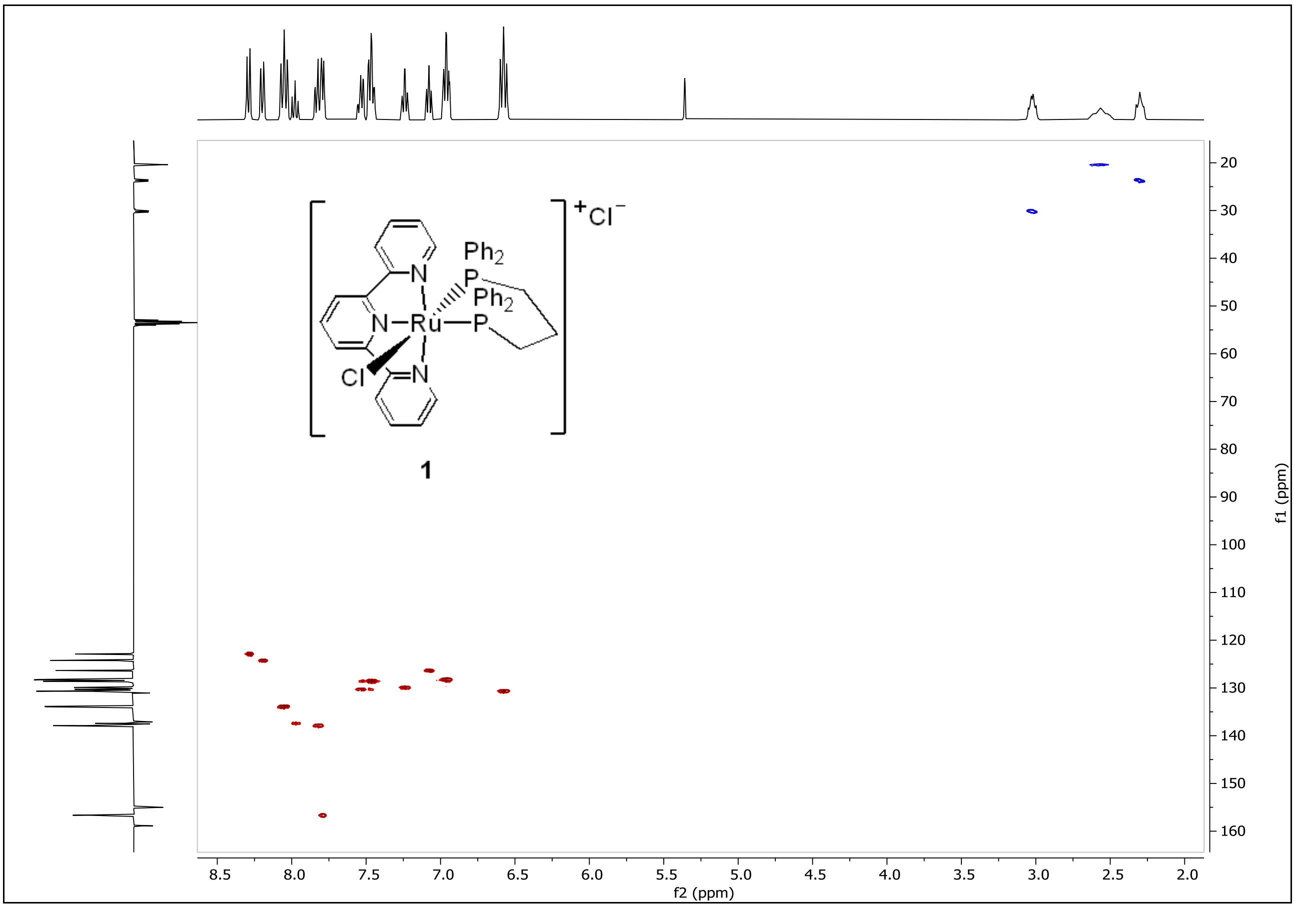

Figure S5. ^{1}H-^{13}C HSQC 2D NMR spectrum of [RuCl(dppp)(tpy)]Cl (**1**) in CD_2Cl_2 at 25 °C.

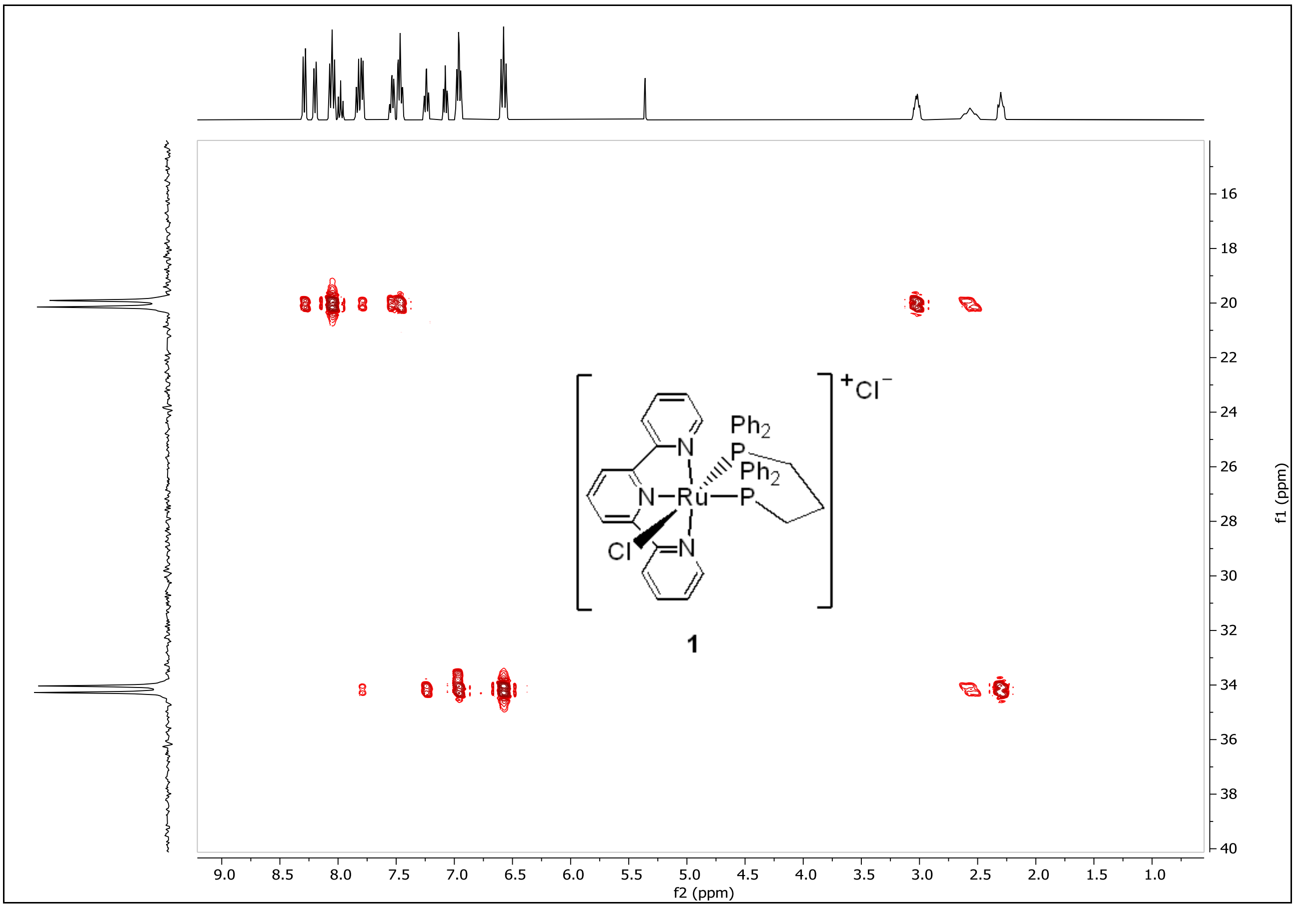

Figure S6. ^{1}H-^{31}P HMBC 2D NMR spectrum of [RuCl(dppp)(tpy)]Cl (**1**) in CD_2Cl_2 at 25 °C.

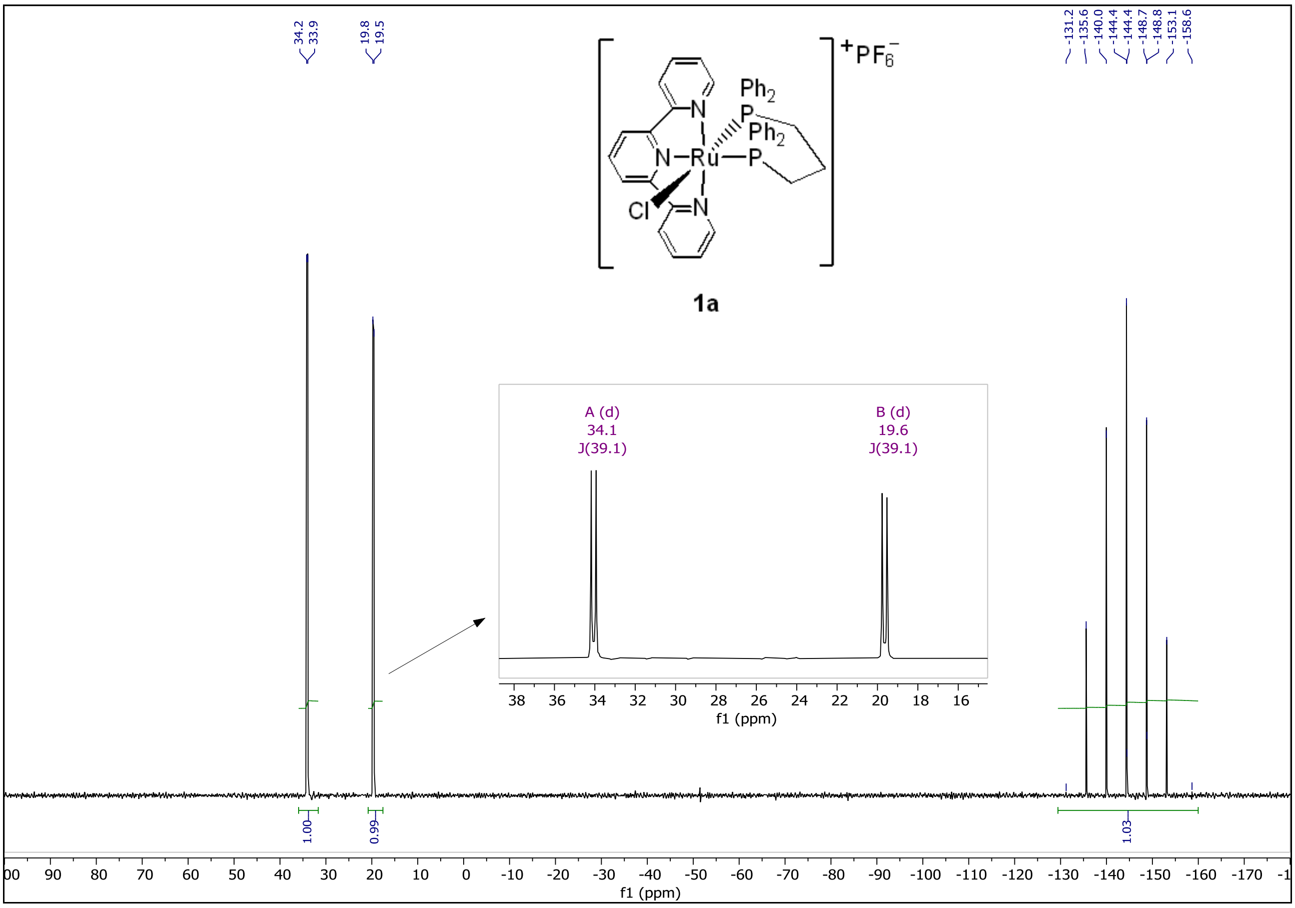

Figure S7. $^{31}P\{^{1}H\}$ NMR spectrum (162.0 MHz) of $[RuCl(dppp)(tpy)]PF_6$ (**1a**) in CD_2Cl_2 at 25 °C.

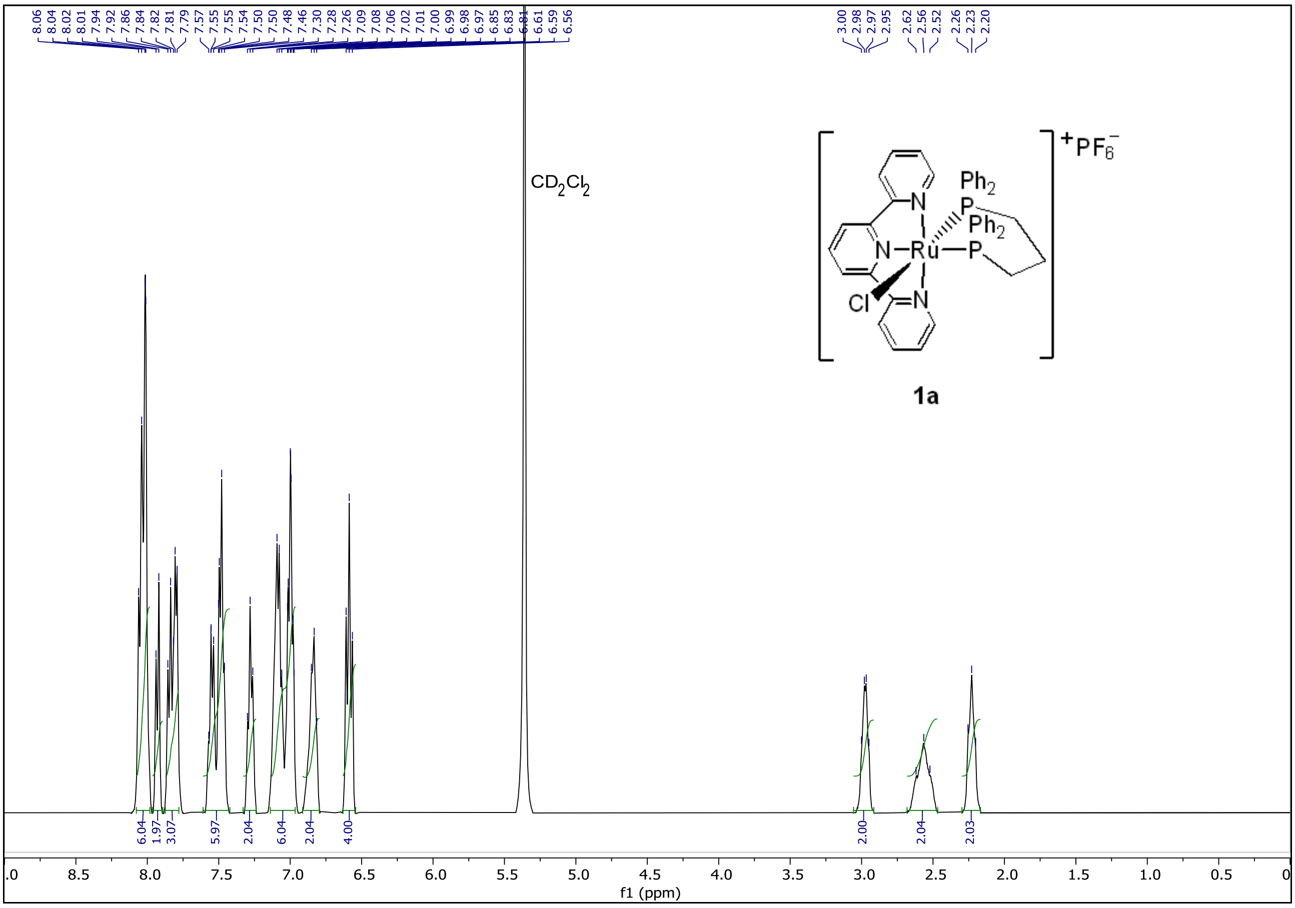

Figure S8. ^{1}H NMR spectrum (400.1 MHz) of [RuCl(dppp)(tpy)]PF$_6$ (**1a**) in CD$_2$Cl$_2$ at 25 °C.

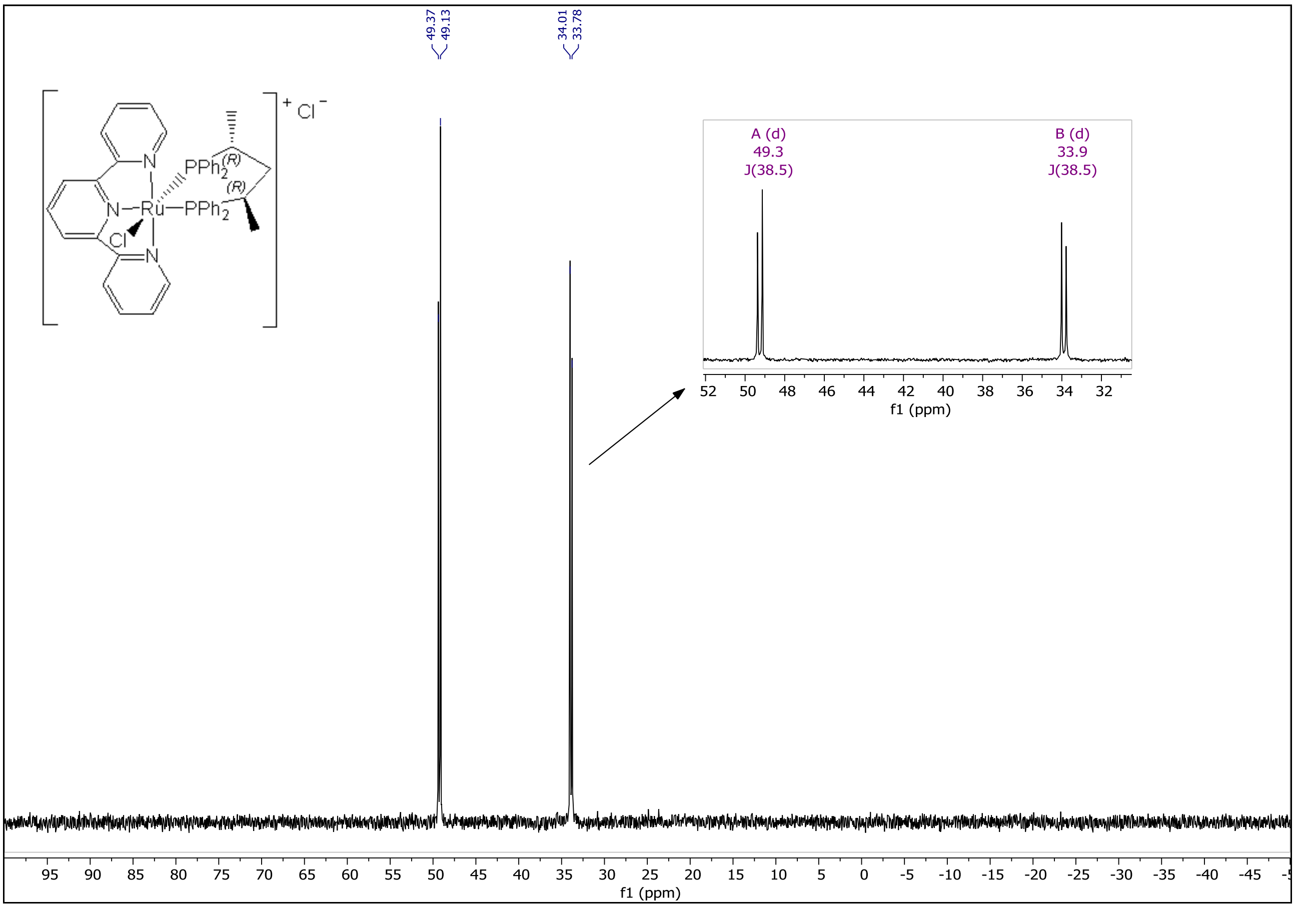

Figure S9. $^{31}P\{^{1}H\}$ NMR spectrum (162.0 MHz) of [RuCl((*R*,*R*)-Skewphos)(tpy)]Cl (**2**) in CD_2Cl_2 at 25 °C.

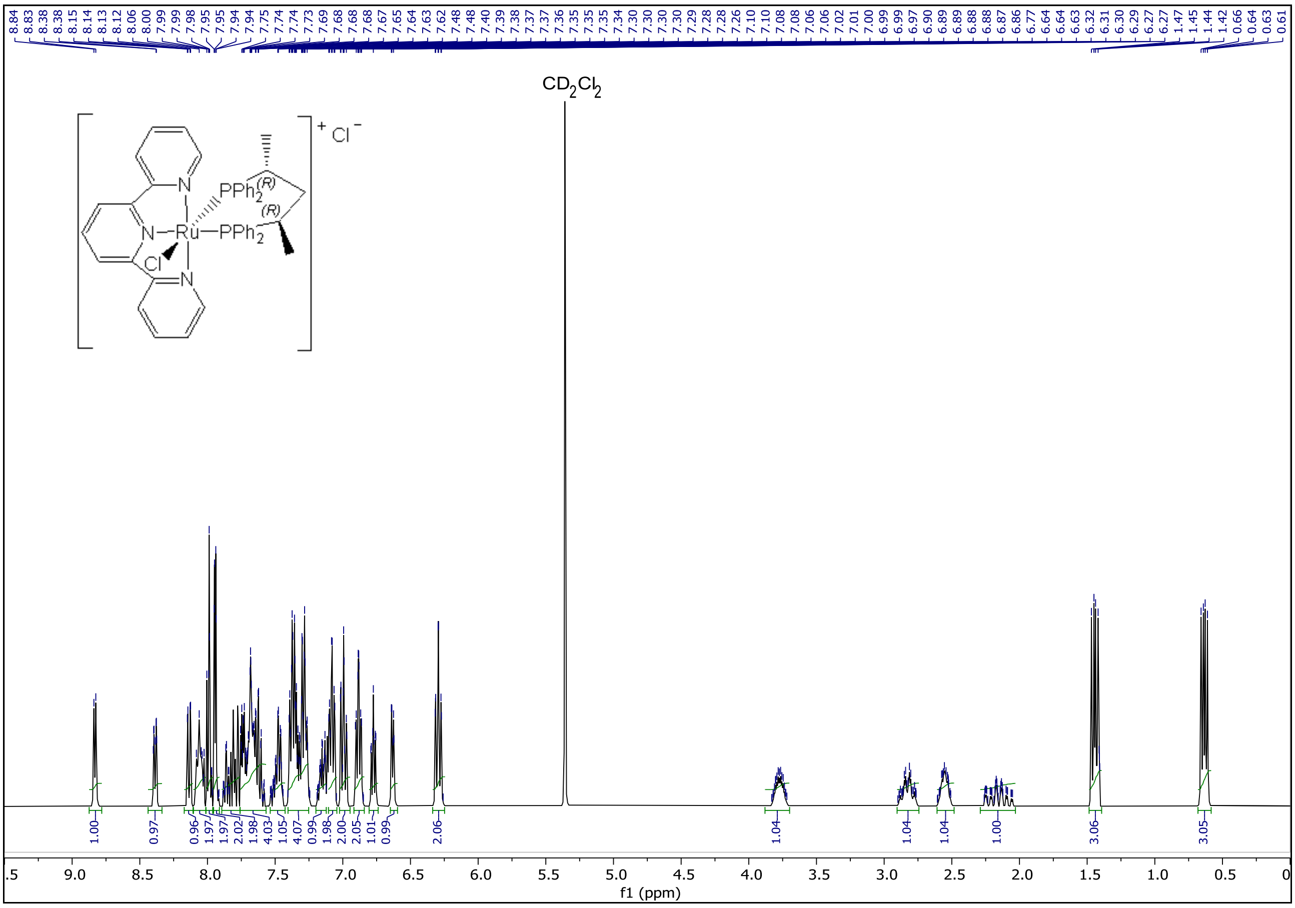

Figure S10. ^{1}H NMR spectrum (400.1 MHz) of [RuCl((*R*,*R*)-Skewphos)(tpy)]Cl (**2**) in CD_2Cl_2 at 25 °C.

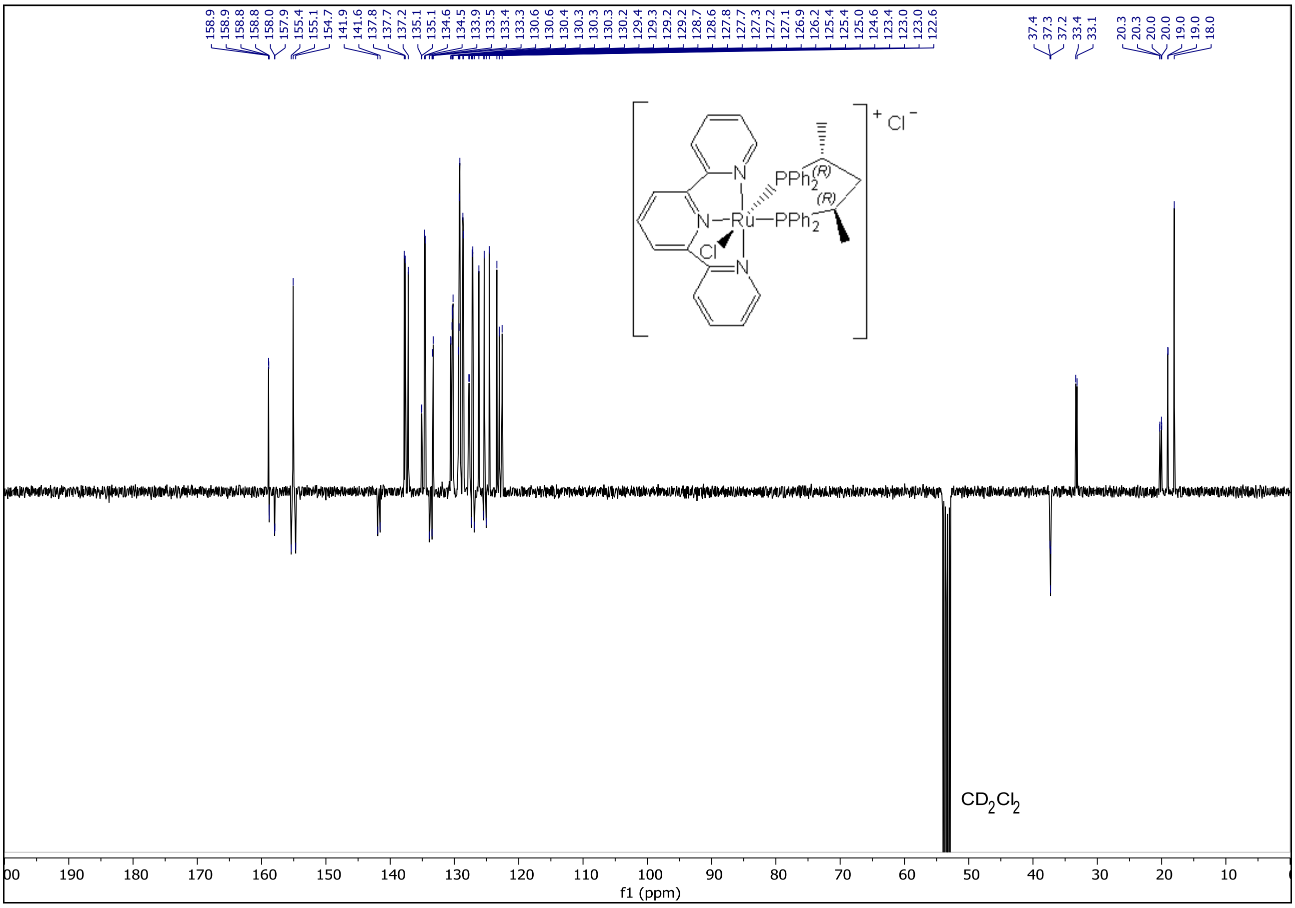

Figure S11. $^{13}C\{^1H\}$ DEPTQ NMR spectrum (100.6 MHz) of [RuCl((*R*,*R*)-Skewphos)(tpy)]Cl (**2**) in CD_2Cl_2 at 25 °C.

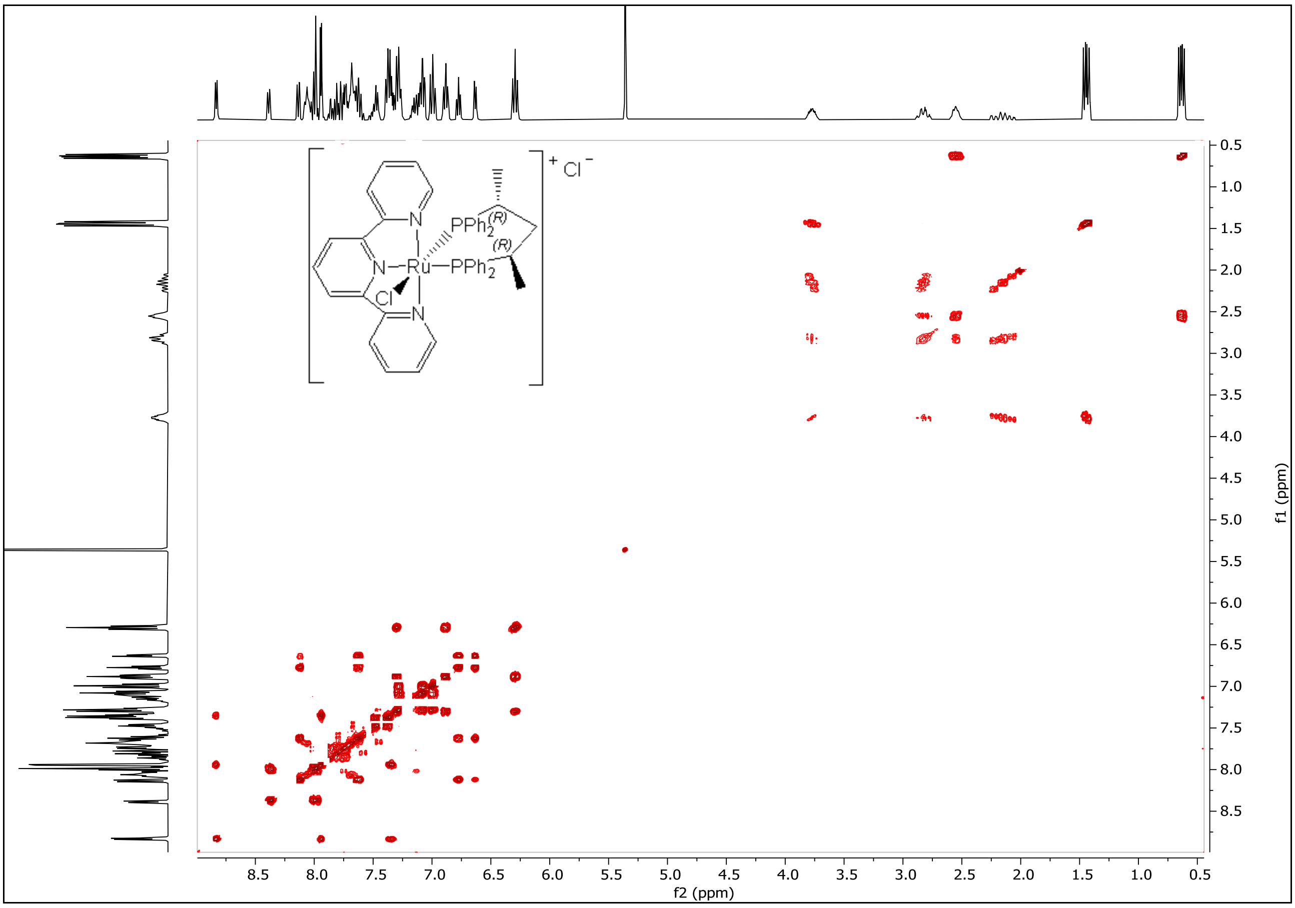

Figure S12. ^{1}H-^{1}H COSY 2D NMR spectrum of [RuCl((*R*,*R*)-Skewphos)(tpy)]Cl (**2**) in CD_2Cl_2 at 25 °C.

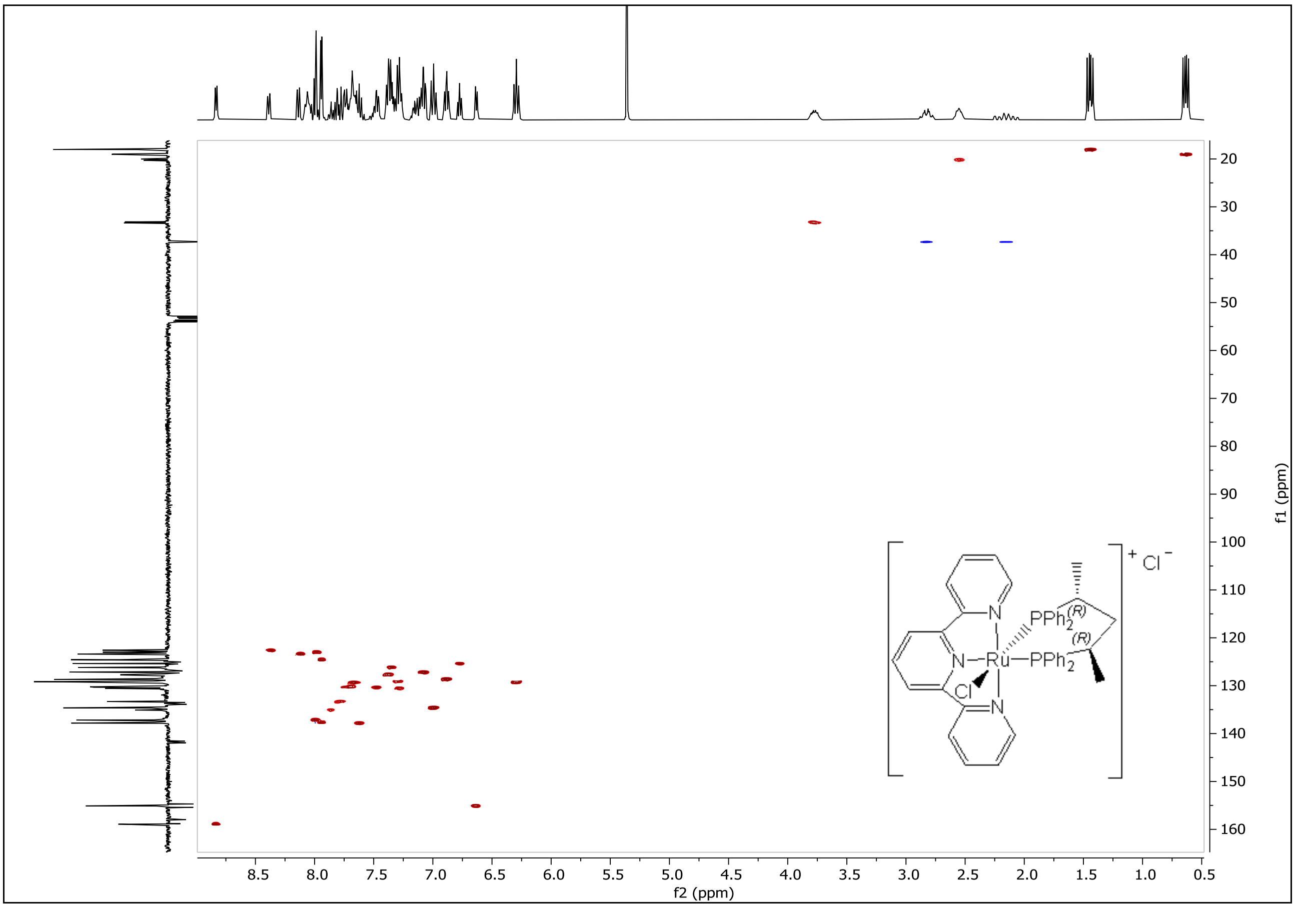

Figure S13. ^{1}H-^{13}C HSQC 2D NMR spectrum of [RuCl((*R*,*R*)-Skewphos)(tpy)]Cl (**2**) in CD_2Cl_2 at 25 °C.

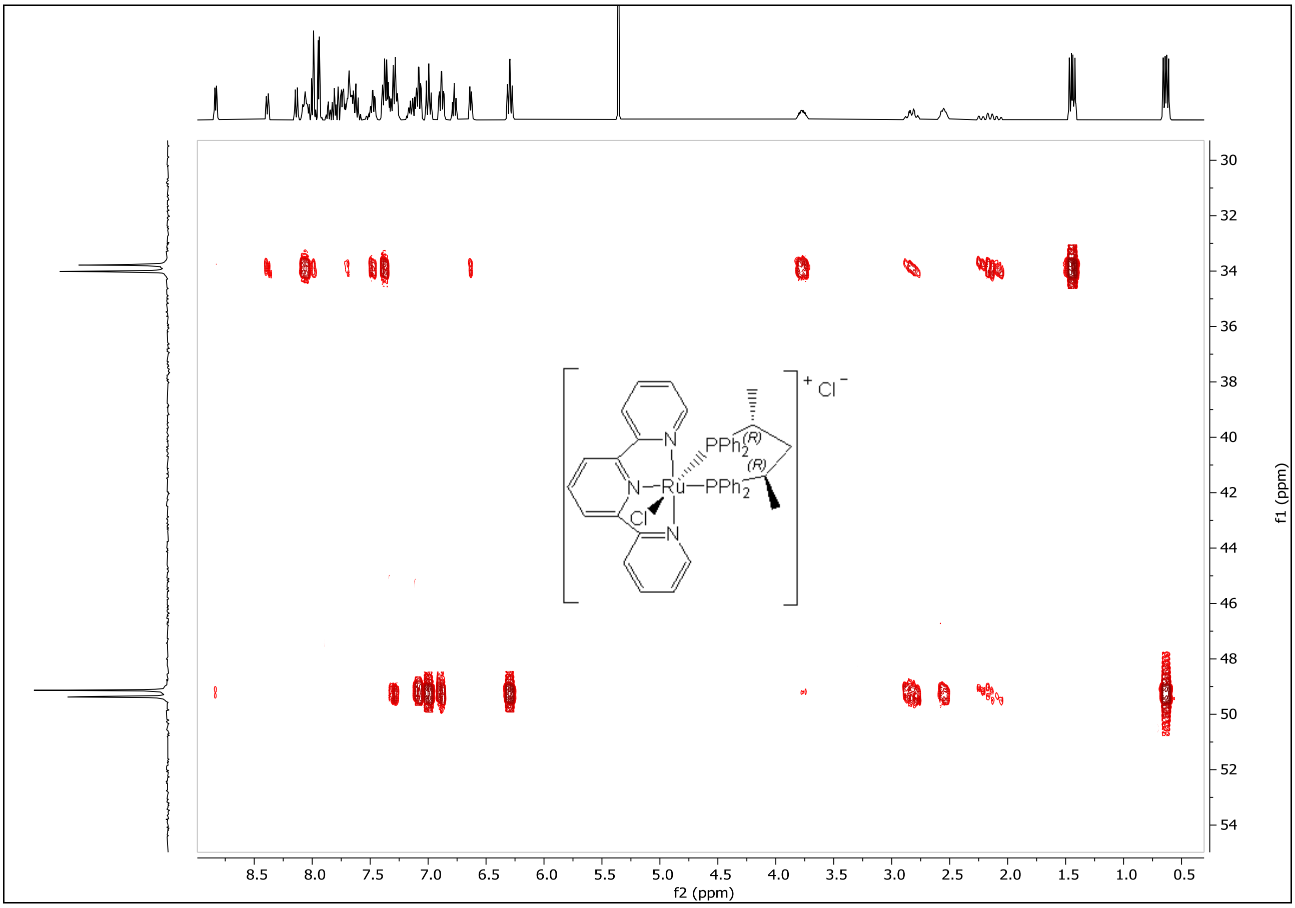

Figure S14. ^{1}H-^{31}P HMBC 2D NMR spectrum of [RuCl((*R*,*R*)-Skewphos)(tpy)]Cl (**2**) in CD_2Cl_2 at 25 °C.

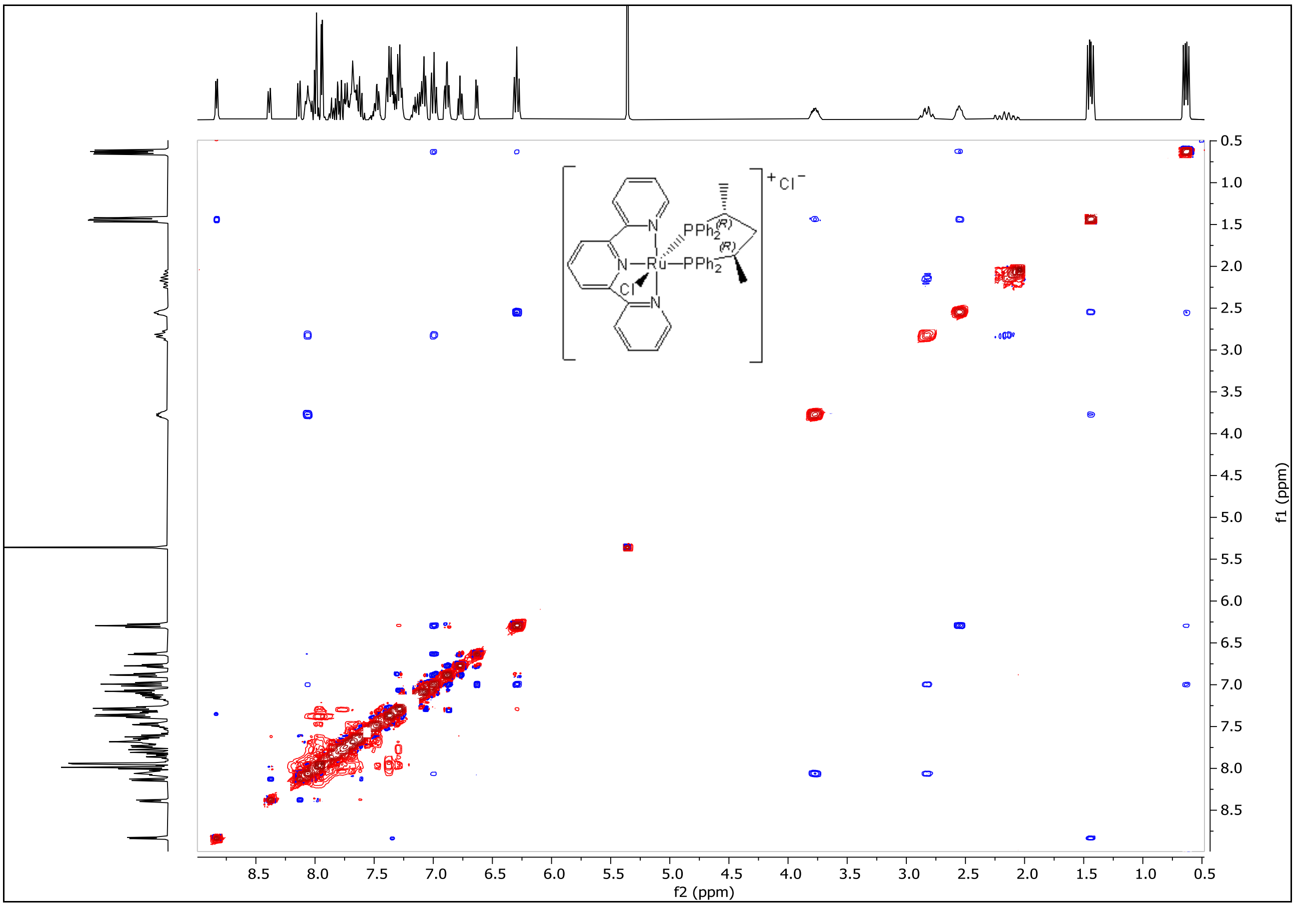

Figure S15. ^{1}H-^{1}H NOESY 2D NMR spectrum of [RuCl((*R*,*R*)-Skewphos)(tpy)]Cl (**2**) in CD_2Cl_2 at 25 °C.

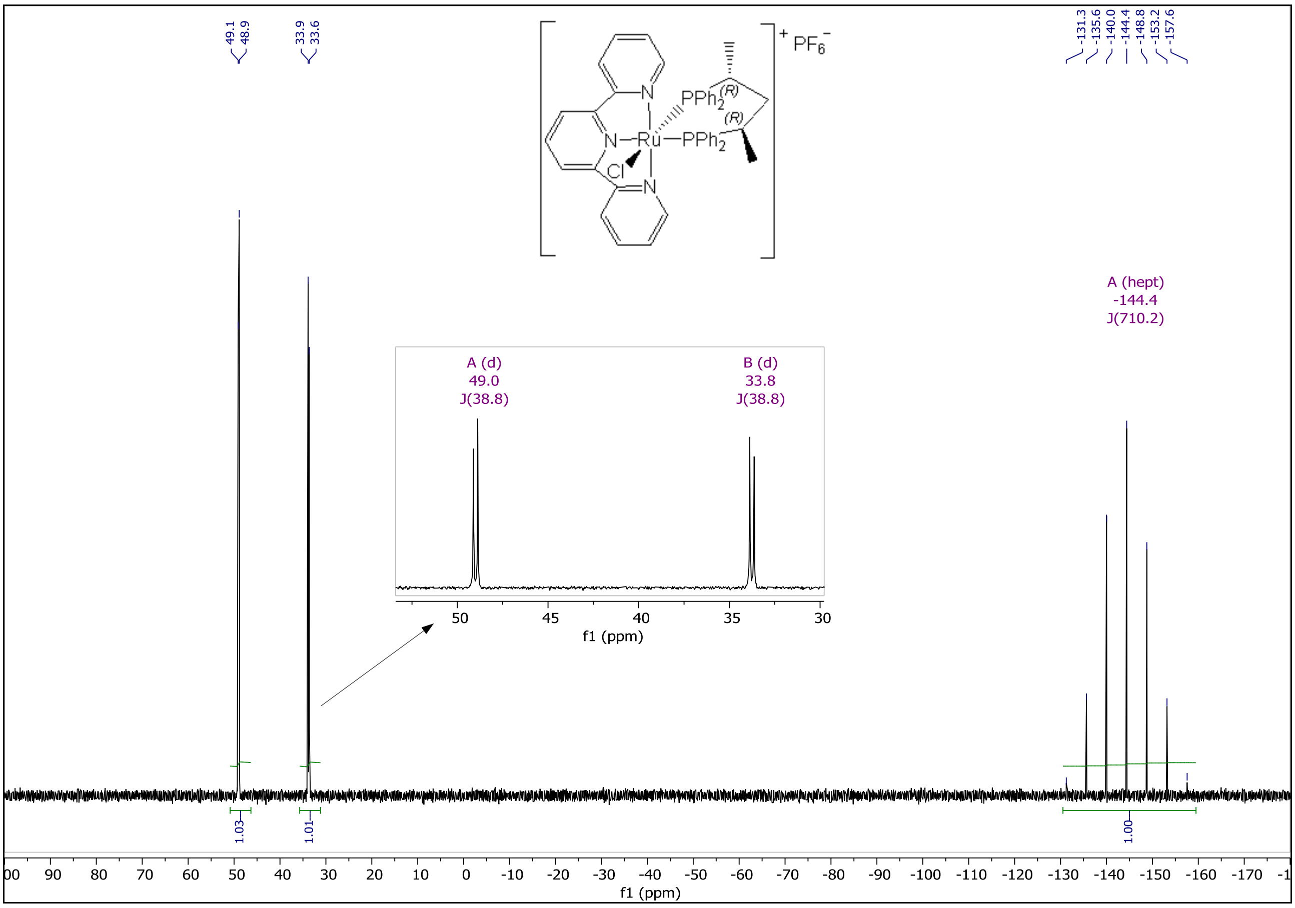

Figure S16. $^{31}P\{^{1}H\}$ NMR spectrum (162.0 MHz) of [RuCl((*R*,*R*)-Skewphos)(tpy)]PF_6 (**2a**) in CD_2Cl_2 at 25 °C.

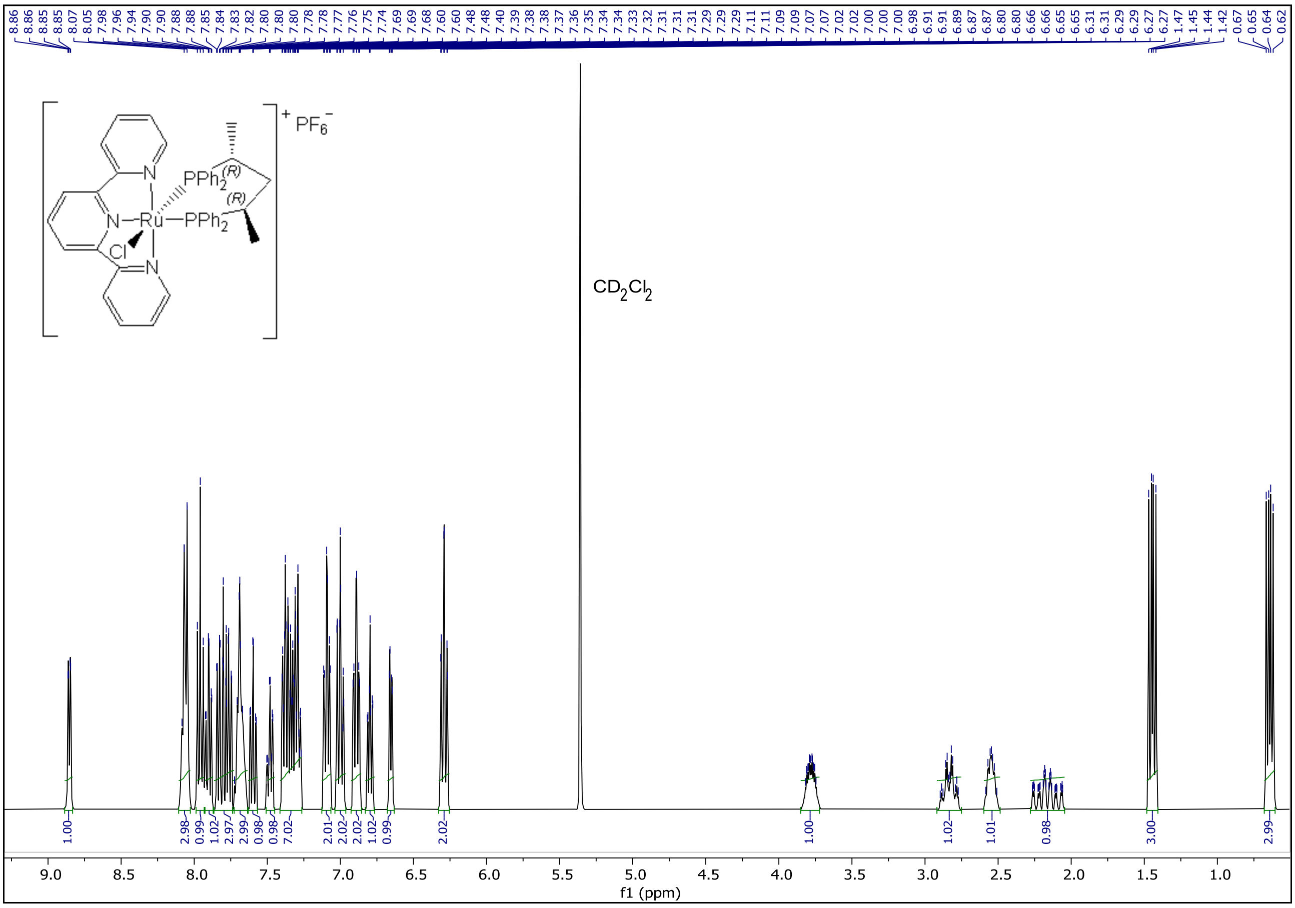

Figure S17. ^{1}H NMR spectrum (400.1 MHz) of [RuCl((*R*,*R*)-Skewphos)(tpy)]PF_6 (**2a**) in CD_2Cl_2 at 25 °C.

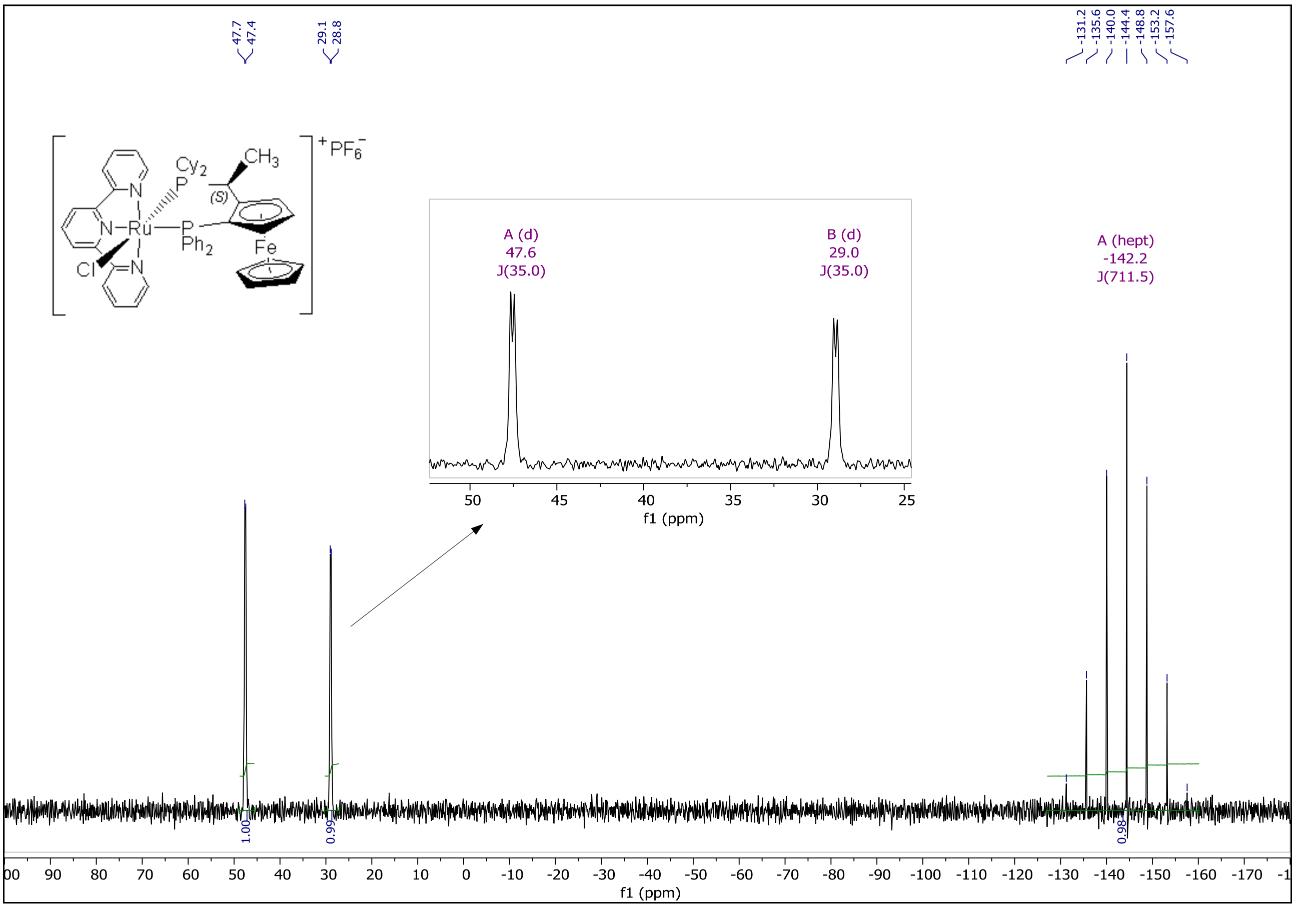

Figure S18. $^{31}P\{^{1}H\}$ NMR spectrum (162.0 MHz) of [RuCl((*S*,*R*)-Josiphos)(tpy)]PF_6 (**4a**) in CD_2Cl_2 at 25 °C.

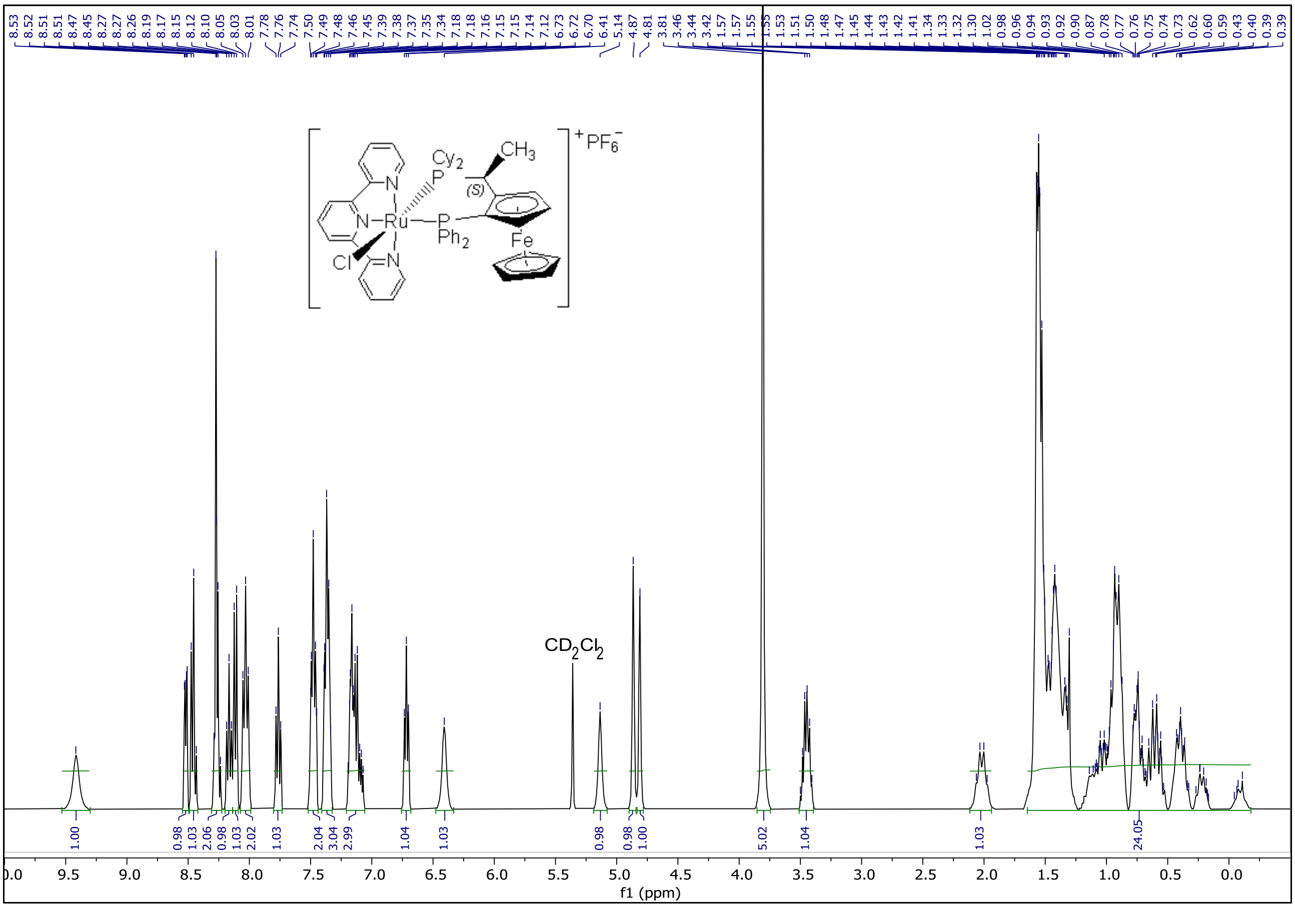

Figure S19. ^{1}H NMR spectrum (400.1 MHz) of [RuCl((*S*,*R*)-Josiphos)(tpy)]PF$_6$ (**4a**) in CD$_2$Cl$_2$ at 25 °C.

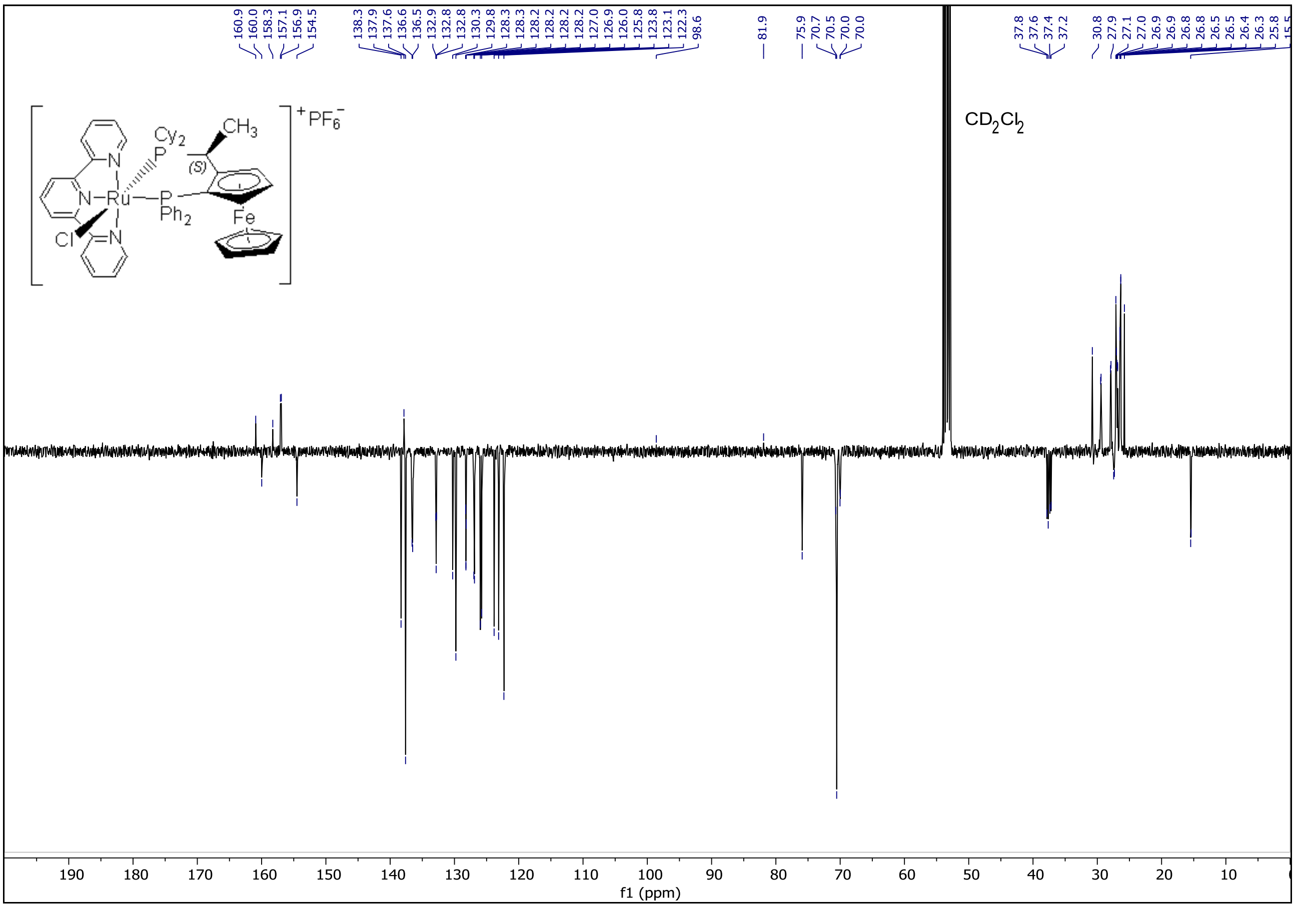

Figure S20. $^{13}C\{^{1}H\}$ DEPTQ NMR spectrum (100.6 MHz) of [RuCl((*S*,*R*)-Josiphos)(tpy)]PF_6 (**4a**) in CD_2Cl_2 at 25 °C.

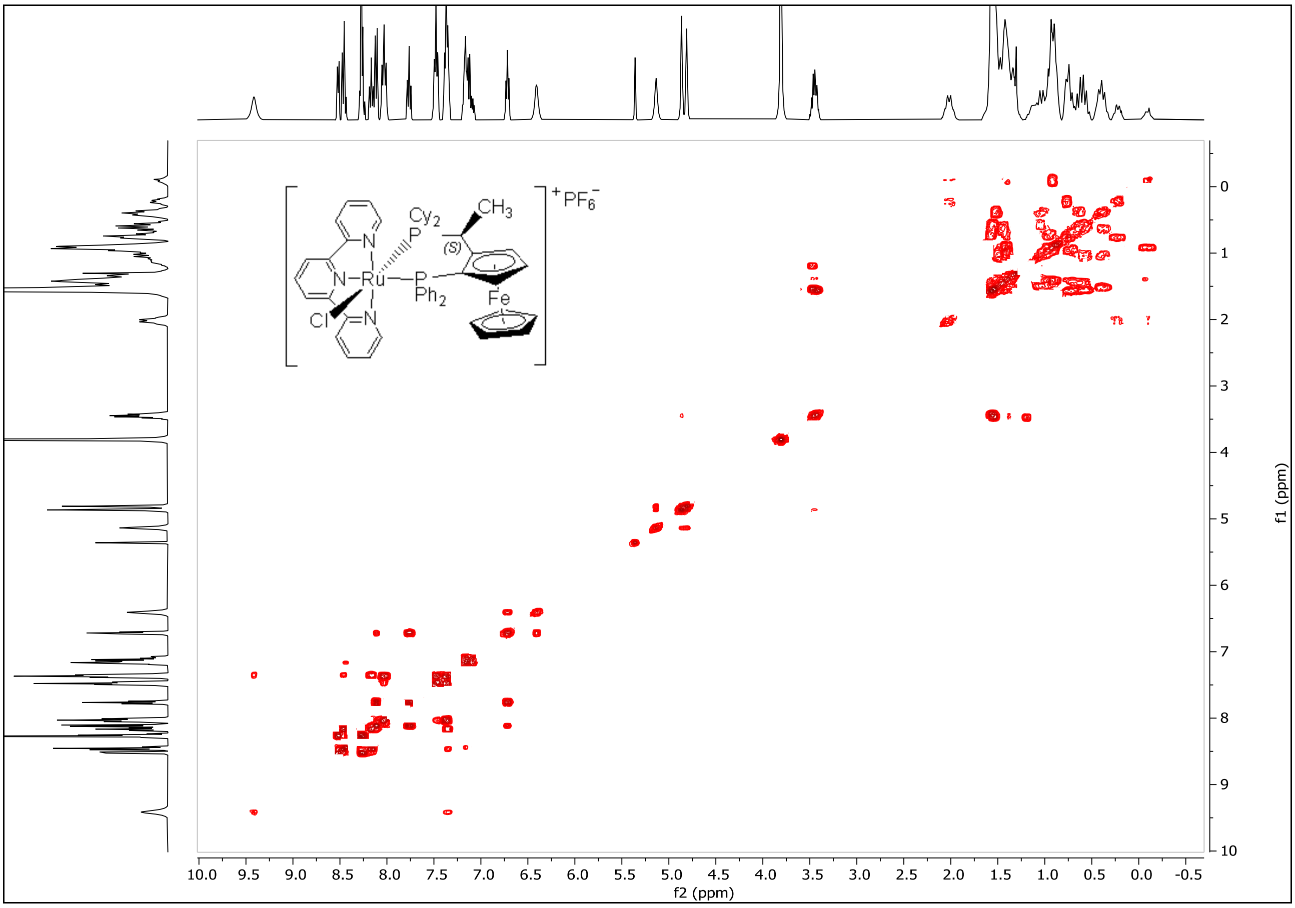

Figure S21. ^{1}H-^{1}H COSY 2D NMR spectrum of [RuCl((*S*,*R*)-Josiphos)(tpy)]PF_6 (**4a**) in CD_2Cl_2 at 25 °C.

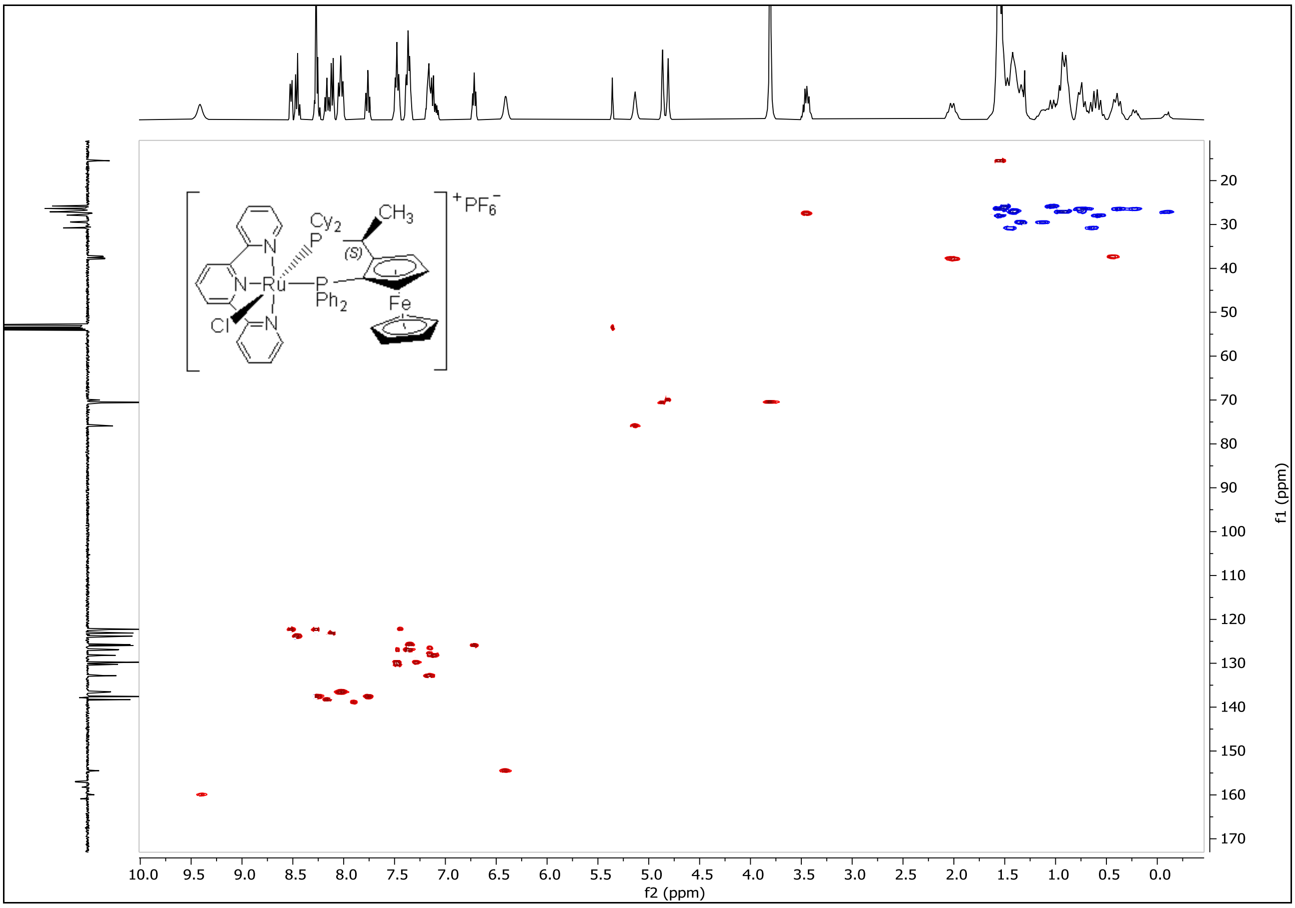

Figure S22. ^{1}H-^{13}C HSQC 2D NMR spectrum of [RuCl((*S*,*R*)-Josiphos)(tpy)]PF_6 (**4a**) in CD_2Cl_2 at 25 °C.

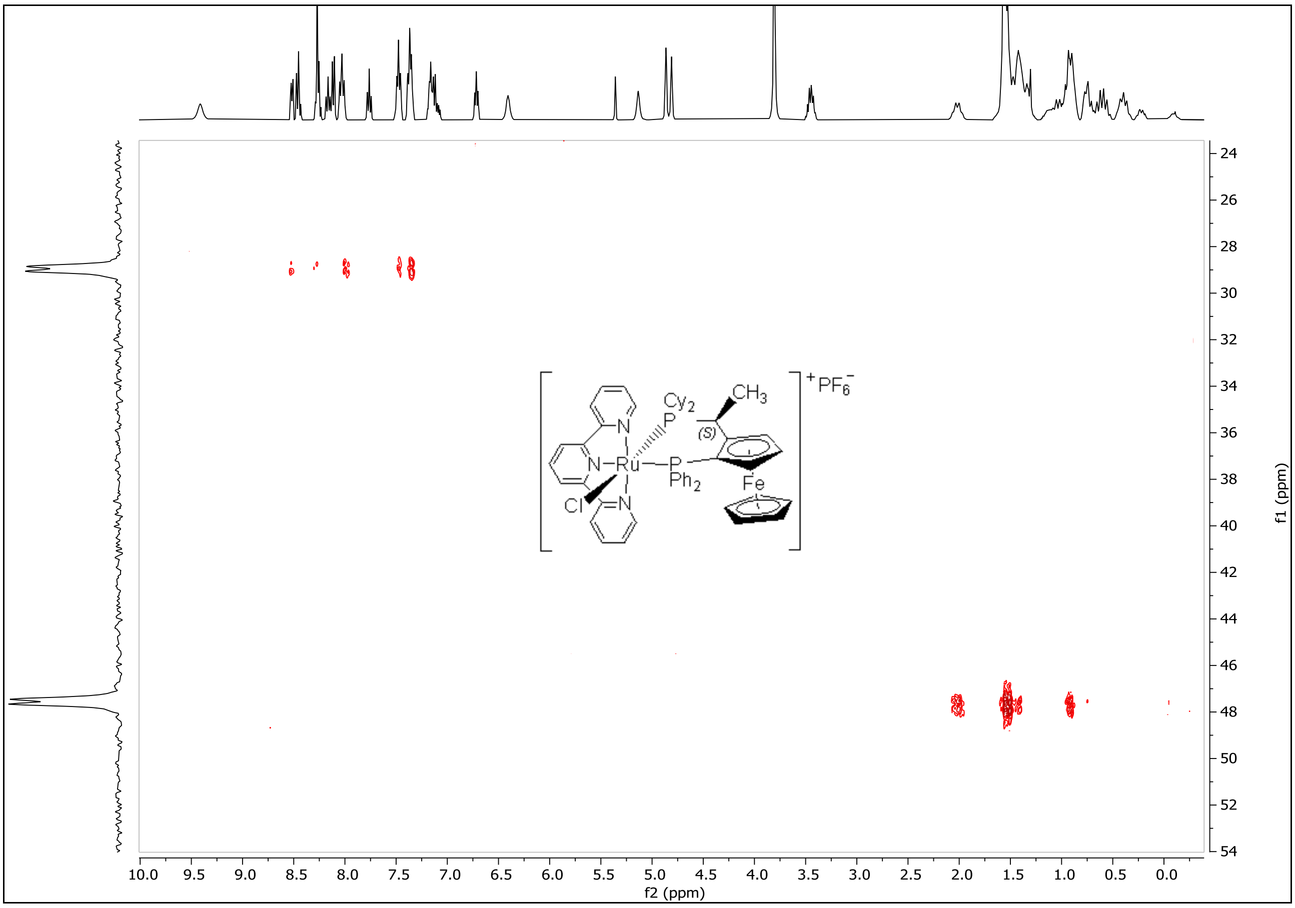

Figure S23. ^{1}H-^{31}P HMBC 2D NMR spectrum of [RuCl((*S*,*R*)-Josiphos)(tpy)]PF_6 (**4a**) in CD_2Cl_2 at 25 °C.

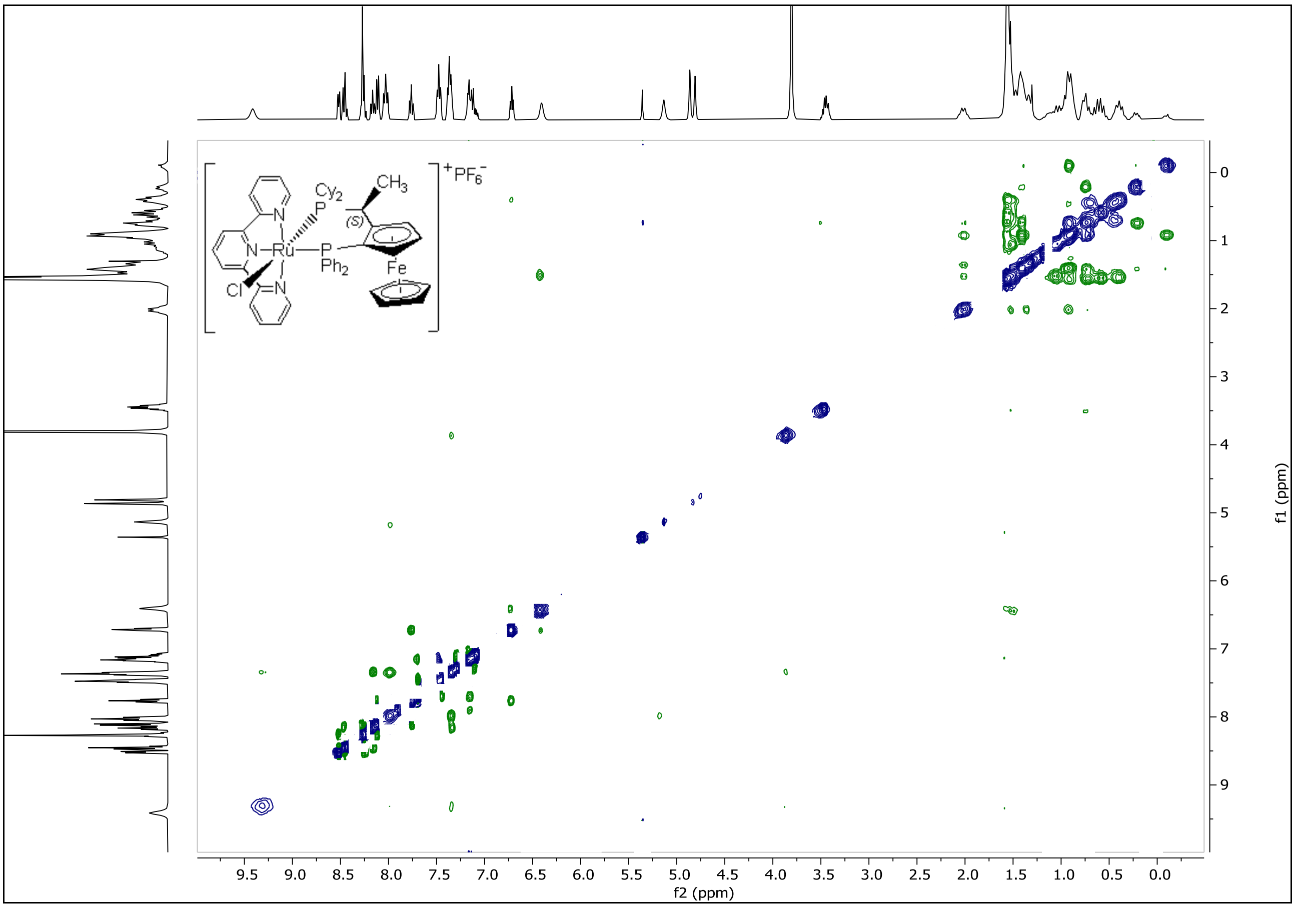

Figure S24. ^{1}H-^{1}H NOESY 2D NMR spectrum of [RuCl((*S*,*R*)-Josiphos)(tpy)]PF_6 (**4a**) in CD_2Cl_2 at 25 °C

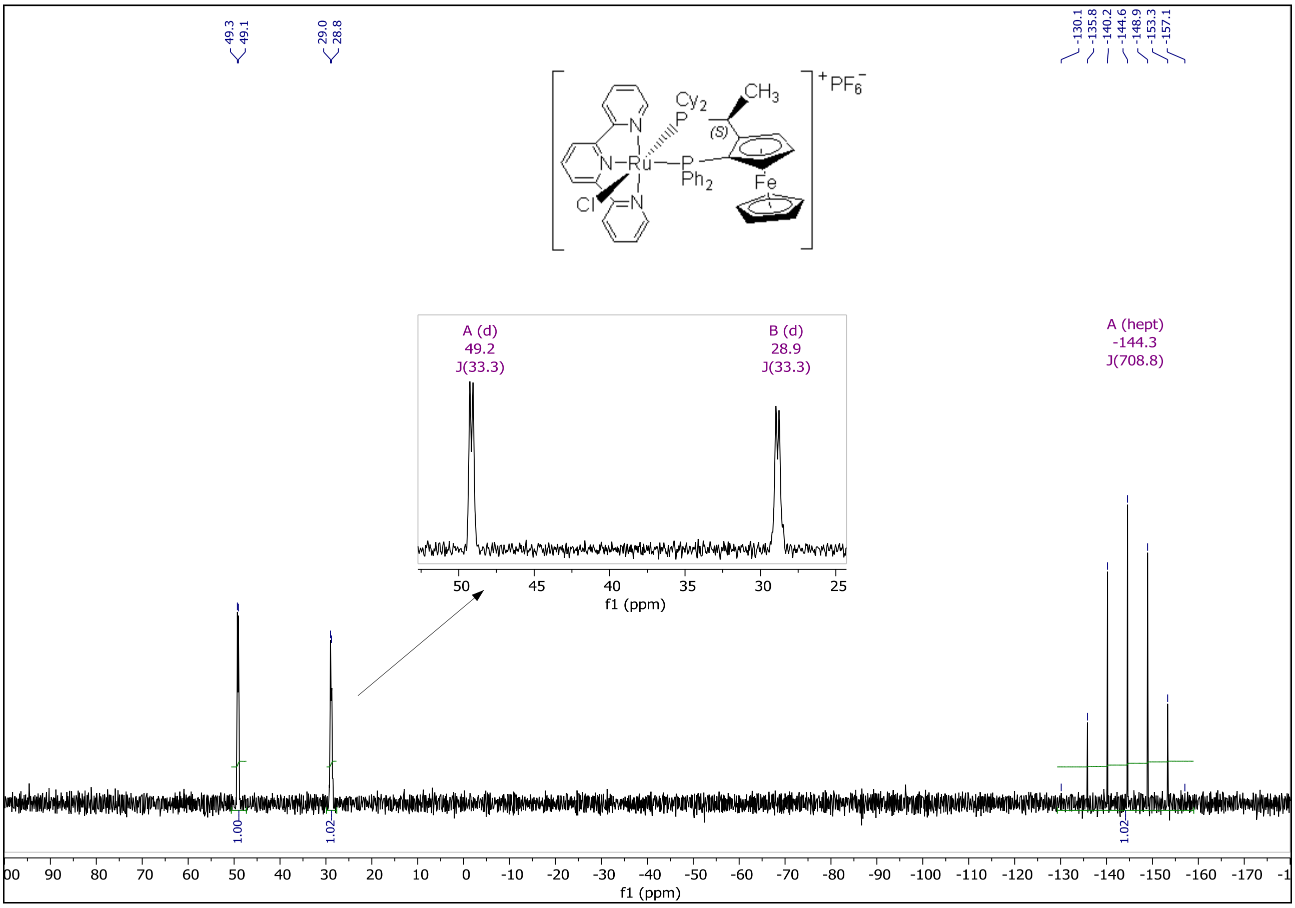

Figure S25. $^{31}P\{^1H\}$ NMR spectrum (162.0 MHz) of [RuCl((*S*,*R*)-Josiphos)(tpy)]PF_6 (**4a**) in CD_3OD at 25 °C.

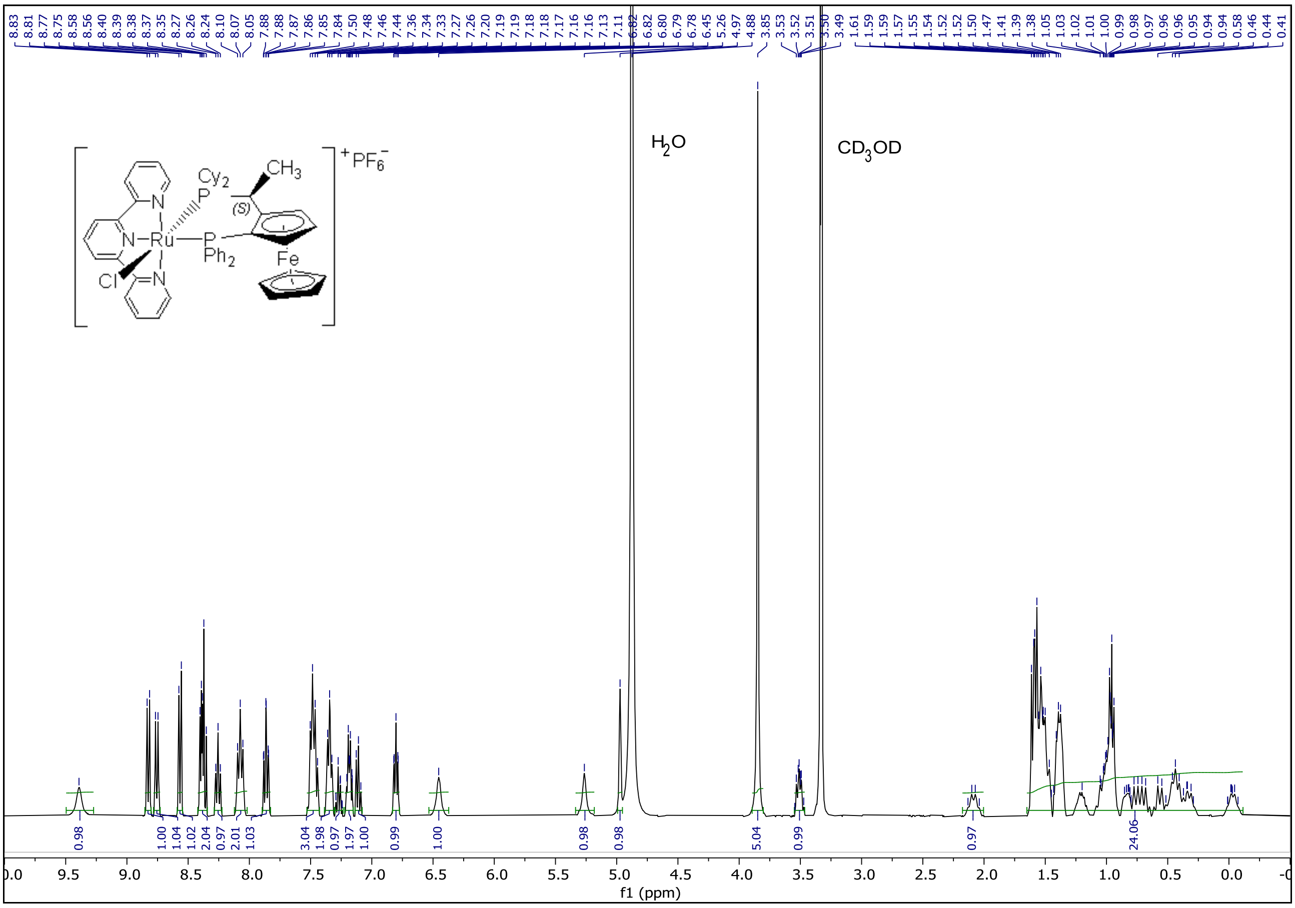

Figure S26. ^{1}H NMR spectrum (400.1 MHz) of [RuCl((*S*,*R*)-Josiphos)(tpy)]PF_6 (**4a**) in CD_3OD at 25 °C.

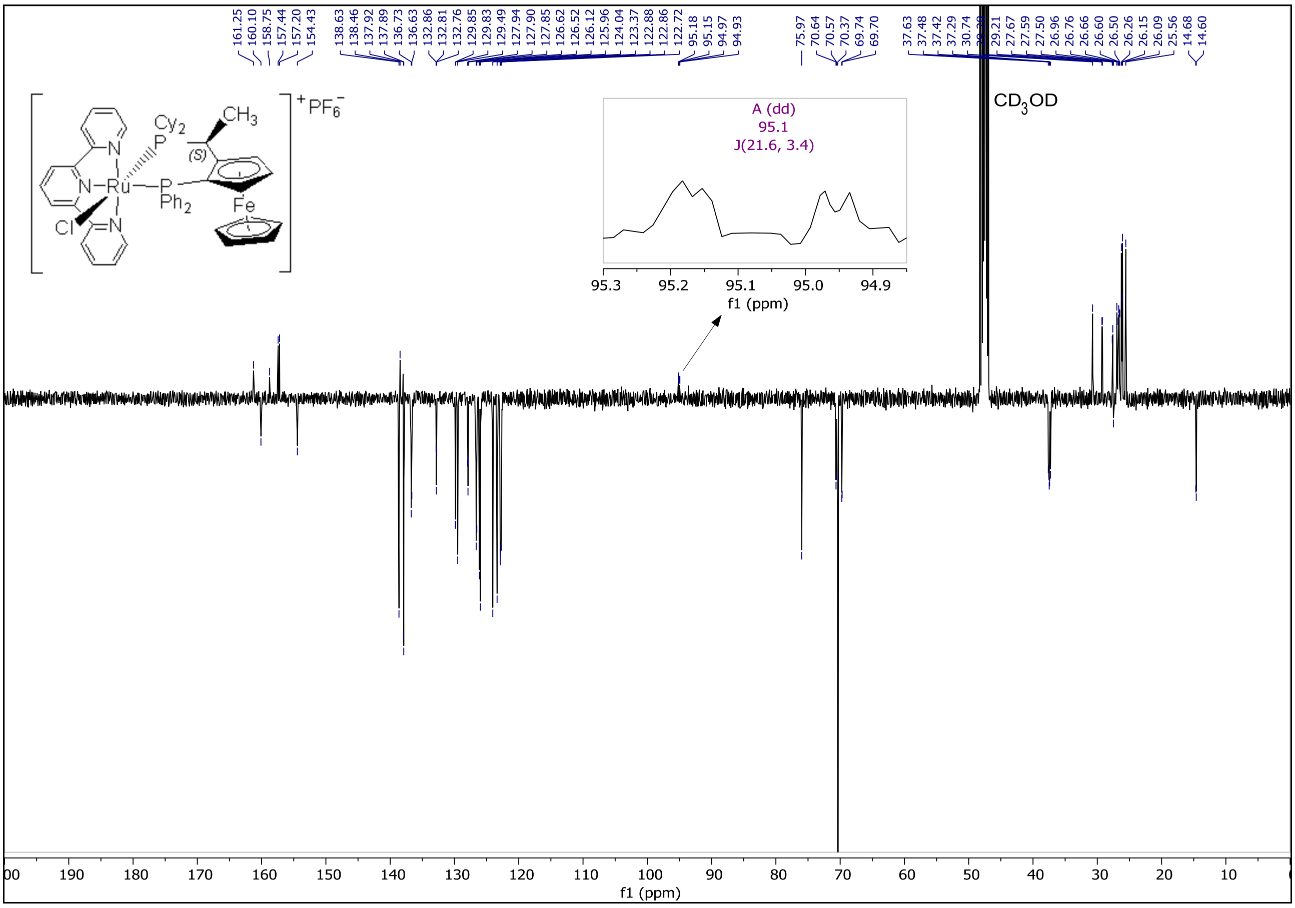

Figure S27. $^{13}C\{^1H\}$ DEPTQ NMR spectrum (100.6 MHz) of [RuCl((*S*,*R*)-Josiphos)(tpy)]PF_6 (**4a**) in CD_3OD at 25 °C.

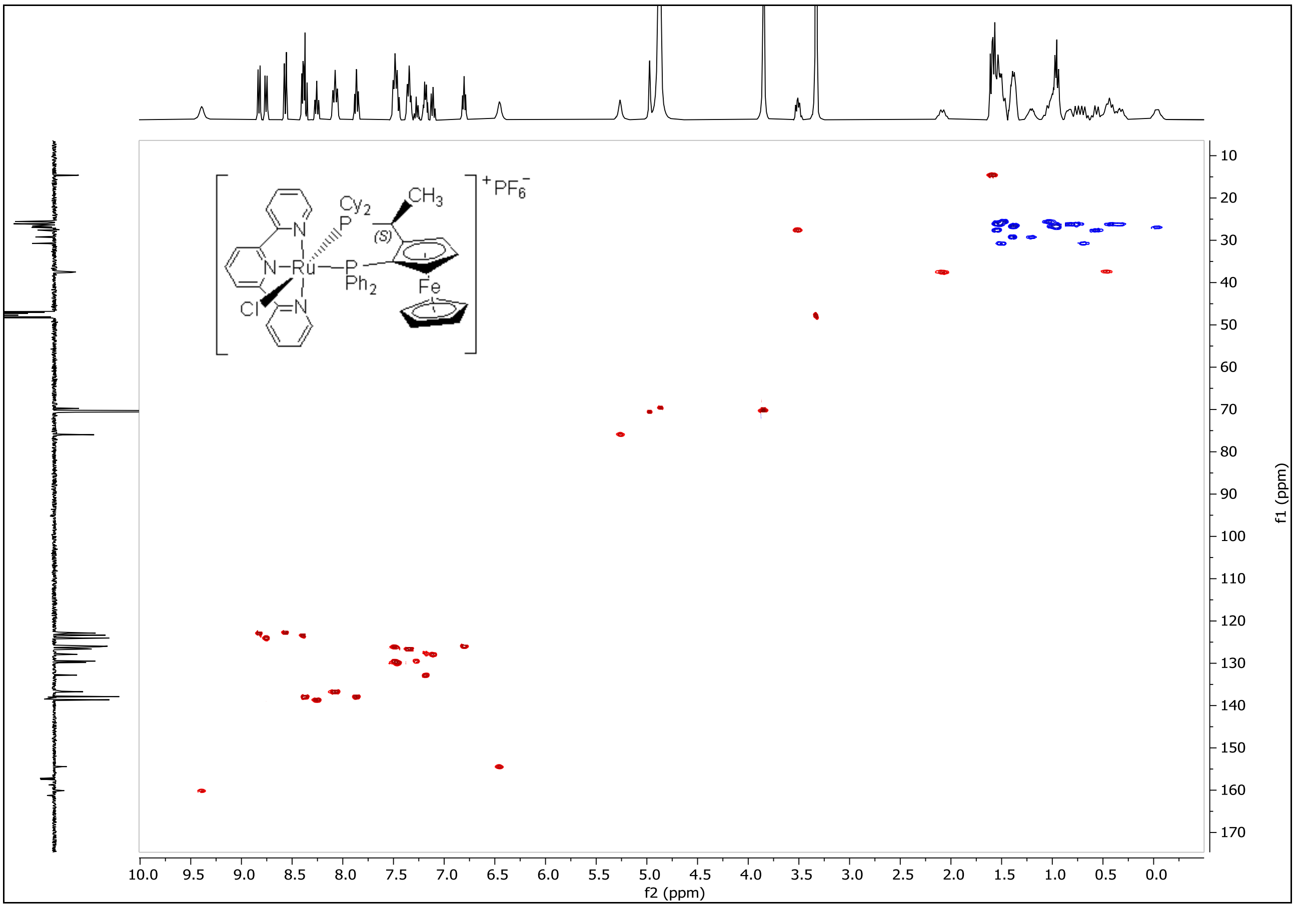

Figure S28. ^{1}H-^{13}C HSQC 2D NMR spectrum of [RuCl((*S*,*R*)-Josiphos)(tpy)]PF_6 (**4a**) in CD_3OD at 25 °C.

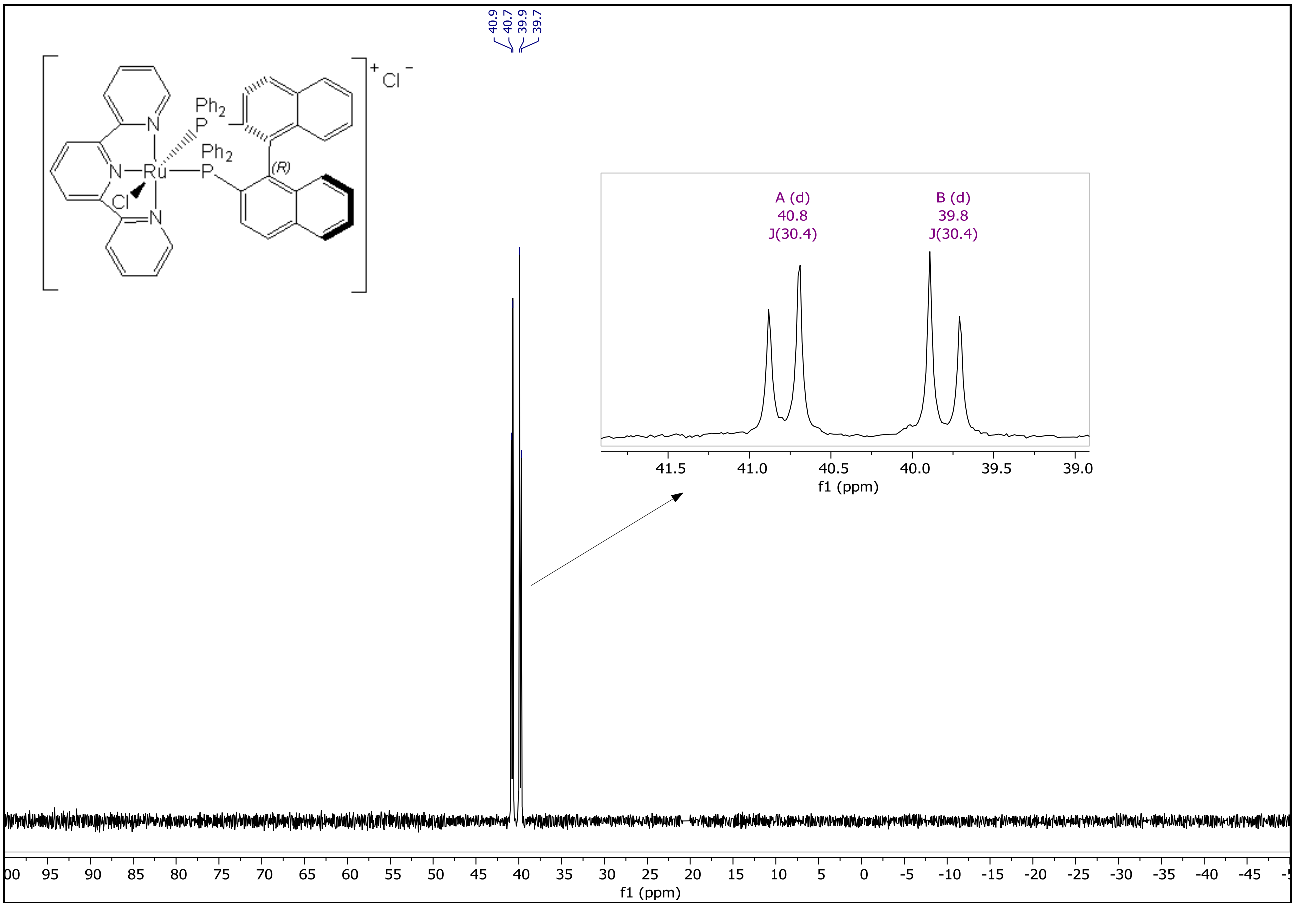

Figure S29. $^{31}P\{^1H\}$ NMR spectrum (162.0 MHz) of [RuCl((*R*)-BINAP)(tpy)]Cl (**5**) in CD_2Cl_2 at 25 °C.

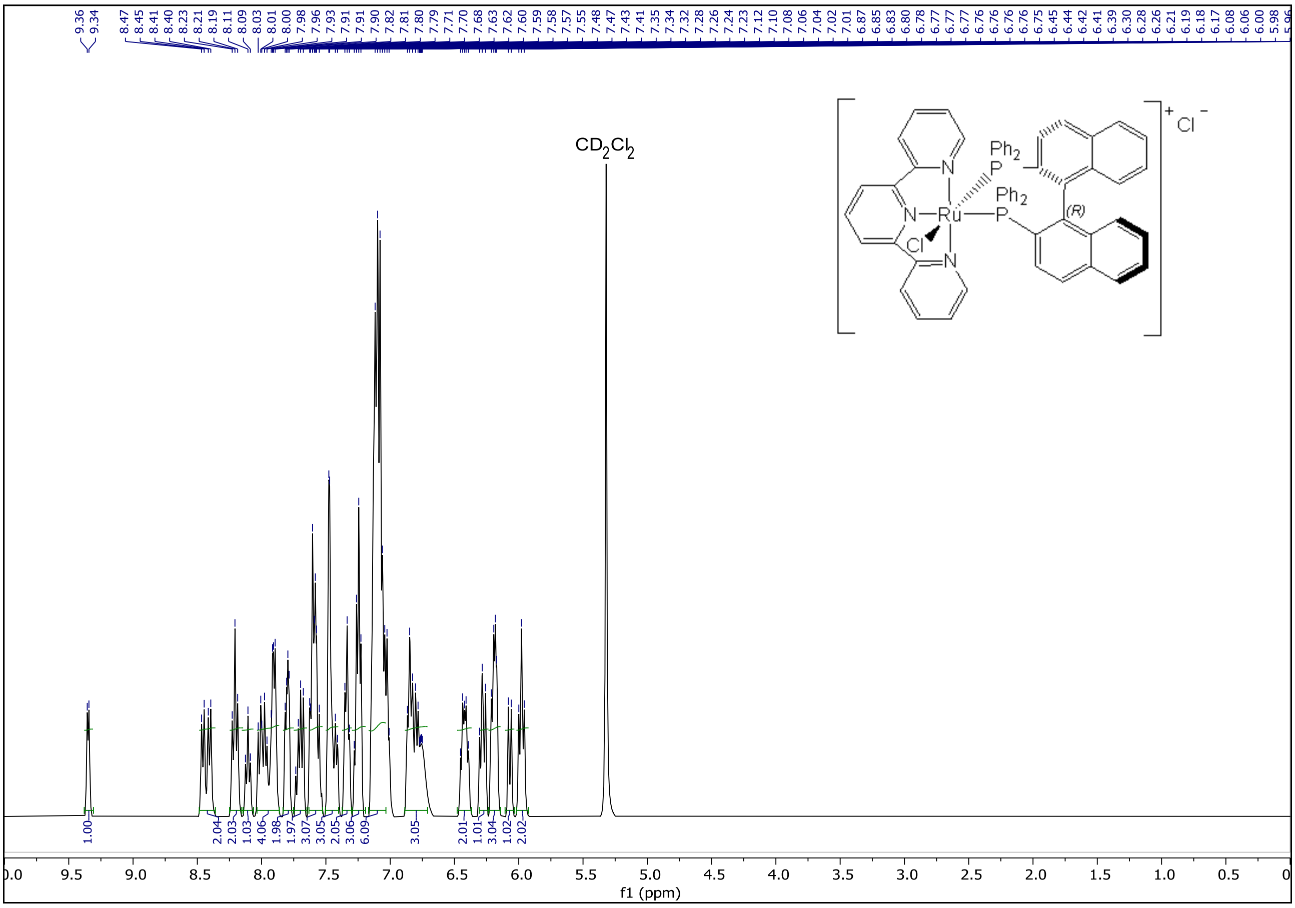

Figure S30. ^{1}H NMR spectrum (400.1 MHz) of [RuCl((*R*)-BINAP)(tpy)]Cl (**5**) in CD_2Cl_2 at 25 °C.

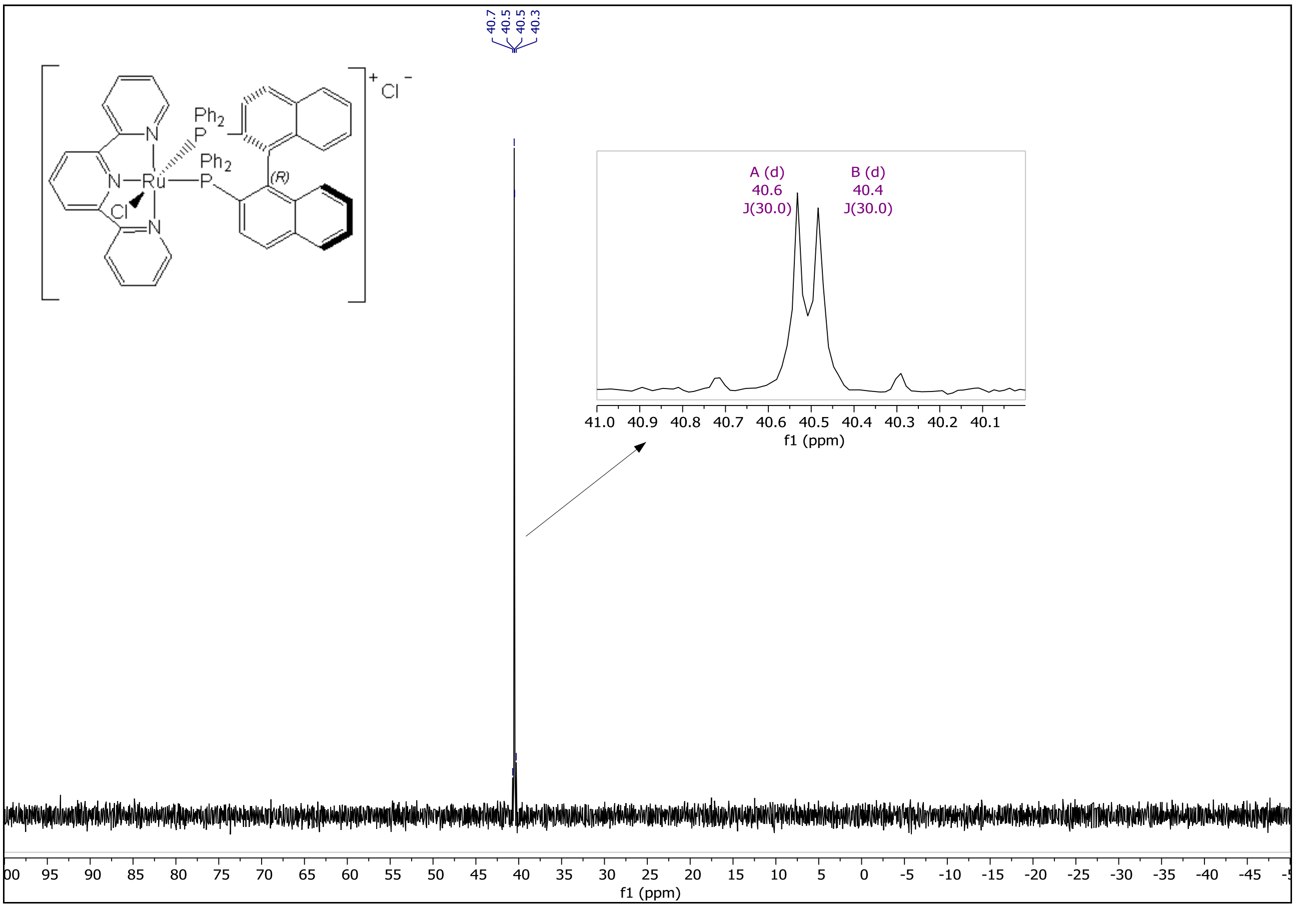

Figure S31. $^{31}P\{^{1}H\}$ NMR spectrum (162.0 MHz) of [RuCl((*R*)-BINAP)(tpy)]Cl (**5**) in CD_3OD at 25 °C.

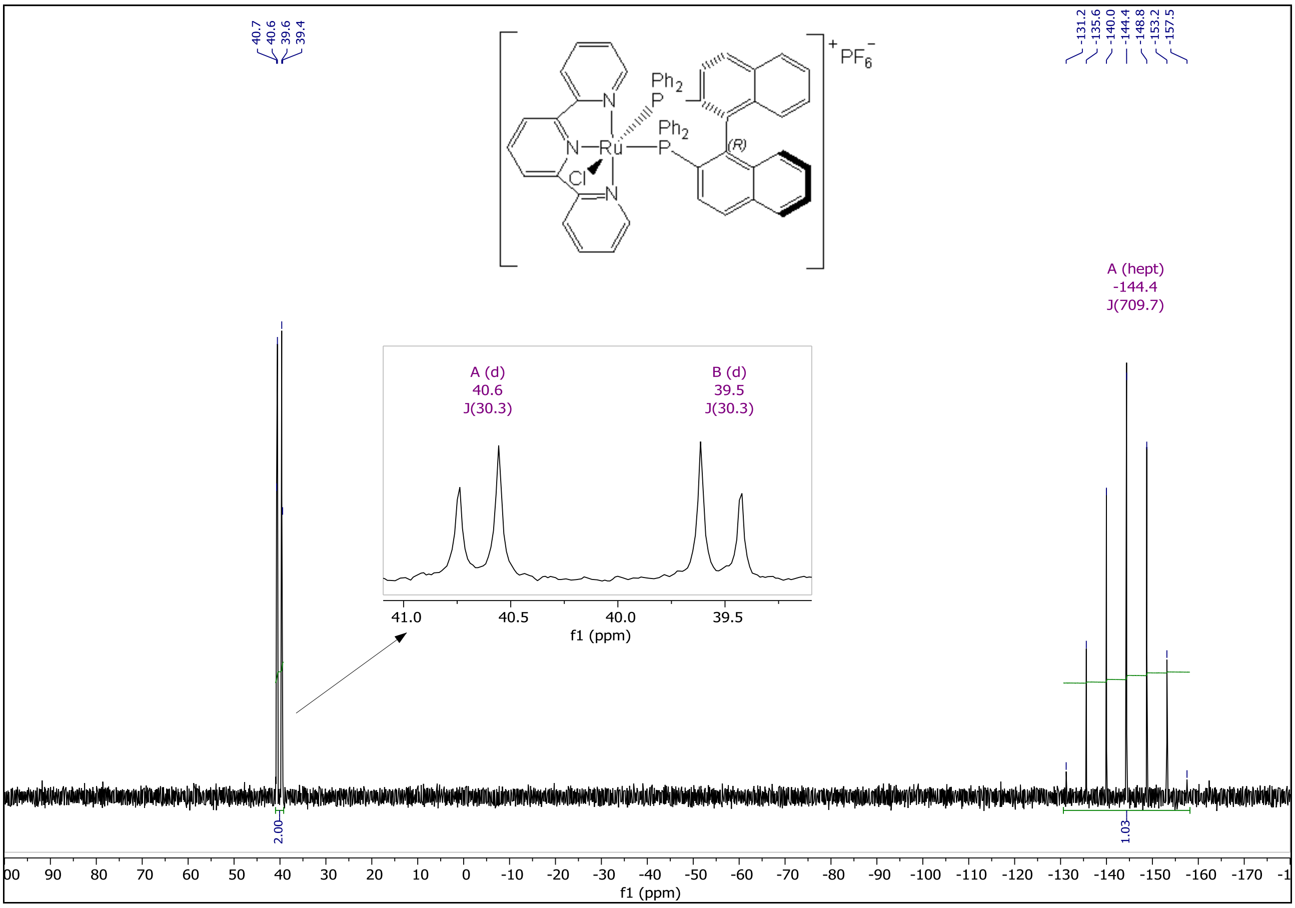

Figure S32. $^{31}P\{^{1}H\}$ NMR spectrum (162.0 MHz) of [RuCl((*R*)-BINAP)(tpy)]PF_6 (**5a**) in CD_2Cl_2 at 25 °C.

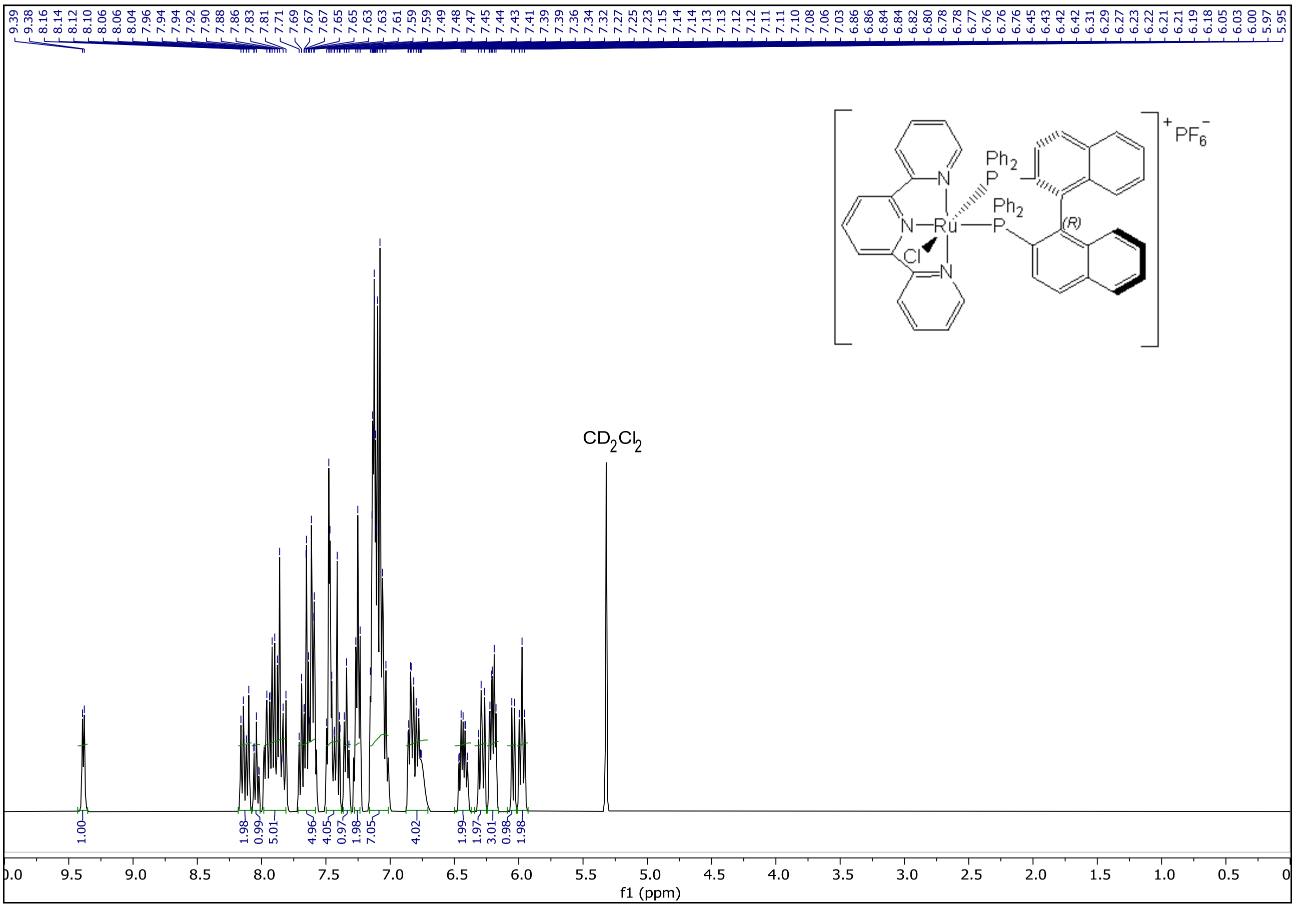

Figure S33. ^{1}H NMR spectrum (400.1 MHz) of [RuCl((*R*)-BINAP)(tpy)]PF_6 (**5a**) in CD_2Cl_2 at 25 °C.

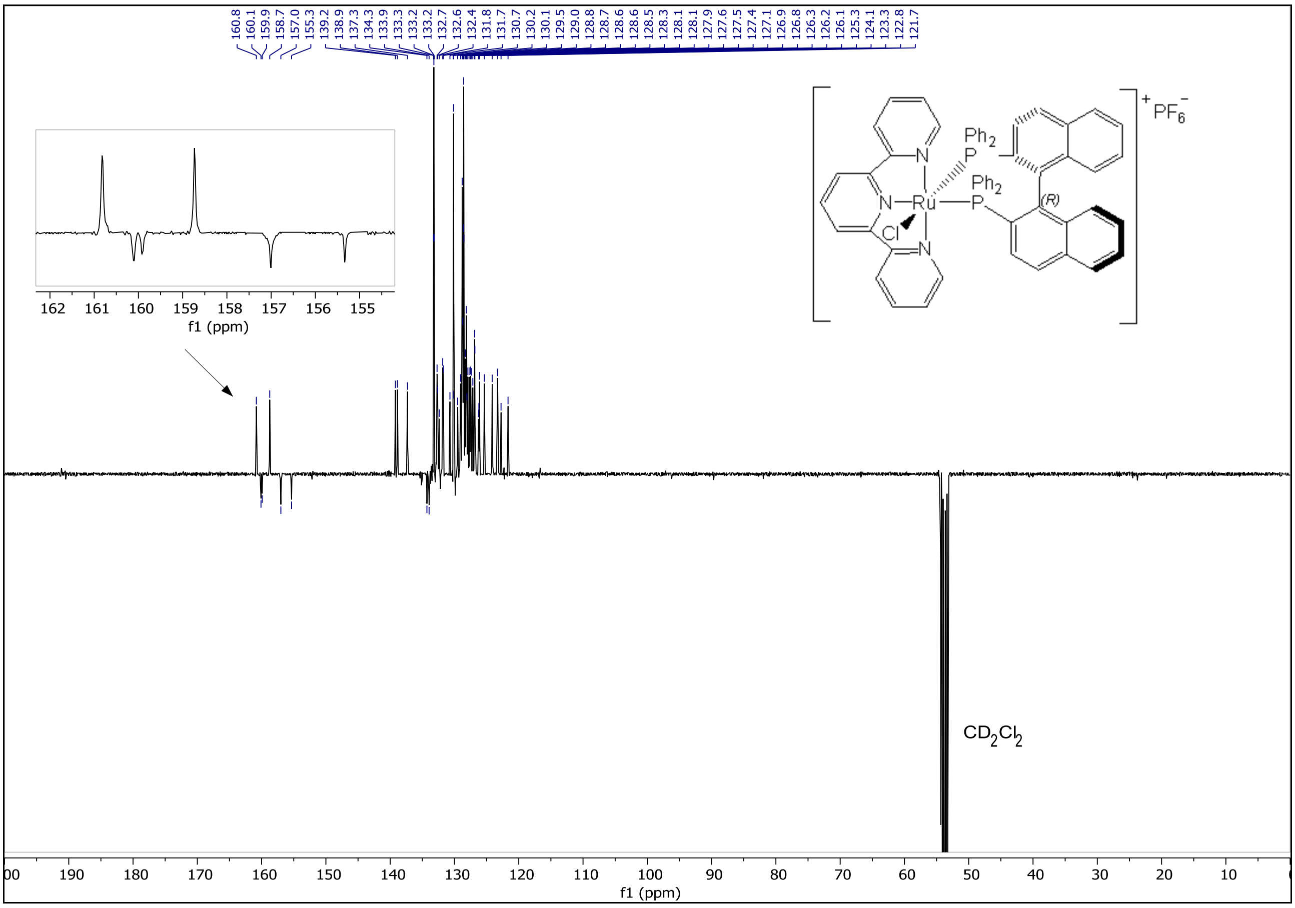

Figure S34. $^{13}C\{^{1}H\}$ DEPTQ NMR spectrum (100.6 MHz) of [RuCl((*R*)-BINAP)(tpy)]PF_6 (**5a**) in CD_2Cl_2 at 25 °C.

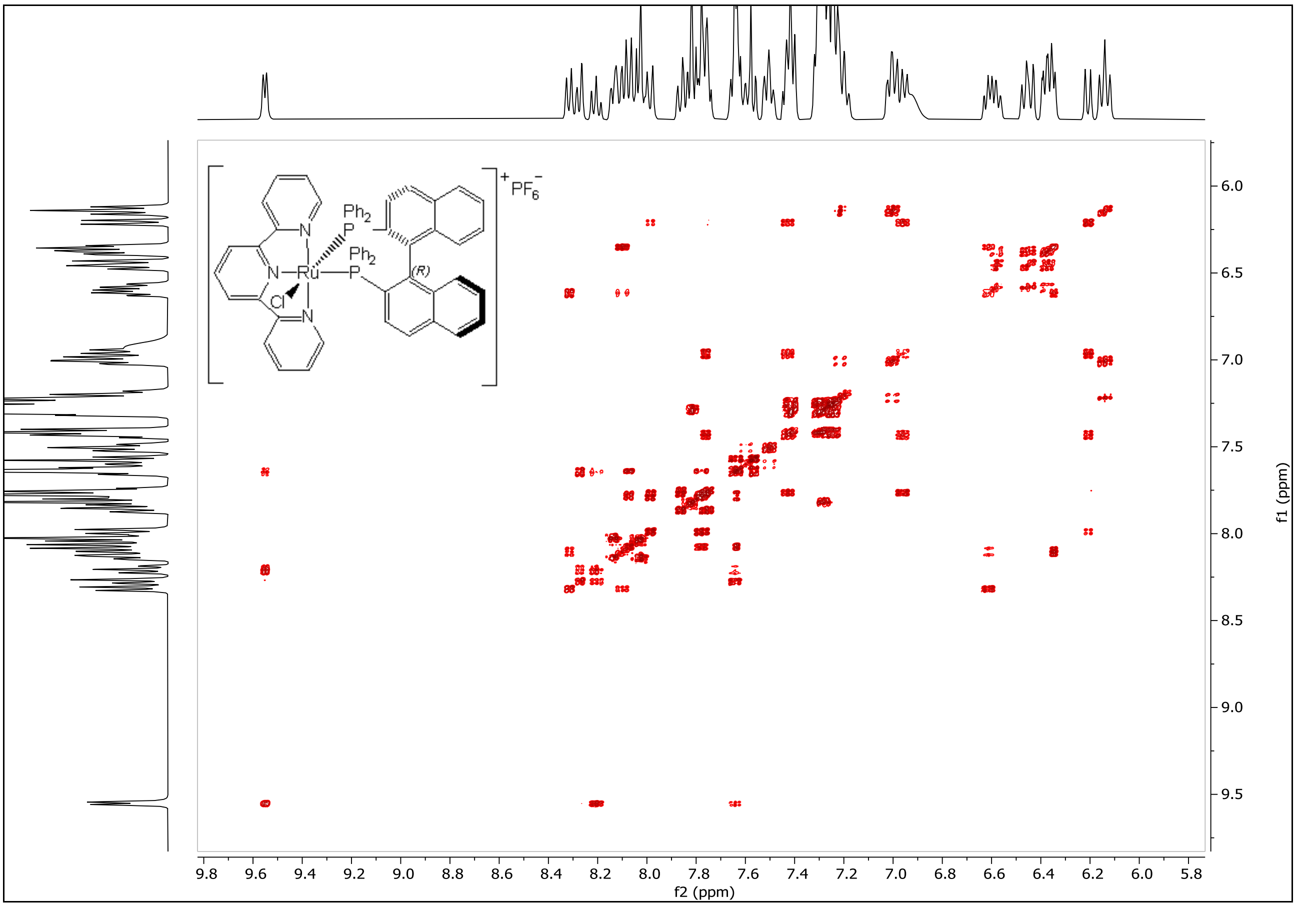

Figure S35. ^1H-^1H COSY 2D NMR spectrum of [RuCl((*R*)-BINAP)(tpy)]PF_6 (**5a**) in CD_2Cl_2 at 25 °C.

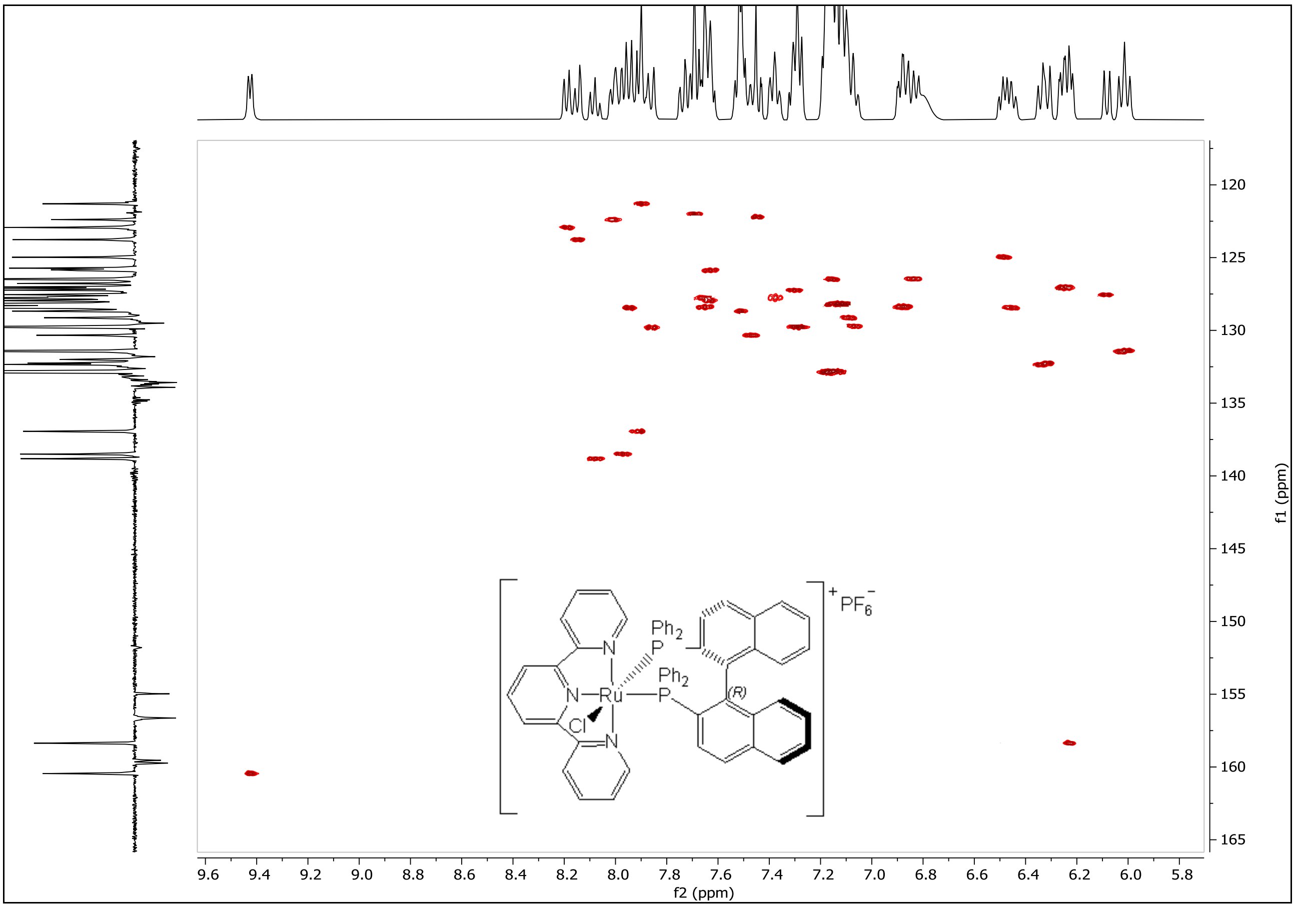

Figure S36. ^{1}H-^{13}C HSQC 2D NMR spectrum of [RuCl((*R*)-BINAP)(tpy)]PF_6 (**5a**) in CD_2Cl_2 at 25 °C.

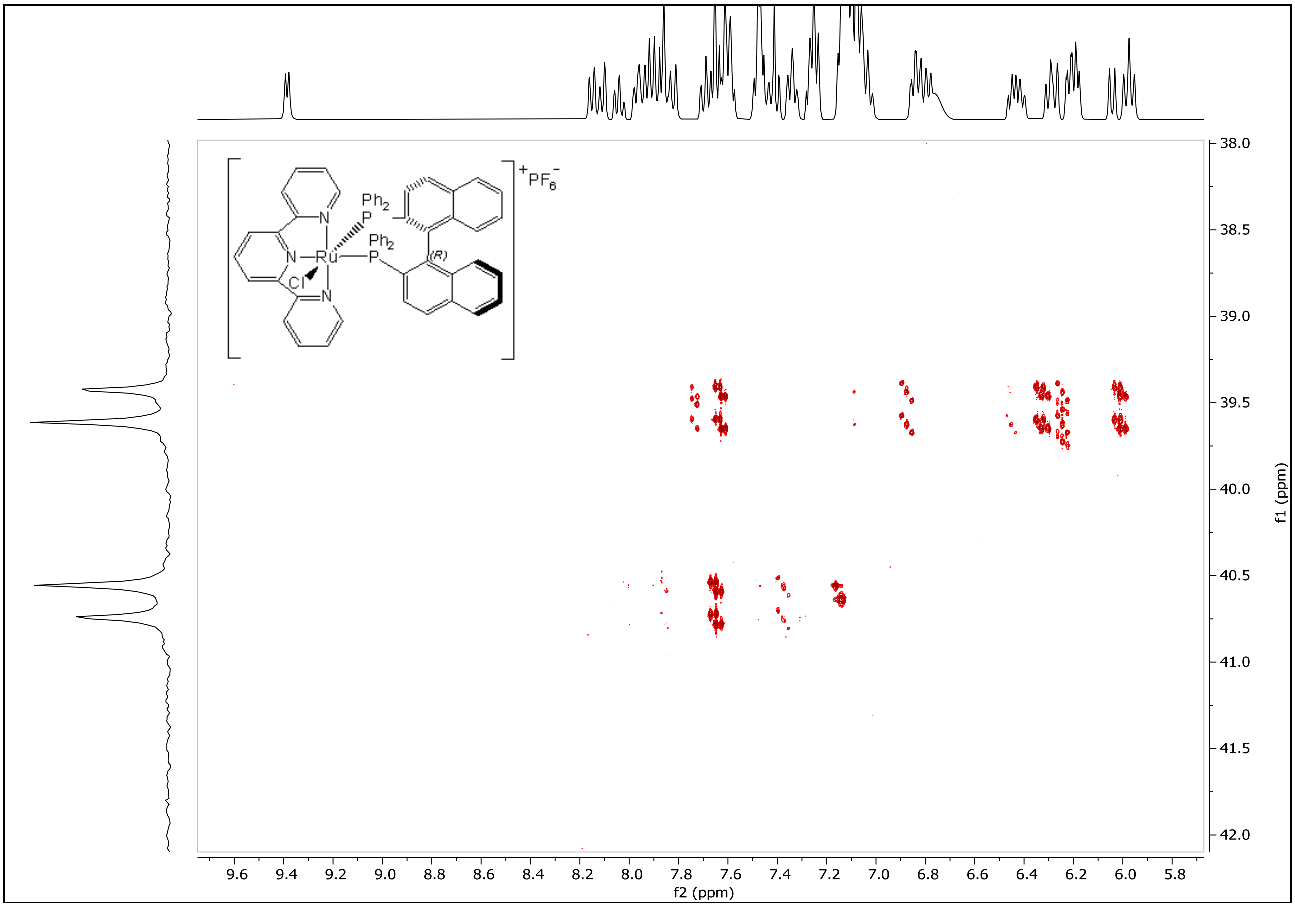

Figure S37. ^{1}H-^{31}P HMBC 2D NMR spectrum of [RuCl((*R*)-BINAP)(tpy)]PF_6 (**5a**) in CD_2Cl_2 at 25 °C.

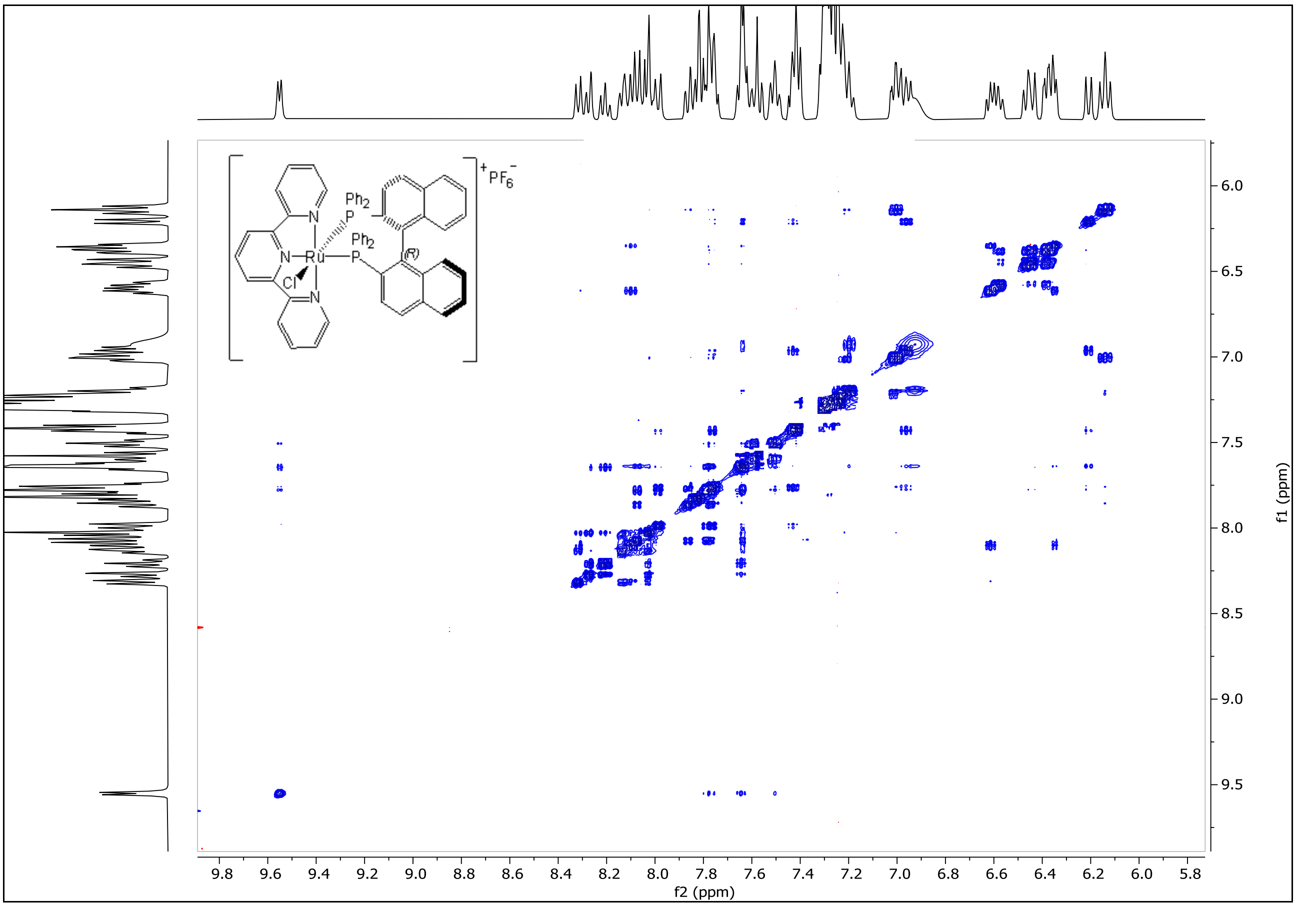

Figure S38. ^{1}H-^{1}H NOESY 2D NMR spectrum of [RuCl((*R*)-BINAP)(tpy)]PF_6 (**5a**) in CD_2Cl_2 at 25 °C.

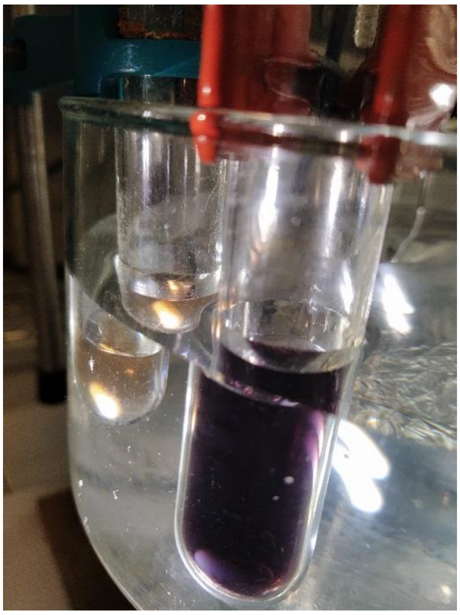

(A)

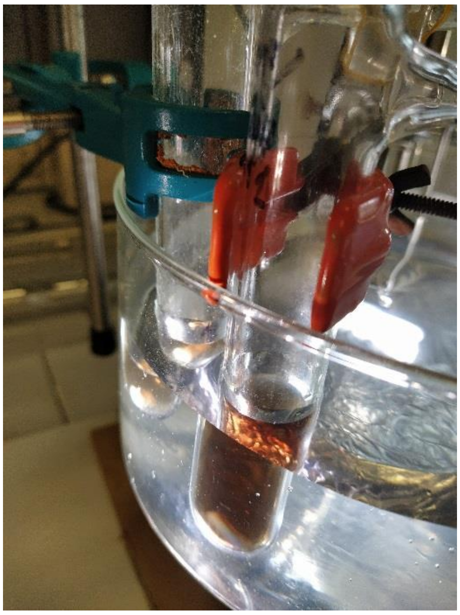

(B)

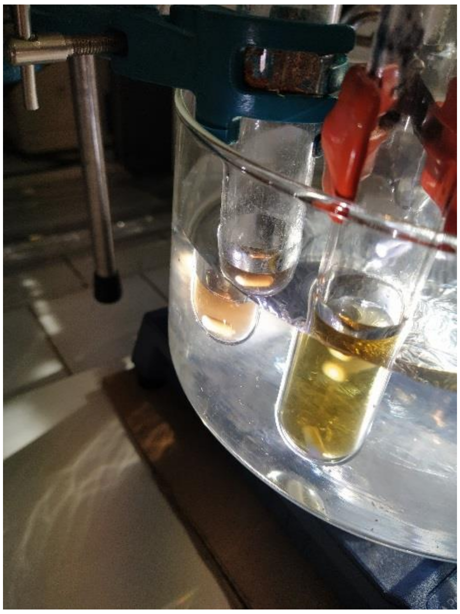

(C)

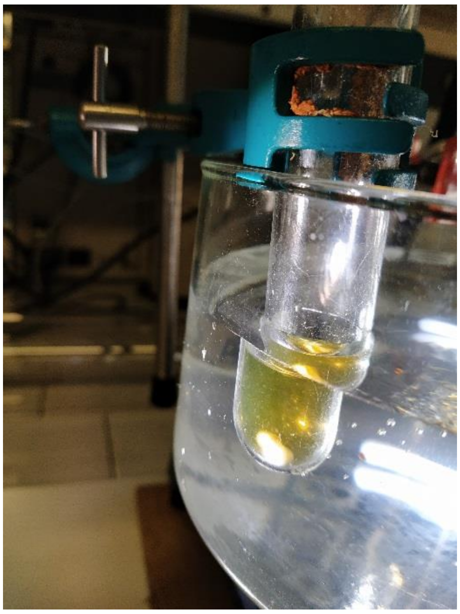

(D)

Figure S39. Photographs of the color evolution of the reaction mixtures during the photocatalytic TH of carbonyl compounds in 2-propanol with NaO*i*Pr 2 mol% at 30 °C. Color after 1 min (**A**), 10 min (**B**), 15 min (**C**), and 25 min (**D**). Comparted with the starting material (prepared in the dark) contained in the schlenk tube with the orange solution in the first three pictures. The timing of color changes are purely indicative and depend from the nature of the substrate and catalyst, and the S/C employed.

Table S1. Further data regarding the Photocatalytic TH of ketones and aldehydes (0.1 M) with complexes **1**, **2**, and **3a** at 30 °C in the presence of NaO*i*Pr 2 mol% (S/C = 1000).

Entry	Complex	Substrate	Solvent	Time[a] [h]	Conv. [b] [%]	ee[b] [%]	By-products[b] [%]
1	**1**	**f**	iPrOH	18	2	-	-
2	**2**	**f**	iPrOH	18	3	-	-
3	**2**	**g**	iPrOH	8	1	-	-
4	**2**	**h**	iPrOH	18	22[c]	-	16[c]
5	**2**	**i**	iPrOH	18	31[d]	-	9[d]
6	**3a**	**k**	iPrOH/MeOH (1:1)	25	0.1	-	-
7	**3a**	**l**	iPrOH/MeOH (1:1)	12	0.2	-	-
8	**3a**	**m**	iPrOH/MeOH (1:1)	36	16	4*R*	-

[a] Irradiation hours. [b] The conversions and *e.e.* were determined by GC analysis. [c] Levoglucosanol with an *erythro*/*threo* ratio 1 : 1.7 and uncharacterized by-products. [d] Mixture of 3-phenylpropan-1-ol (16%), 3-phenyl-2-propenol (cinnamyl alcohol, 6%) and uncharacterized by-products.

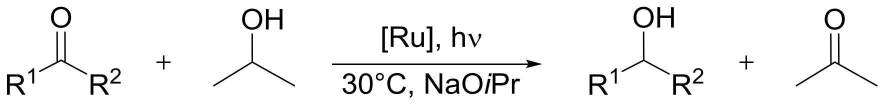

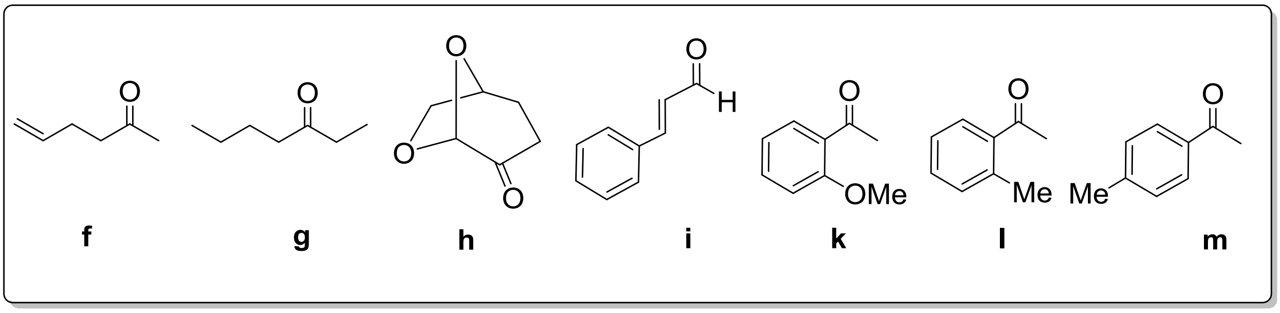

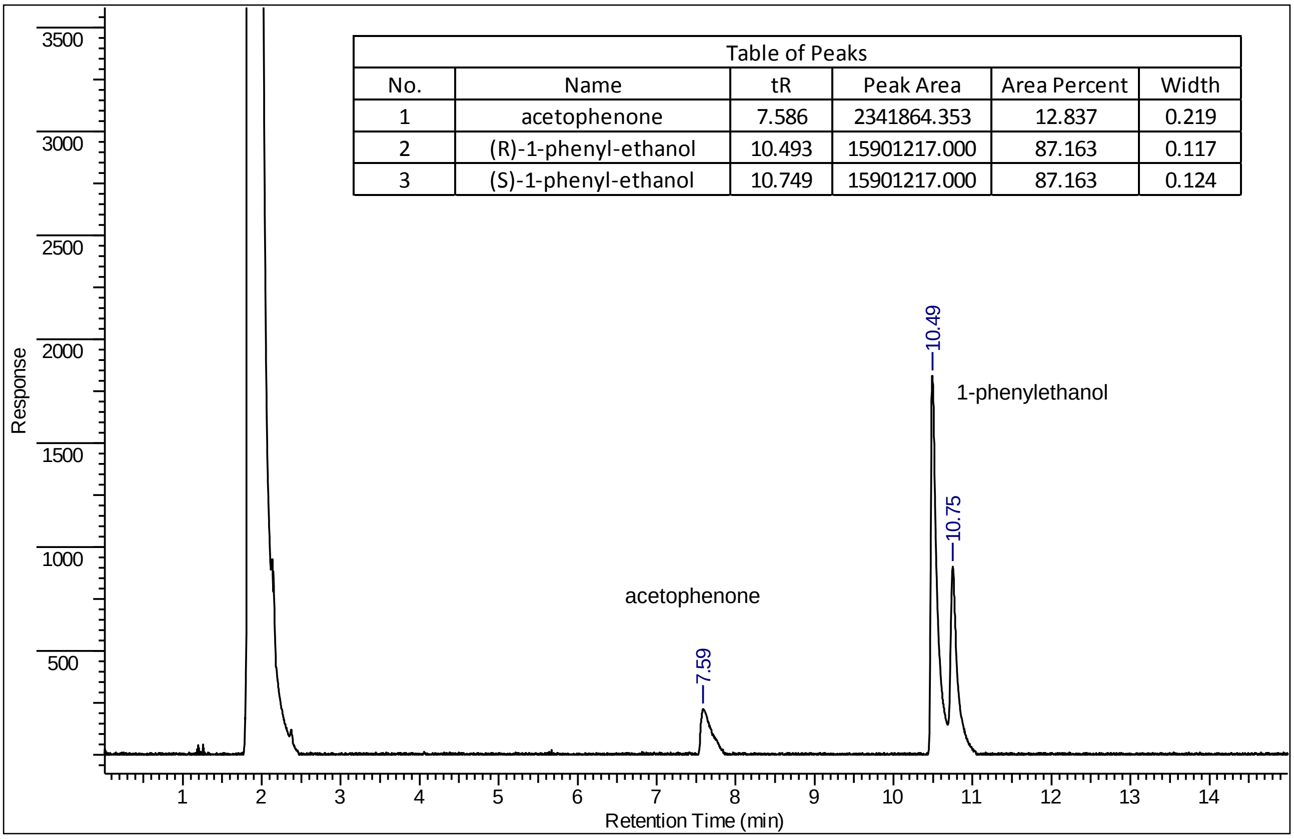

Table of Peaks

No.	Name	tR	Peak Area	Area Percent	Width
1	acetophenone	7.586	2341864.353	12.837	0.219
2	(R)-1-phenyl-ethanol	10.493	15901217.000	87.163	0.117
3	(S)-1-phenyl-ethanol	10.749	15901217.000	87.163	0.124

Figure S40. GC-FID chromatogram of the reaction mixture of the enantioselective photocatalytic TH of acetophenone **a** in 2-propanol/MeOH 1:1 (v/v) at 30 °C and NaO*i*Pr 2 mol% promoted by complex **3** at S/C 1000 after 24 h of overall irradiation. GC analyses were performed with a Varian CP-3380 gas chromatograph equipped with a 25 m length MEGADEX-ETTBDMS-β chiral column with hydrogen (5 psi) as the carrier gas and flame ionization detector (FID). The injector and detector temperature was 250 °C, with initial T = 95 °C ramped to 140 °C at 3 °C/min for a total of 15 min of analysis.

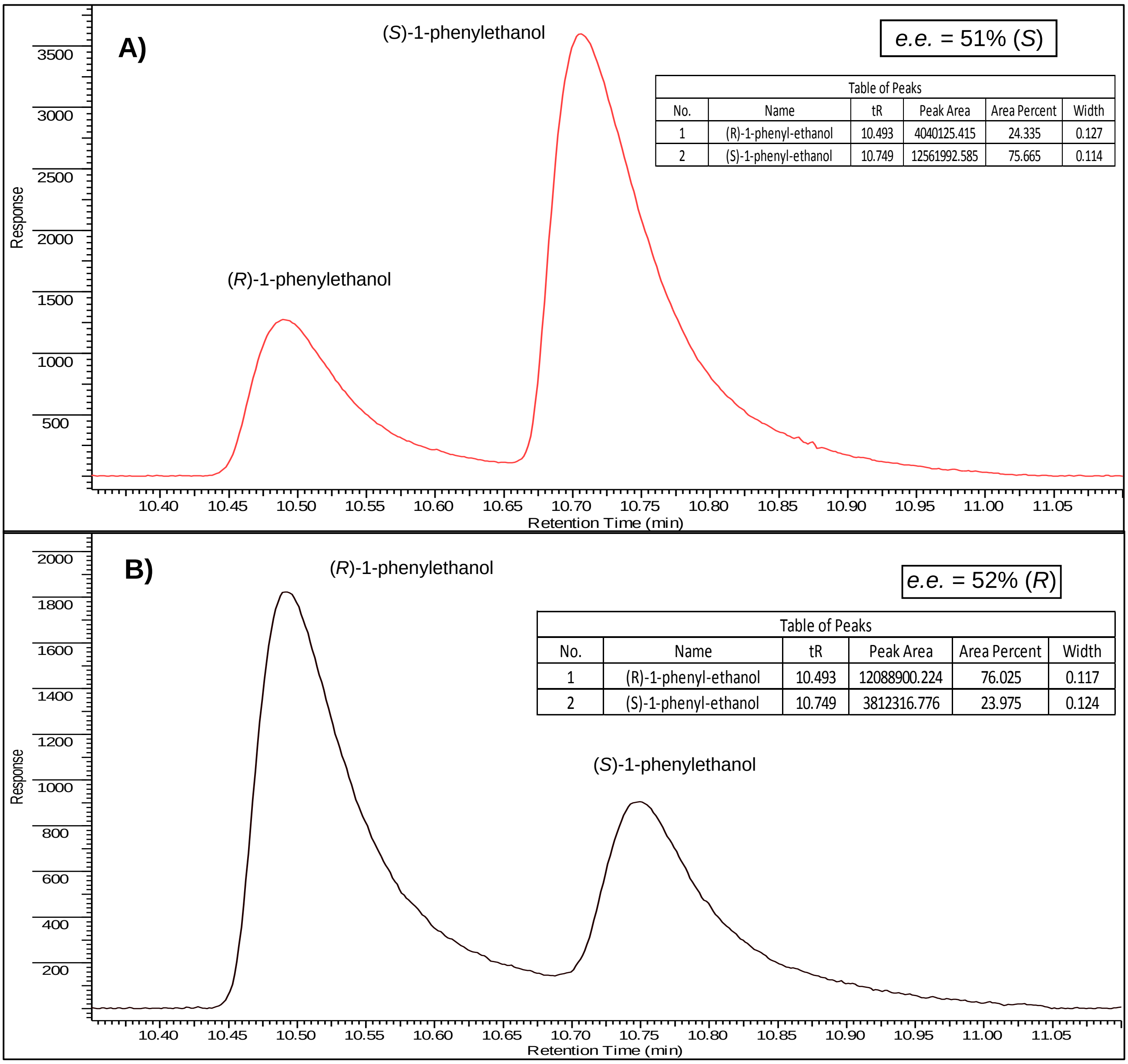

Figure S41. Comparison between the GC-FID chromatograms of the enantioselective photocatalytic TH of acetophenone **a** in 2-propanol/MeOH 1:1 (v/v) with NaO*i*Pr 2 mol% at 30 °C promoted by complex **2** (A) and **3** (B) at S/C 1000.

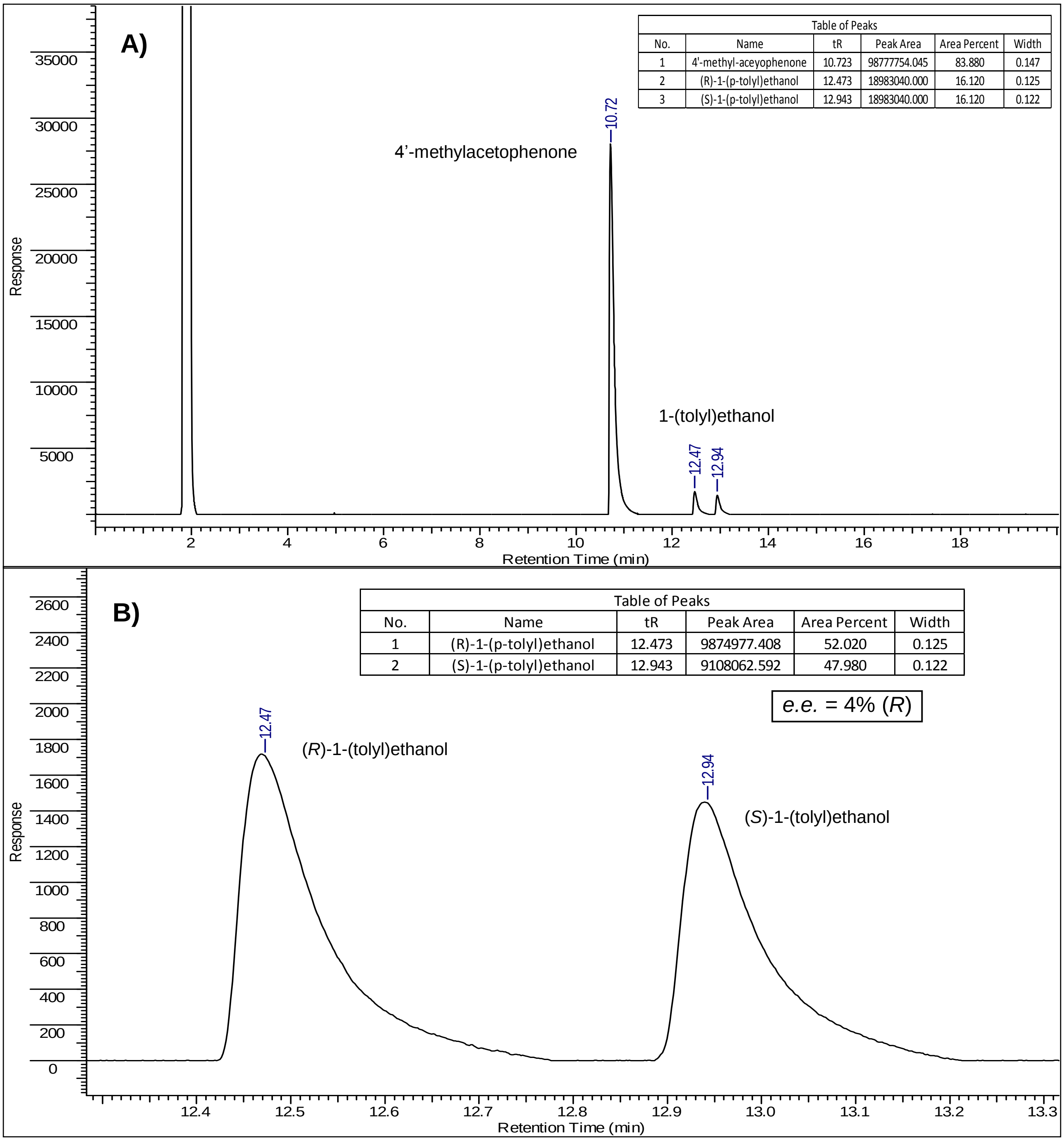

Table of Peaks (A)

No.	Name	tR	Peak Area	Area Percent	Width
1	4'-methyl-aceyophenone	10.723	98777754.045	83.880	0.147
2	(R)-1-(p-tolyl)ethanol	12.473	18983040.000	16.120	0.125
3	(S)-1-(p-tolyl)ethanol	12.943	18983040.000	16.120	0.122

Table of Peaks (B)

No.	Name	tR	Peak Area	Area Percent	Width
1	(R)-1-(p-tolyl)ethanol	12.473	9874977.408	52.020	0.125
2	(S)-1-(p-tolyl)ethanol	12.943	9108062.592	47.980	0.122

Figure S42. GC-FID chromatogram (A) of the reaction mixture of the enantioselective photocatalytic TH of 4’-methyl-acetophenone **m** in 2-propanol/MeOH 1:1 (v/v) at 30 °C and NaO*i*Pr 2 mol% promoted by complex **3** at S/C 1000 after 24 h of overall irradiation, and detailed of the peaks of (*R*)- and (*S*)-1-(tolyl)ethanol (B). GC analyses were performed with a Varian CP-3380 gas chromatograph equipped with a 25 m length MEGADEX-ETTBDMS-β chiral column with hydrogen (5 psi) as the carrier gas and flame ionization detector (FID). The injector and detector temperature was 250 °C, with initial T = 95 °C ramped to 140 °C at 3 °C/min for a total of 20 min of analysis.

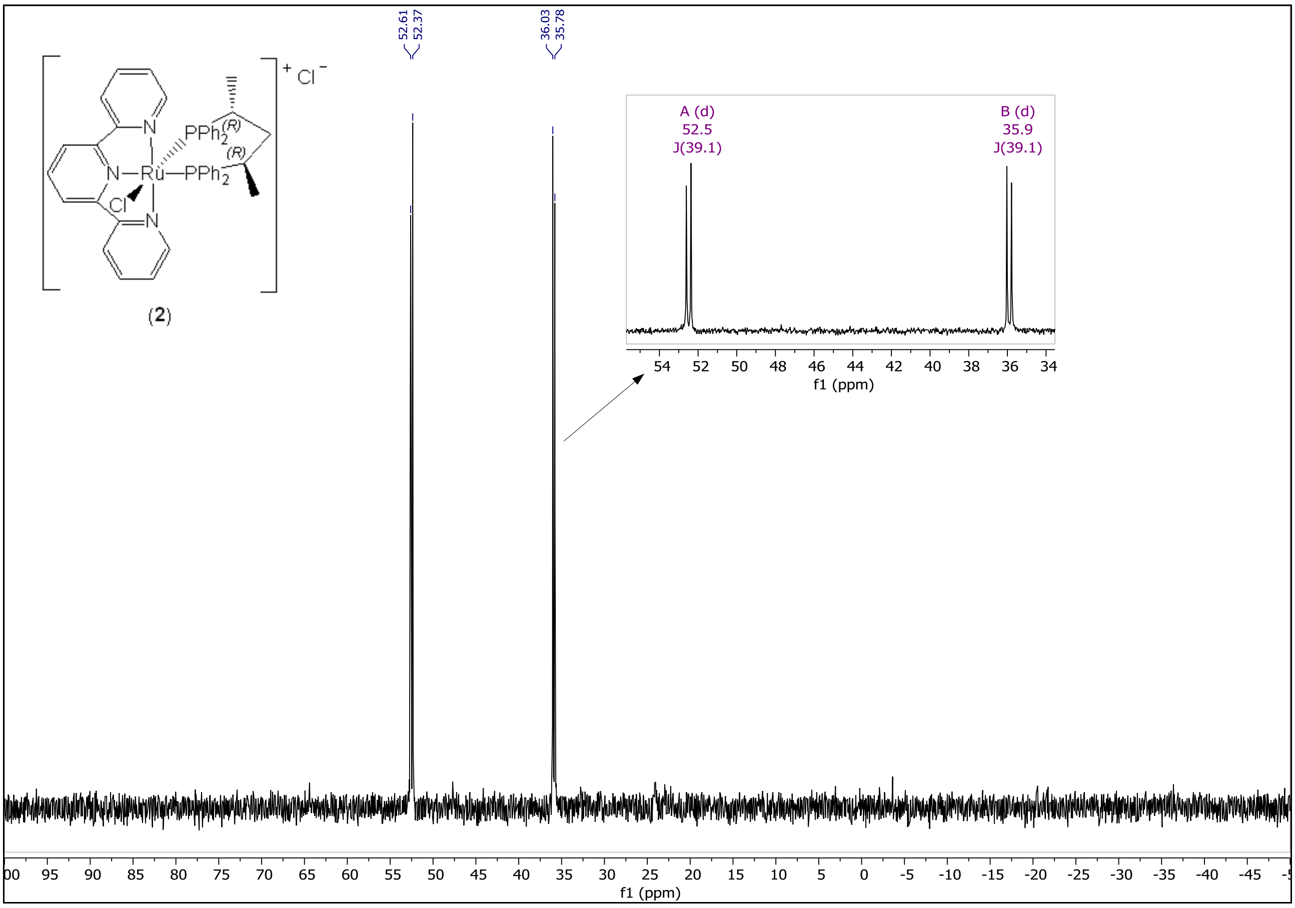

Figure S43. $^{31}P\{^{1}H\}$ NMR spectrum (162.0 MHz) of [RuCl((*R*,*R*)-Skewphos)(tpy)]Cl (**2**) in 2-propanol-d^8 at 25 °C.

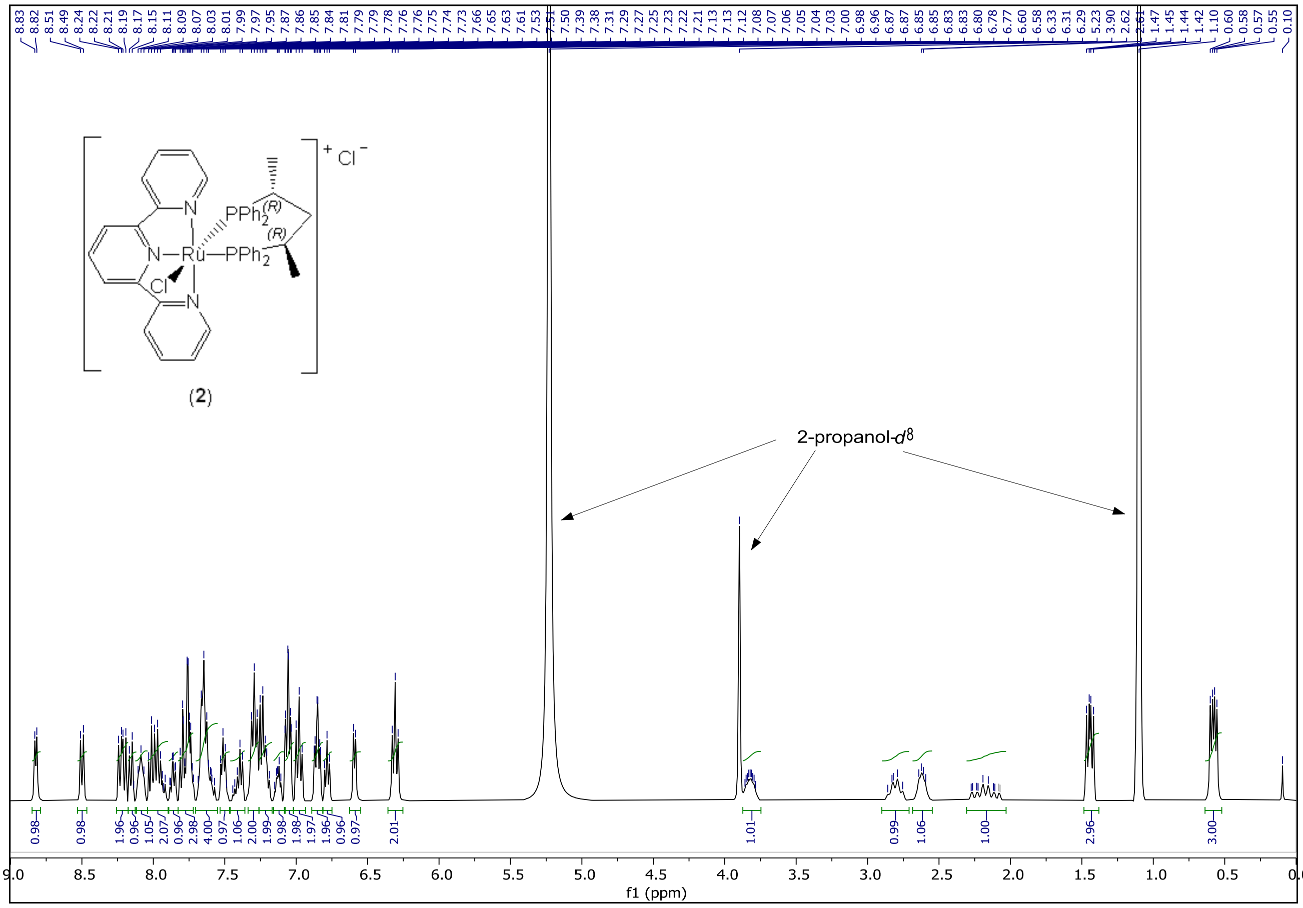

Figure S44. ^{1}H NMR spectrum (400.1 MHz) of [RuCl((*R*,*R*)-Skewphos)(tpy)]Cl (**2**) in 2-propanol-d^{8} at 25 °C.

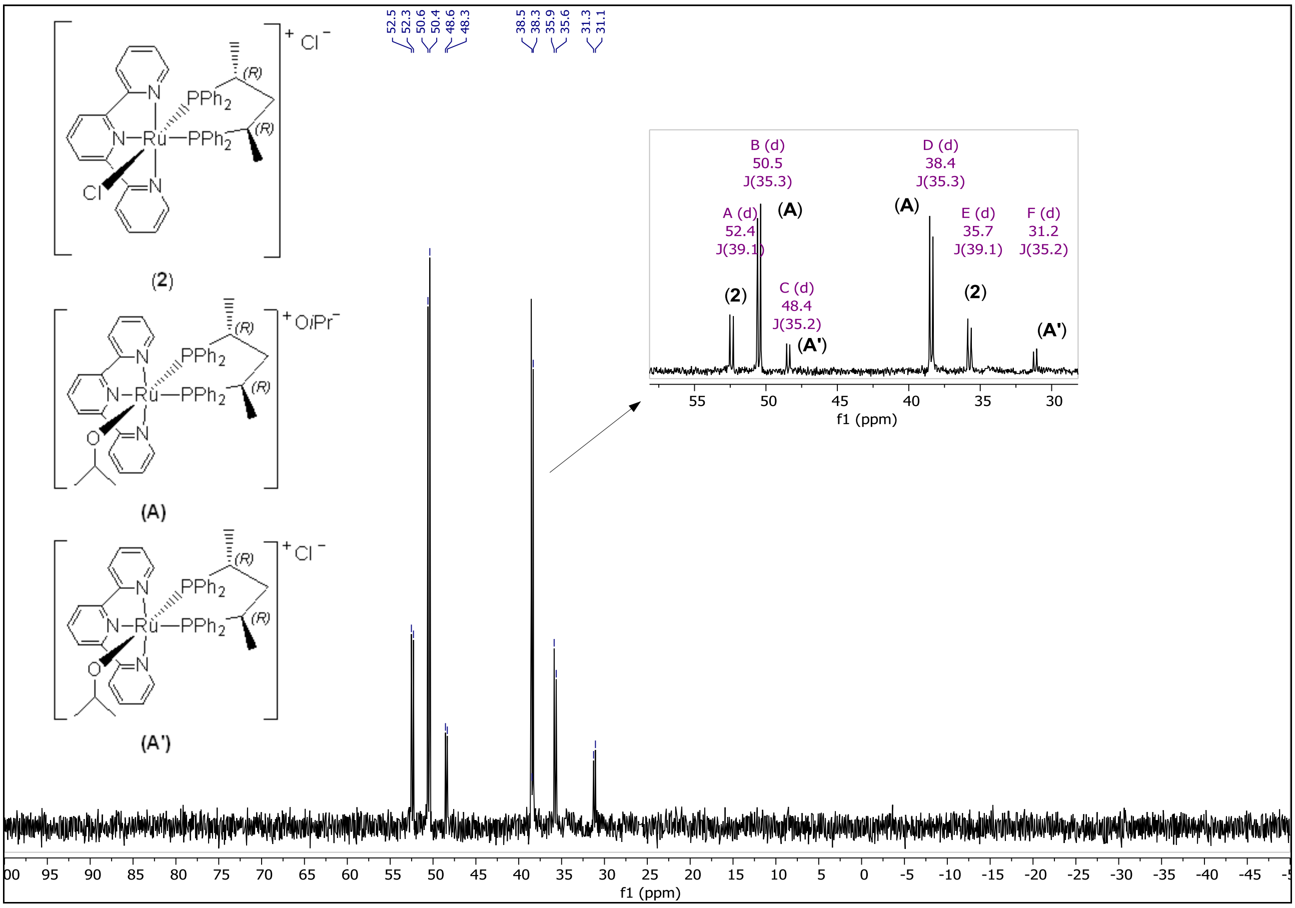

Figure S45. $^{31}P\{^1H\}$ NMR spectrum (162.0 MHz) of the mixture obtained from [RuCl((*R*,*R*)-Skewphos)(tpy)]Cl (**2**) by addition of NaO*i*Pr (3 equiv) in 2-propanol-d^8 at 25 °C in the dark.

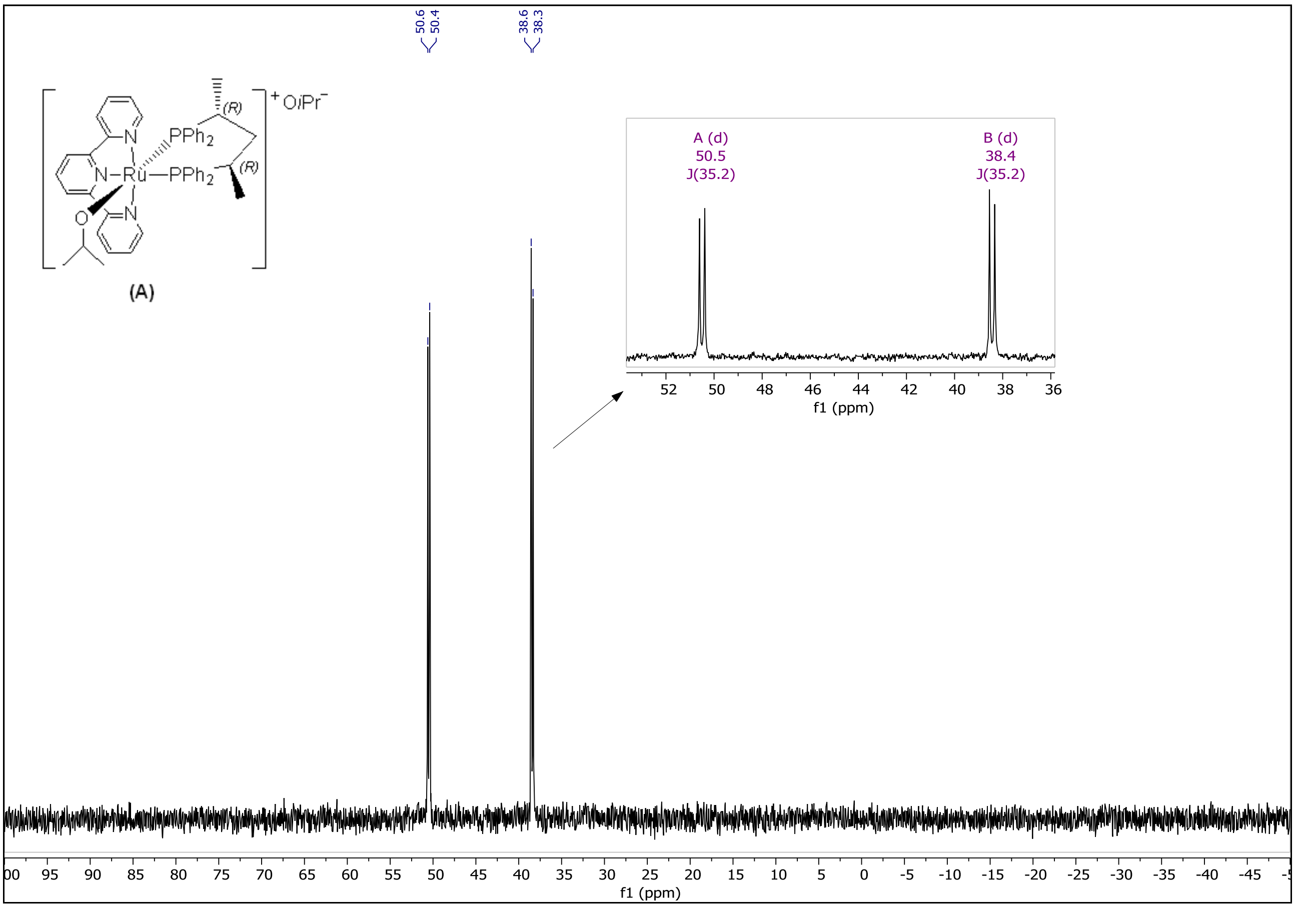

Figure S46. $^{31}P\{^{1}H\}$ NMR spectrum (162.0 MHz) of [Ru(O*i*Pr)((*R*,*R*)-Skewphos)(tpy)](O*i*Pr) (**A**) in 2-propanol-d^8 at 25 °C, obtained after 1 h of visible light irradiation from complex **2** in presence of NaO*i*Pr (3 equiv).

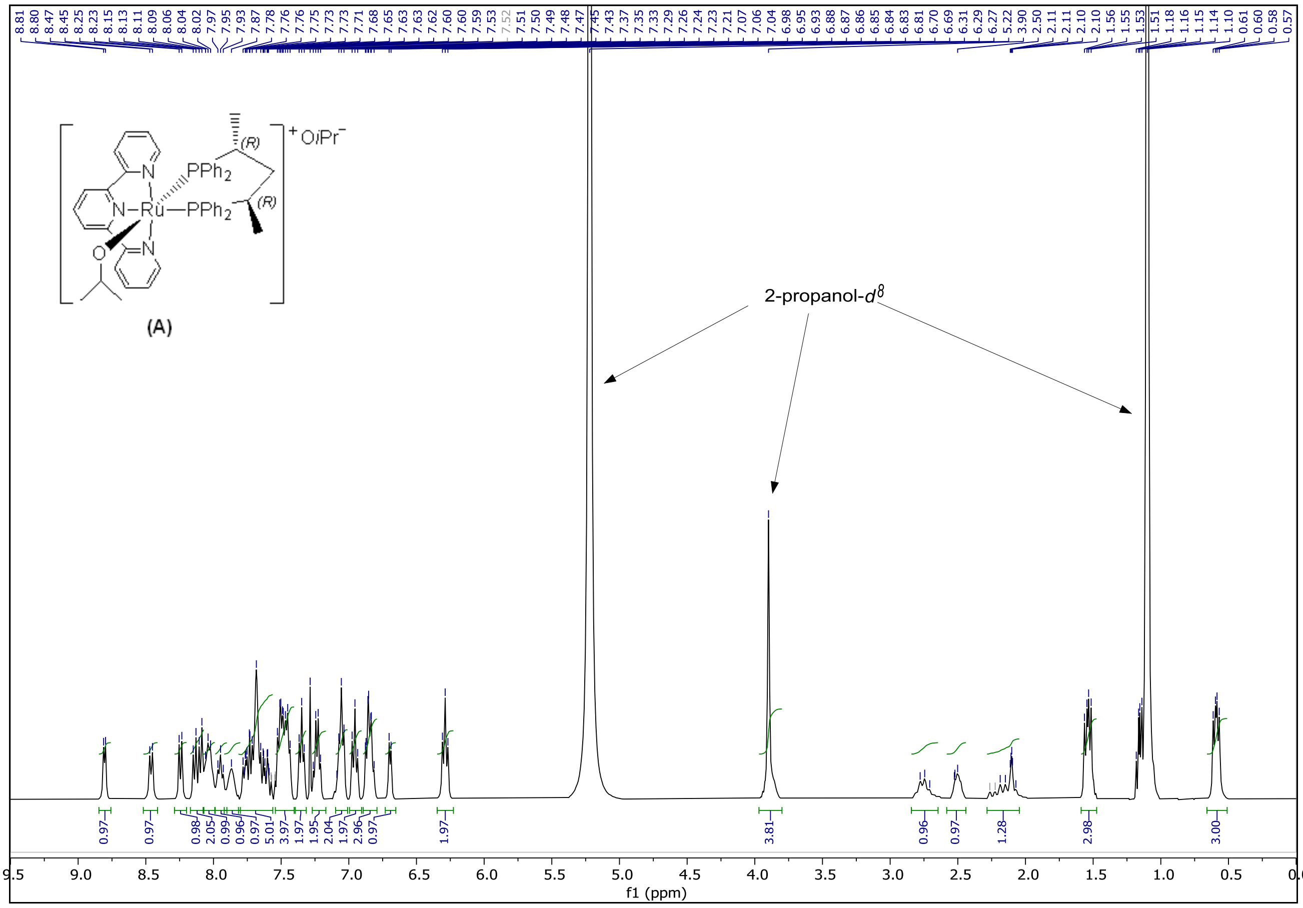

Figure S47. ^{1}H NMR spectrum (400.1 MHz) of [Ru(O*i*Pr)((*R*,*R*)-Skewphos)(tpy)](O*i*Pr) (**A**) in 2-propanol-*d*^{8} at 25 °C, obtained after 1 h of visible light irradiation from complex **2** in presence of NaO*i*Pr (3 equiv).

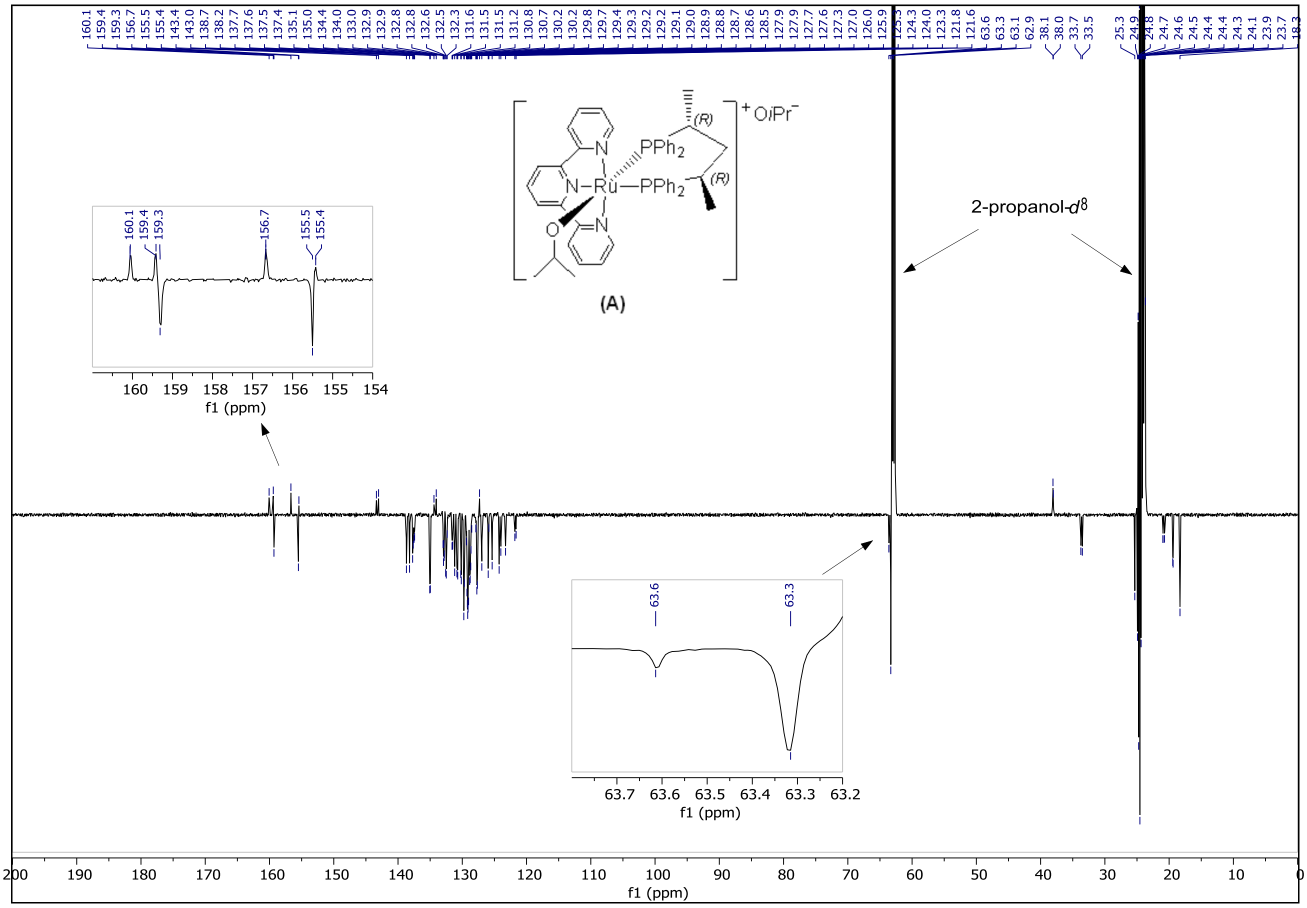

Figure S48. $^{13}C\{^1H\}$ DEPTQ NMR spectrum (100.6 MHz) of [Ru(O*i*Pr)((*R*,*R*)-Skewphos)(tpy)](O*i*Pr) (**A**) in 2-propanol-d^8 at 25 °C, obtained after 1 h of visible light irradiation from complex **2** in presence of NaO*i*Pr (3 equiv).

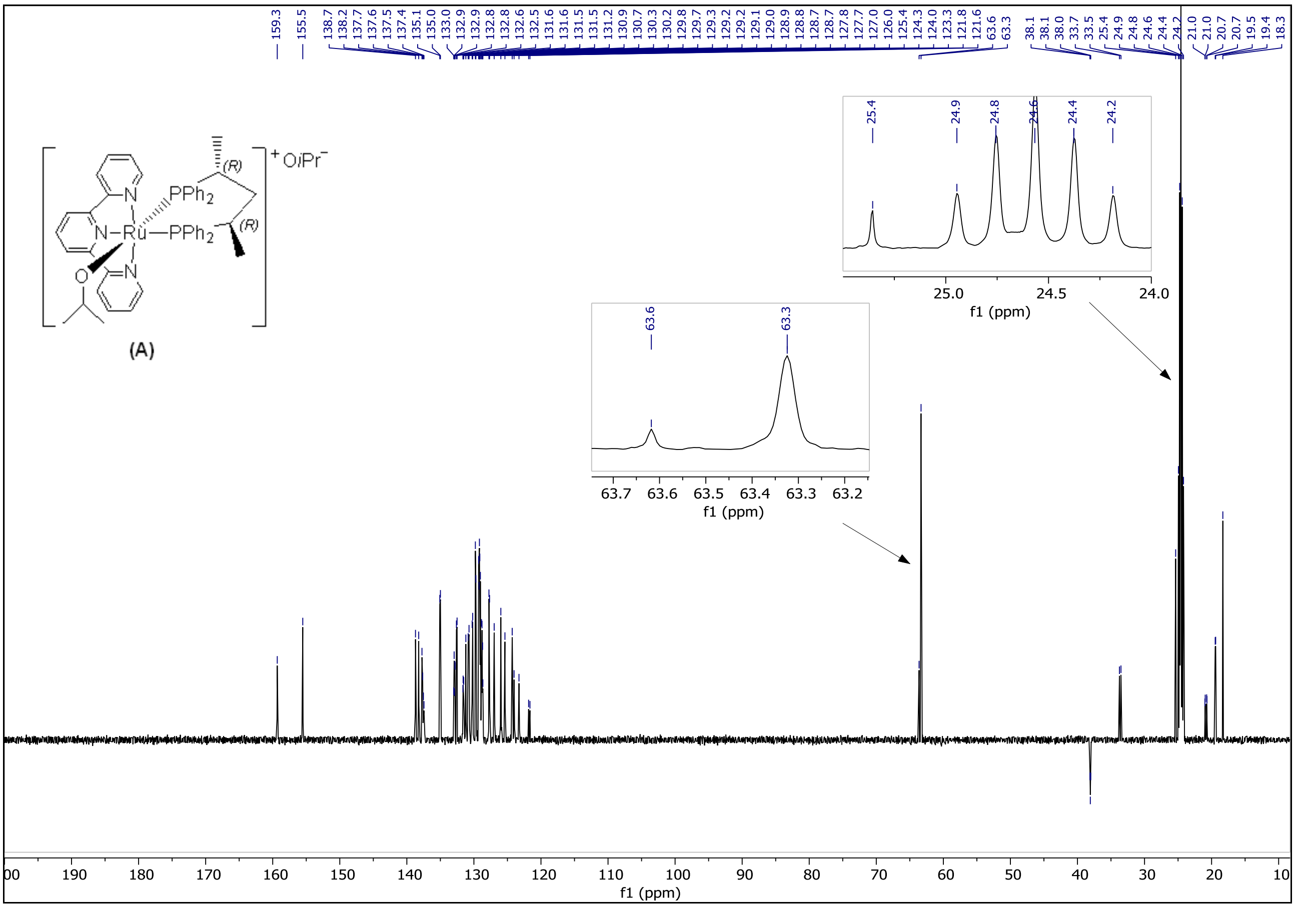

Figure S49. $^{13}C\{^{1}H\}$ DEPT135 NMR spectrum (100.6 MHz) of [Ru(O*i*Pr)((*R*,*R*)-Skewphos)(tpy)](O*i*Pr) (**A**) in 2-propanol-d^8 at 25 °C, obtained after 1 h of visible light irradiation from complex **2** in presence of NaO*i*Pr (3 equiv).

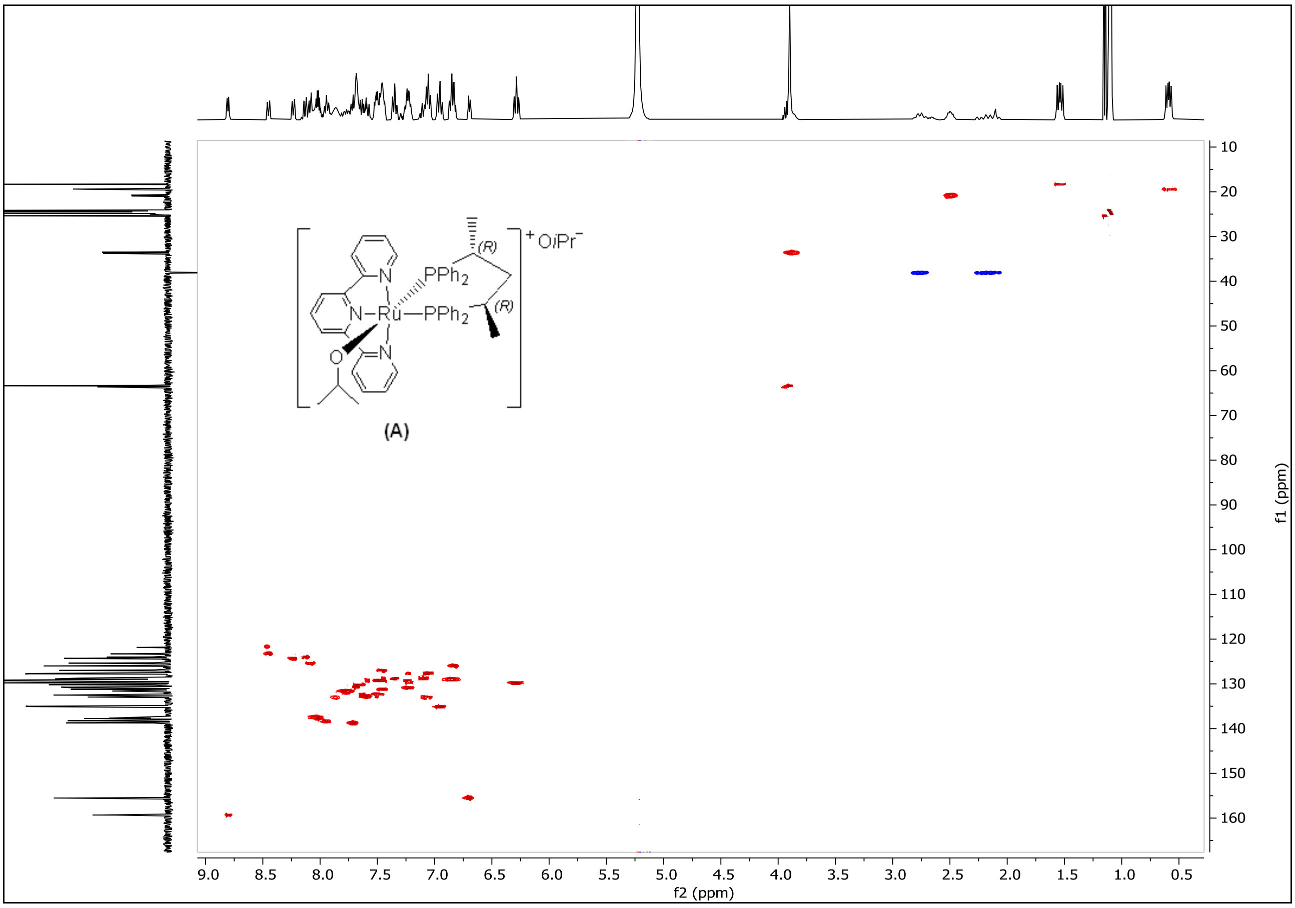

Figure S50. ^{1}H-^{13}C HSQC 2D NMR spectrum of [Ru(O*i*Pr)((*R*,*R*)-Skewphos)(tpy)](O*i*Pr) (**A**) in 2-propanol-d^{8} at 25 °C, obtained after 1 h of visible light irradiation from complex **2** in presence of NaO*i*Pr (3 equiv).

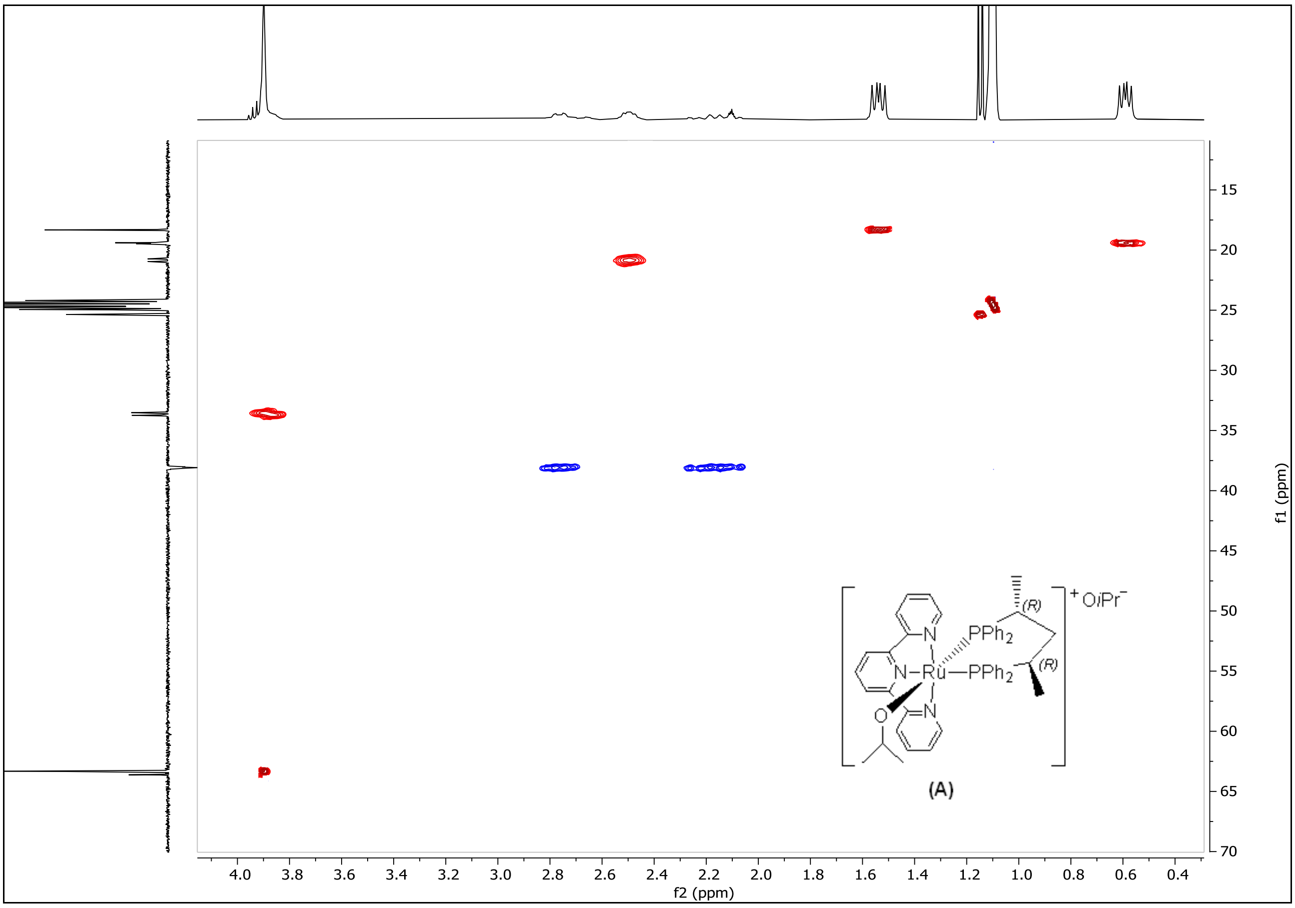

Figure S51. Aliphatic region of the ^{1}H-^{13}C HSQC 2D NMR spectrum of [Ru(O*i*Pr)((*R*,*R*)-Skewphos)(tpy)](O*i*Pr) (**A**) in 2-propanol-d^{8} at 25 °C, obtained after 1 h of visible light irradiation from complex **2** in presence of NaO*i*Pr (3 equiv).

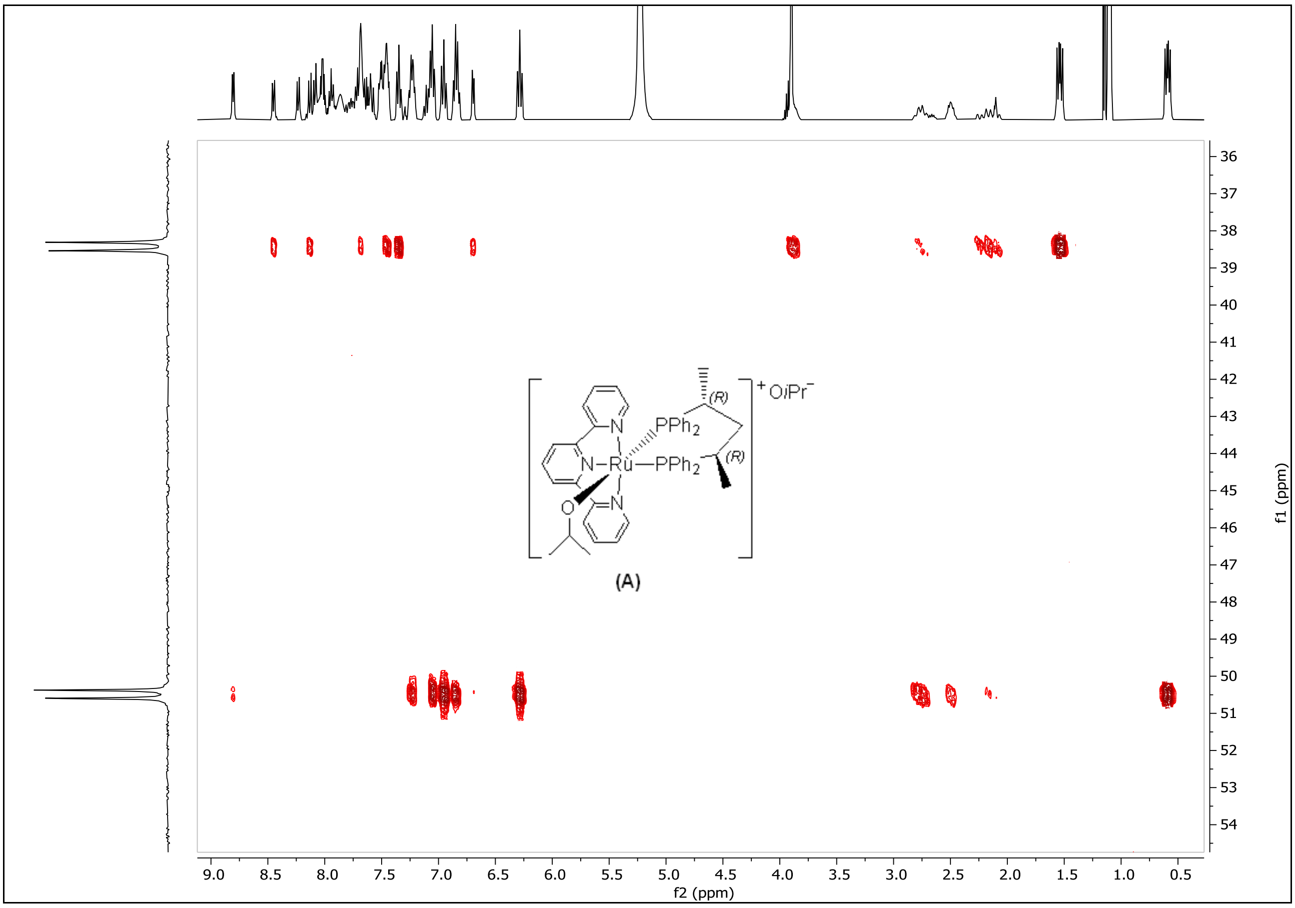

Figure S52. ^{1}H-^{31}P HMBC 2D NMR spectrum of [Ru(O*i*Pr)((*R*,*R*)-Skewphos)(tpy)](O*i*Pr) (**A**) in 2-propanol-*d*^{8} at 25 °C, obtained after 1 h of visible light irradiation from complex **2** in presence of NaO*i*Pr (3 equiv).

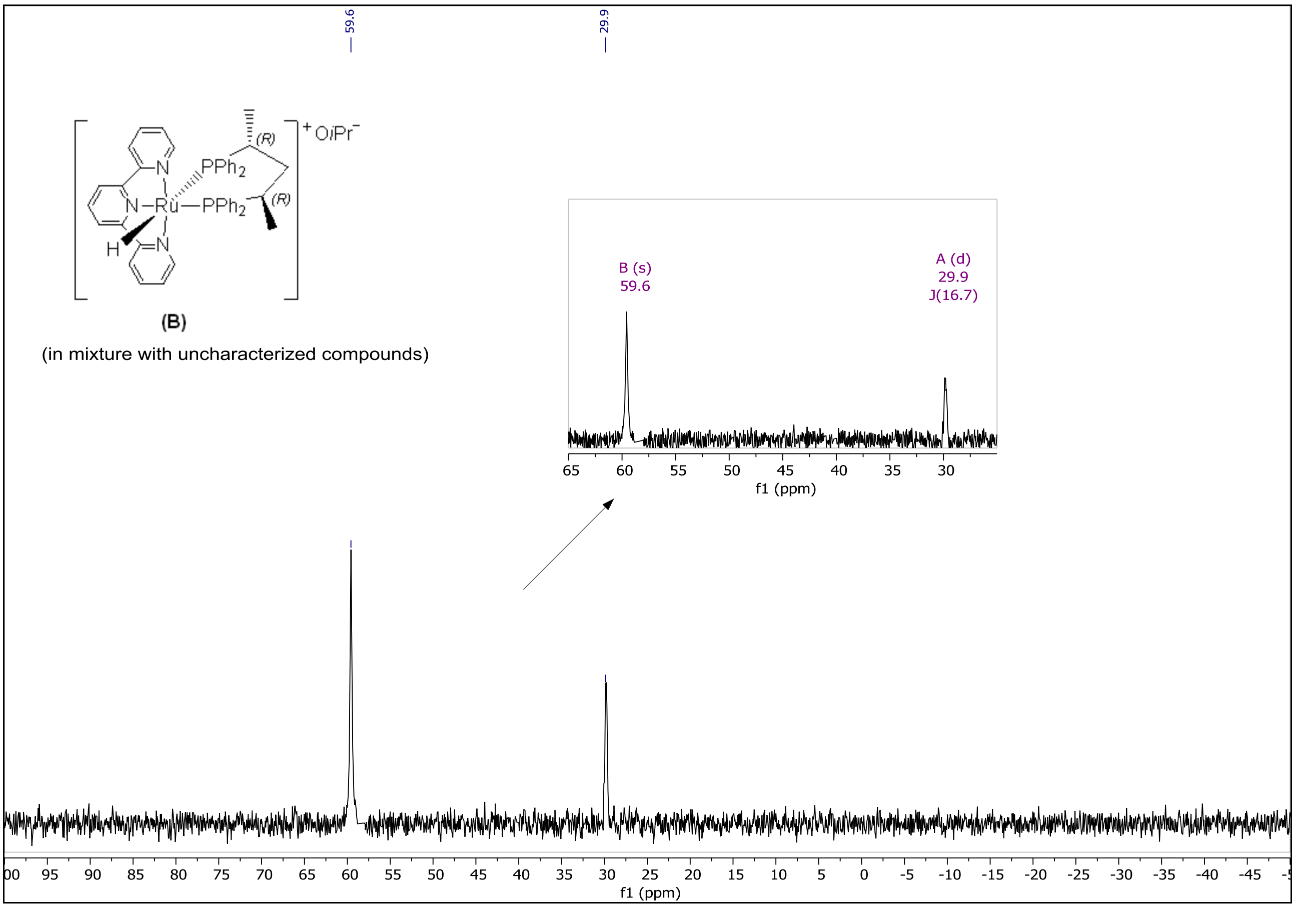

Figure S53. $^{31}P\{^{1}H\}$ NMR spectrum (162.0 MHz) with evidences of the formation of [RuH((*R*,*R*)-Skewphos)(tpy)](O*i*Pr) (**B**) in 2-propanol-d^8 at 25 °C (in mixture with uncharacterized compounds), obtained after 2 h of visible light irradiation from complex **2** in presence of NaO*i*Pr (3 equiv).

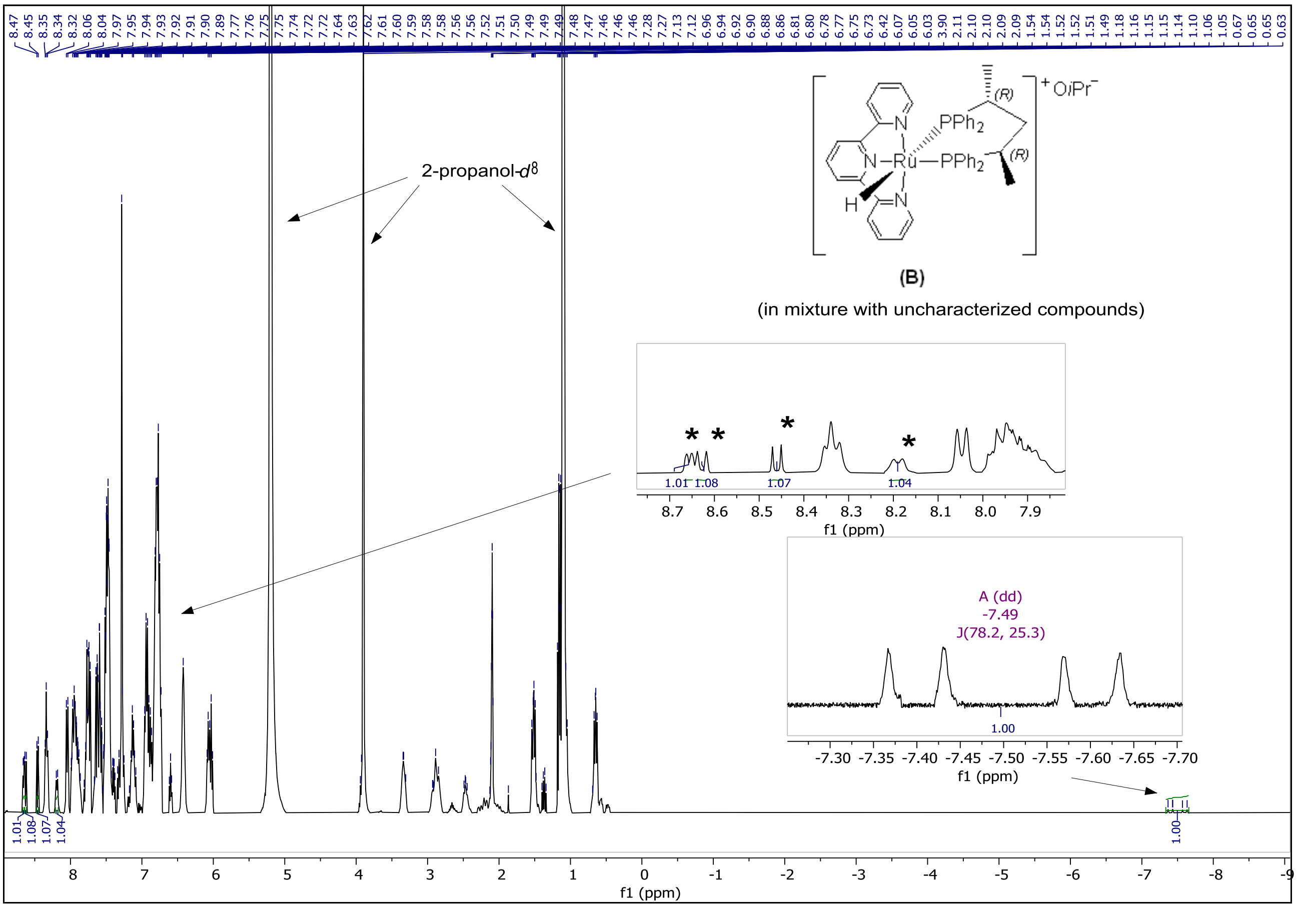

Figure S54. ^{1}H NMR spectrum (400.1 MHz) with evidences of the formation of [RuH((*R*,*R*)-Skewphos)(tpy)](O*i*Pr) (**B**) in 2-propanol-*d*^{8} at 25 °C (in mixture with uncharacterized compounds), obtained after 2 h of visible light irradiation from complex **2** in presence of NaO*i*Pr (3 equiv). The asterisk (*) indicates tpy proton peaks belonging to the Ru(II) hydride species.

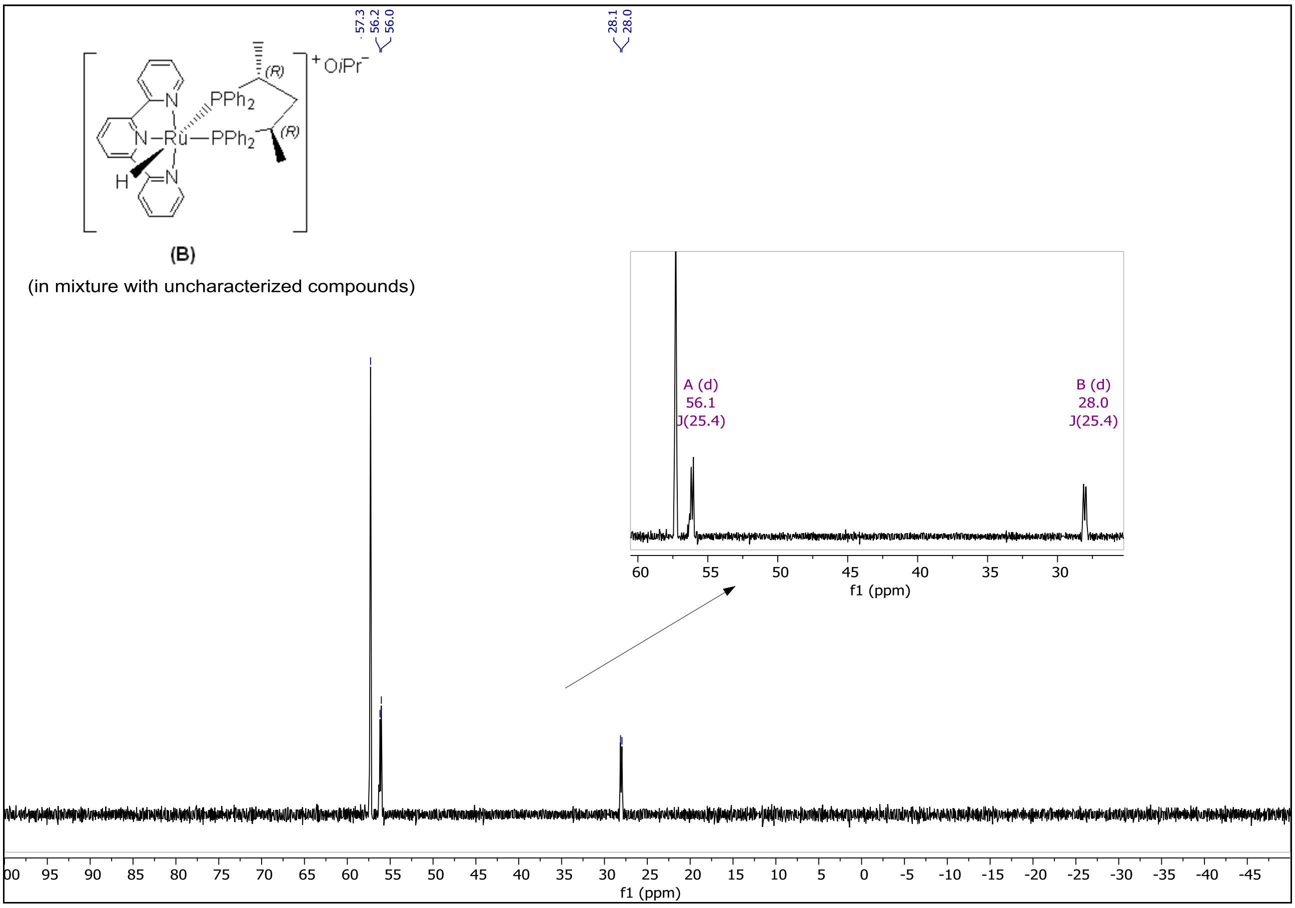

Figure S55. $^{31}P\{^{1}H\}$ NMR spectrum (162.0 MHz) with evidences of the formation of [RuH((*R*,*R*)-Skewphos)(tpy)](O*i*Pr) (**B**) in 2-propanol/toluene-d^8 1:1 (v/v) at 25 °C (in mixture with uncharacterized compounds), obtained after 2 h of visible light irradiation from complex **2** in presence of NaO*i*Pr (3 equiv).

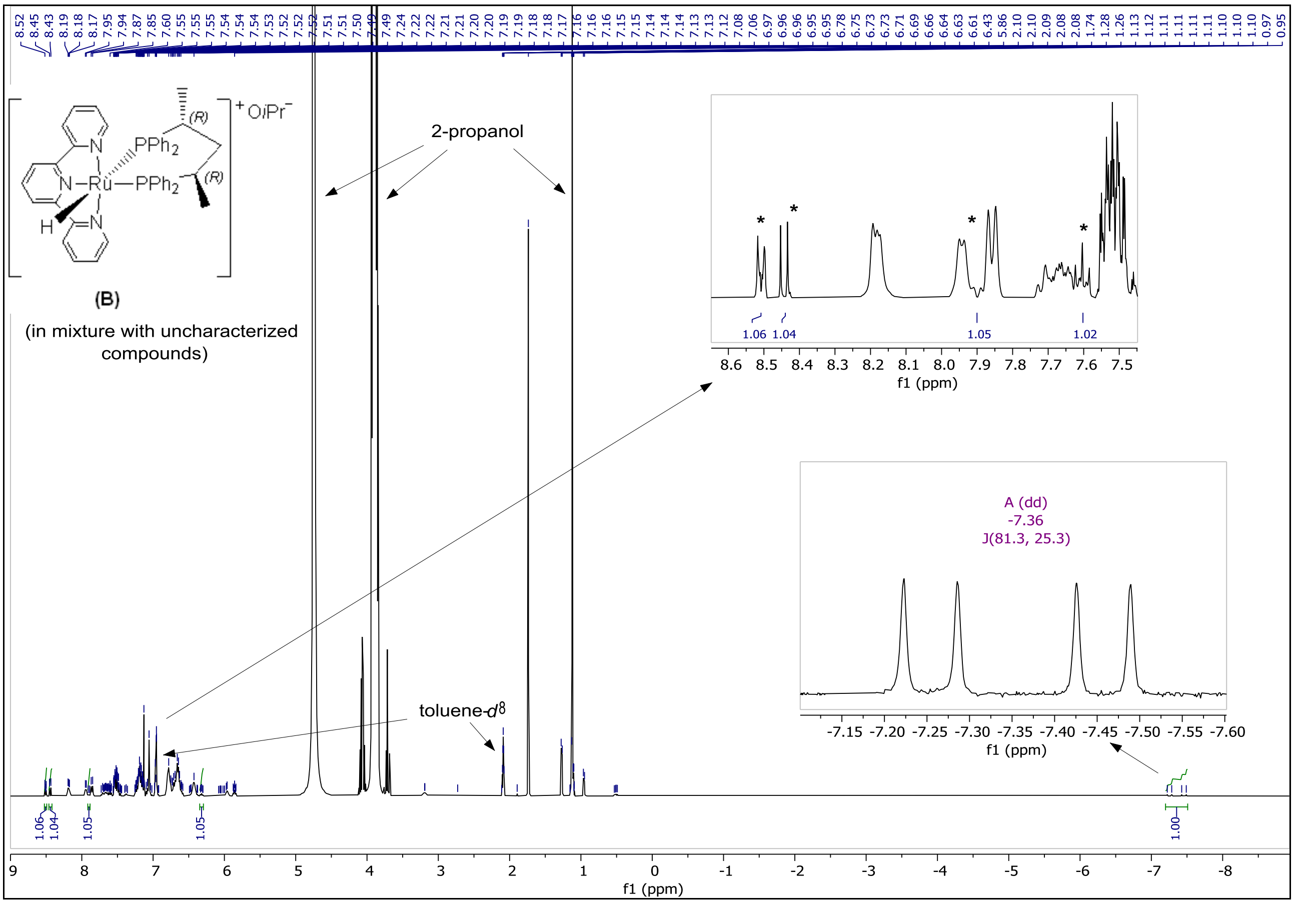

Figure S56. ^{1}H NMR spectrum (400.1 MHz) with evidences of the formation of [RuH((*R*,*R*)-Skewphos)(tpy)](O*i*Pr) (**B**) in 2-propanol/toluene-d^8 1:1 (v/v) at 25 °C (in mixture with uncharacterized compounds), obtained after 2 h of visible light irradiation from complex **2** in presence of NaO*i*Pr (3 equiv). The asterisk (*) indicates tpy proton peaks belonging to the Ru(II) hydride species. The methyl signal of 2-propanol at δ 1.14 ppm was presaturated.

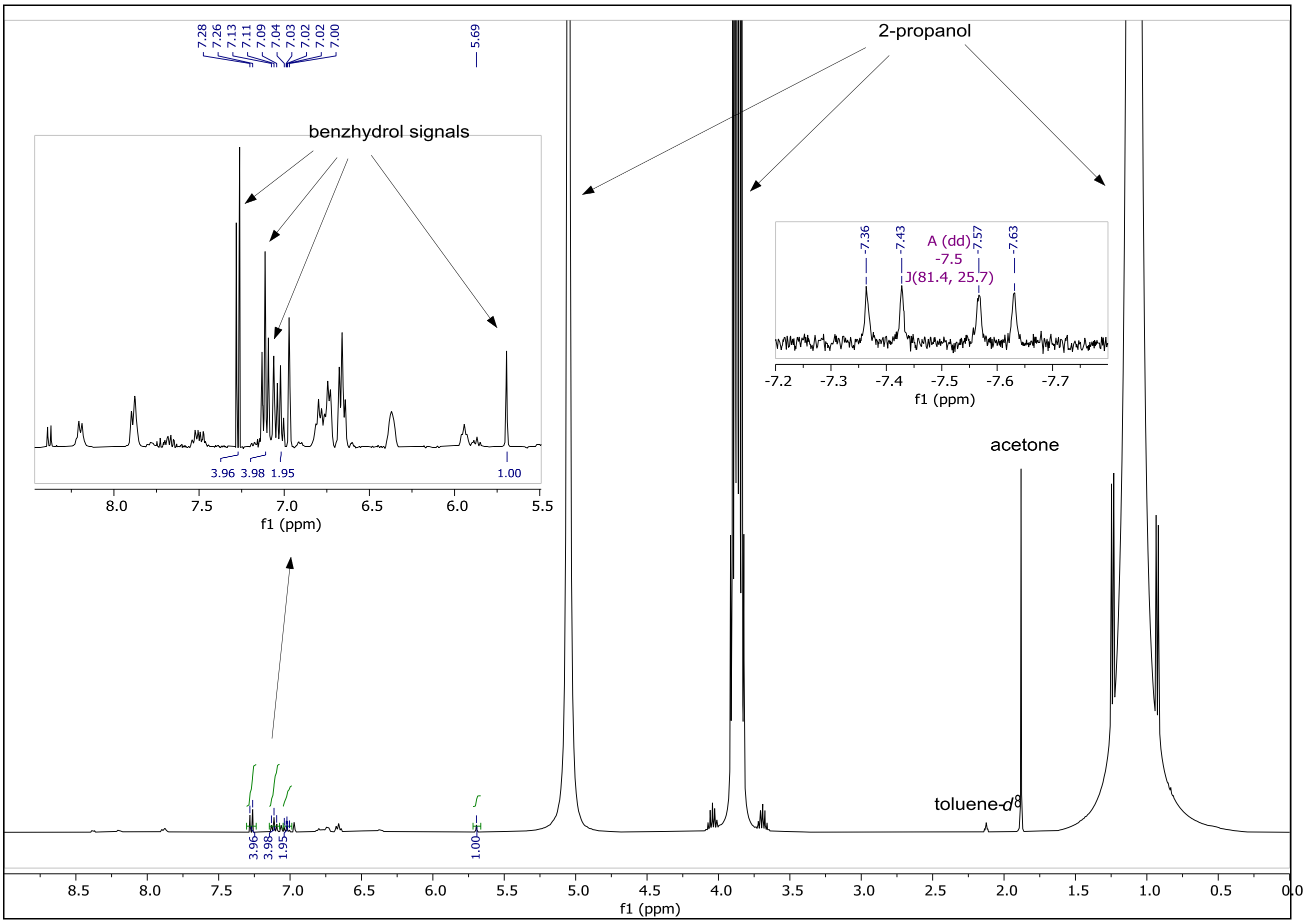

Figure S57. ^{1}H NMR spectrum (400.1 MHz) with evidences of the reduction of benzophenone to benzhydrol promoted by the mixture containing [RuH((*R*,*R*)-Skewphos)(tpy)](O*i*Pr) (**B**) in 2-propanol/toluene-*d*[8] 1:1 (v/v) at 25 °C, obtained after 1 h of visible light irradiation.

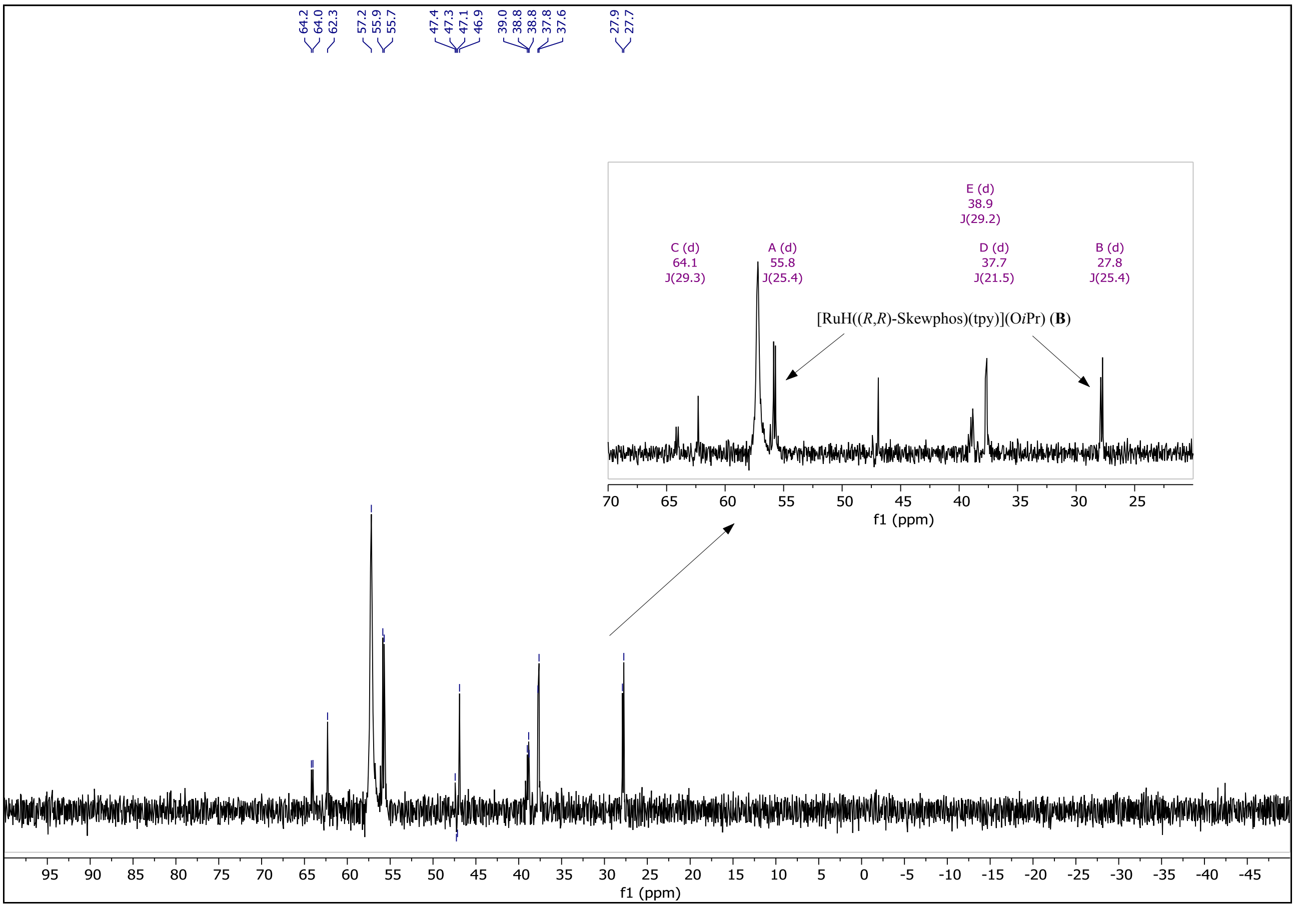

Figure S58. $^{31}P\{^{1}H\}$ NMR spectrum (162.0 MHz) obtained after the addition of benzophenone (1 equiv) to the mixture containing [RuH((*R*,*R*)-Skewphos)(tpy)](O*i*Pr) (**B**) in 2-propanol/toluene-d^8 1:1 (v/v) at 25 °C, and 1 h of visible light irradiation.

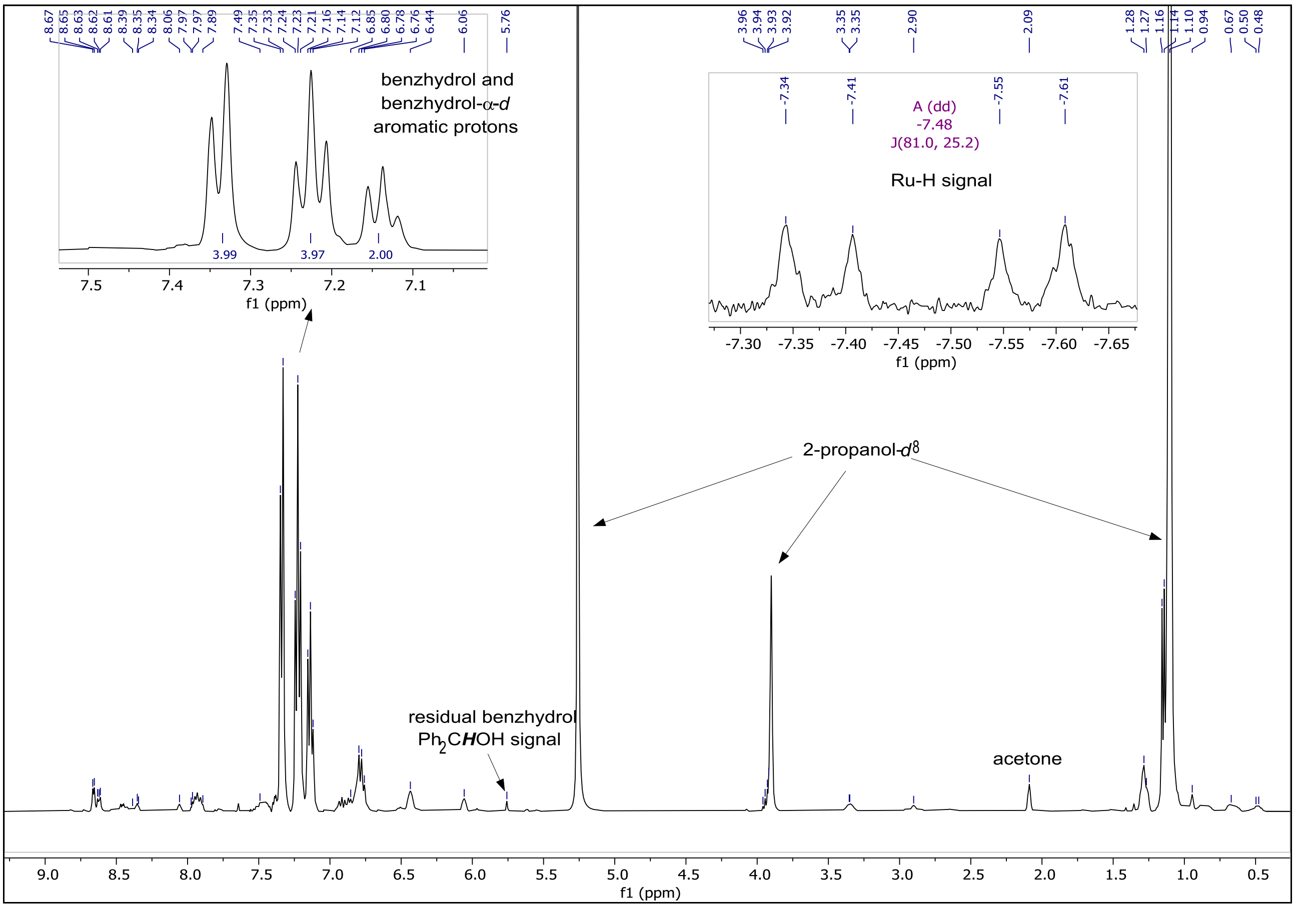

Figure S59. ^{1}H NMR spectrum (400.1 MHz) with evidences of the reduction of benzophenone to benzhydrol promoted by the mixture containing [RuH((*R*,*R*)-Skewphos)(tpy)](O*i*Pr) (**B**) in 2-propanol-*d*[8] at 25 °C, obtained after 1 h of visible light irradiation.

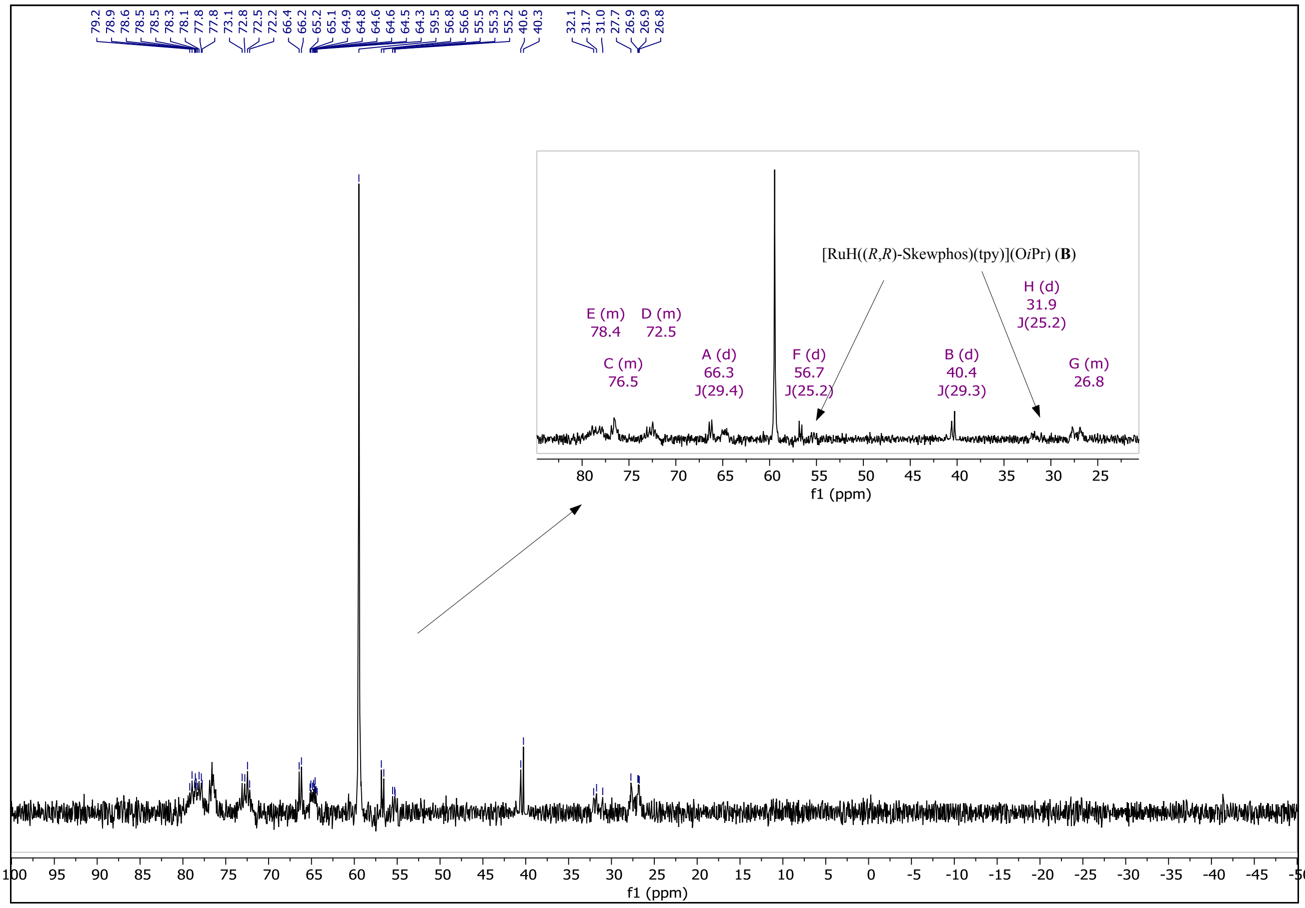

Figure S60. $^{31}P\{^1H\}$ NMR spectrum (162.0 MHz) obtained after the addition of benzophenone (1 equiv) to the mixture containing [RuH((*R*,*R*)-Skewphos)(tpy)](O*i*Pr) (**B**) in 2-propanol-d^8 at 25 °C, and 1 h of visible light irradiation.

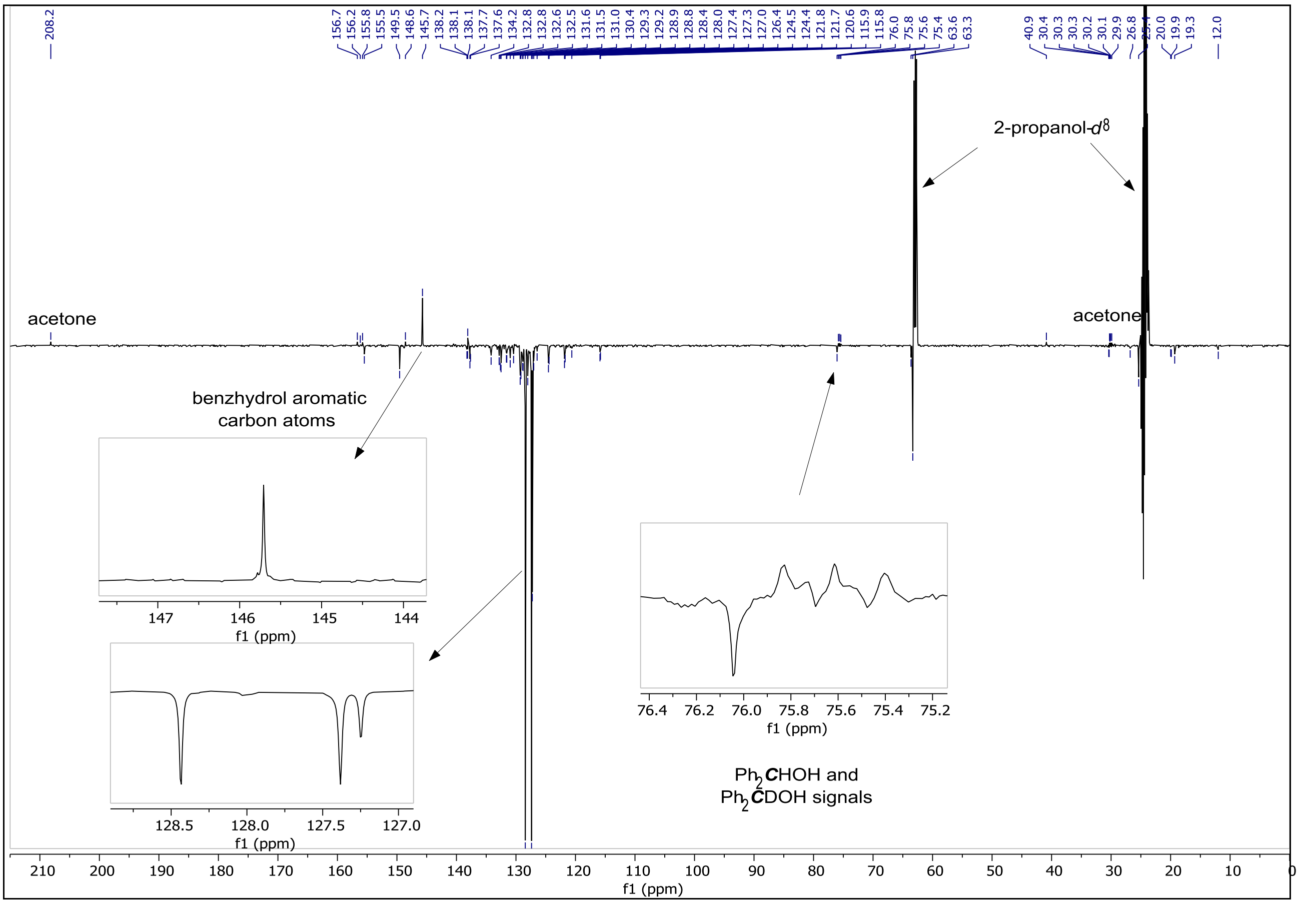

Figure S61. $^{13}C\{^{1}H\}$ DEPTQ NMR spectrum (100.6 MHz) with evidences of the reduction of benzophenone to benzhydrol promoted by the mixture containing [RuH((*R*,*R*)-Skewphos)(tpy)](O*i*Pr) (**B**) in 2-propanol-*d*^{8} at 25 °C, obtained after 1 h of visible light irradiation.

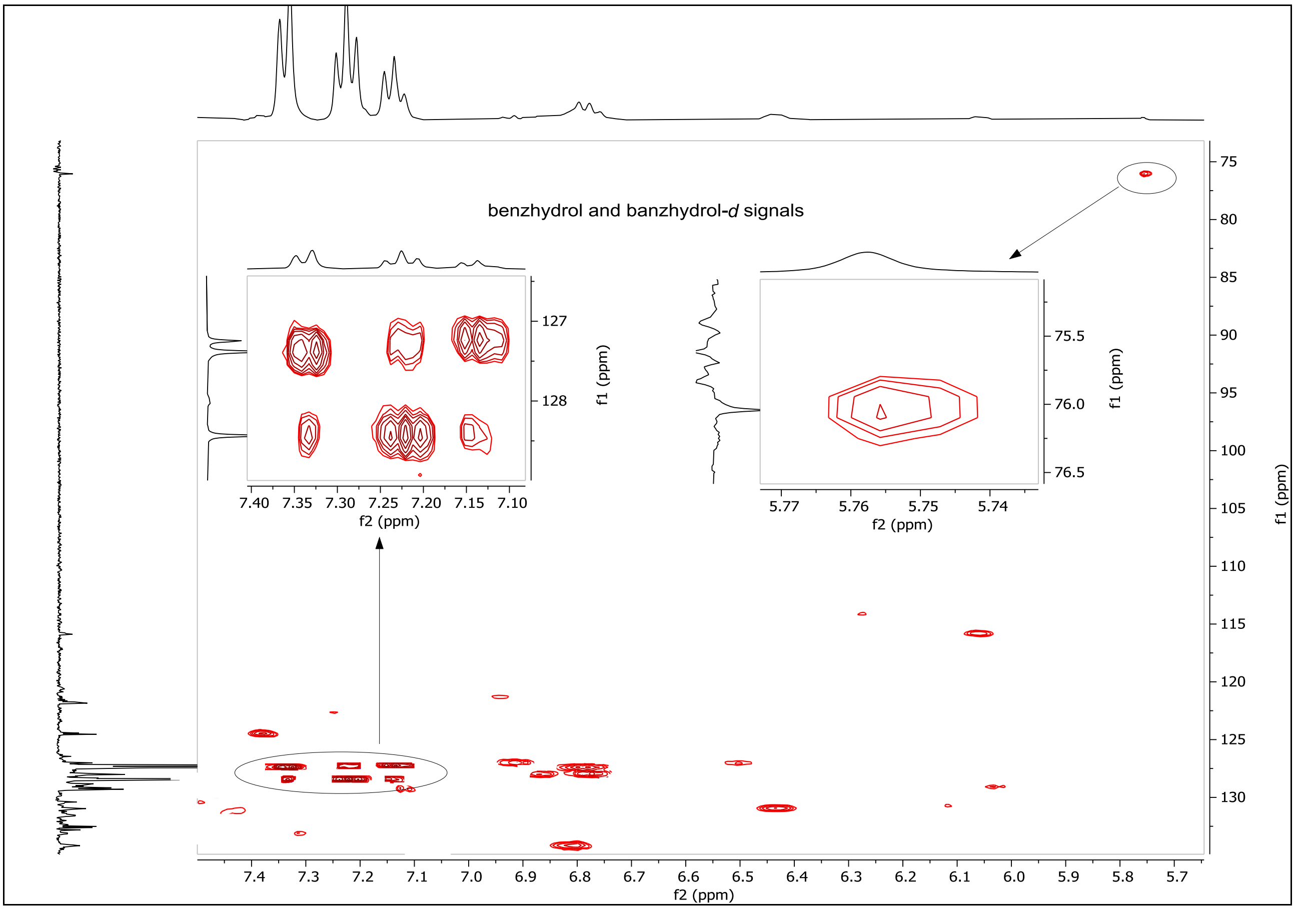

Figure S62. Aromatic region of the ^{1}H-^{13}C HSQC 2D NMR spectrum with evidences of the reduction of benzophenone to benzhydrol promoted by the mixture containing [RuH((*R*,*R*)-Skewohos)(tpy)](O*i*Pr) (**B**) in 2-propanol-d^{8} at 25 °C, obtained after 1 h of visible light irradiation.

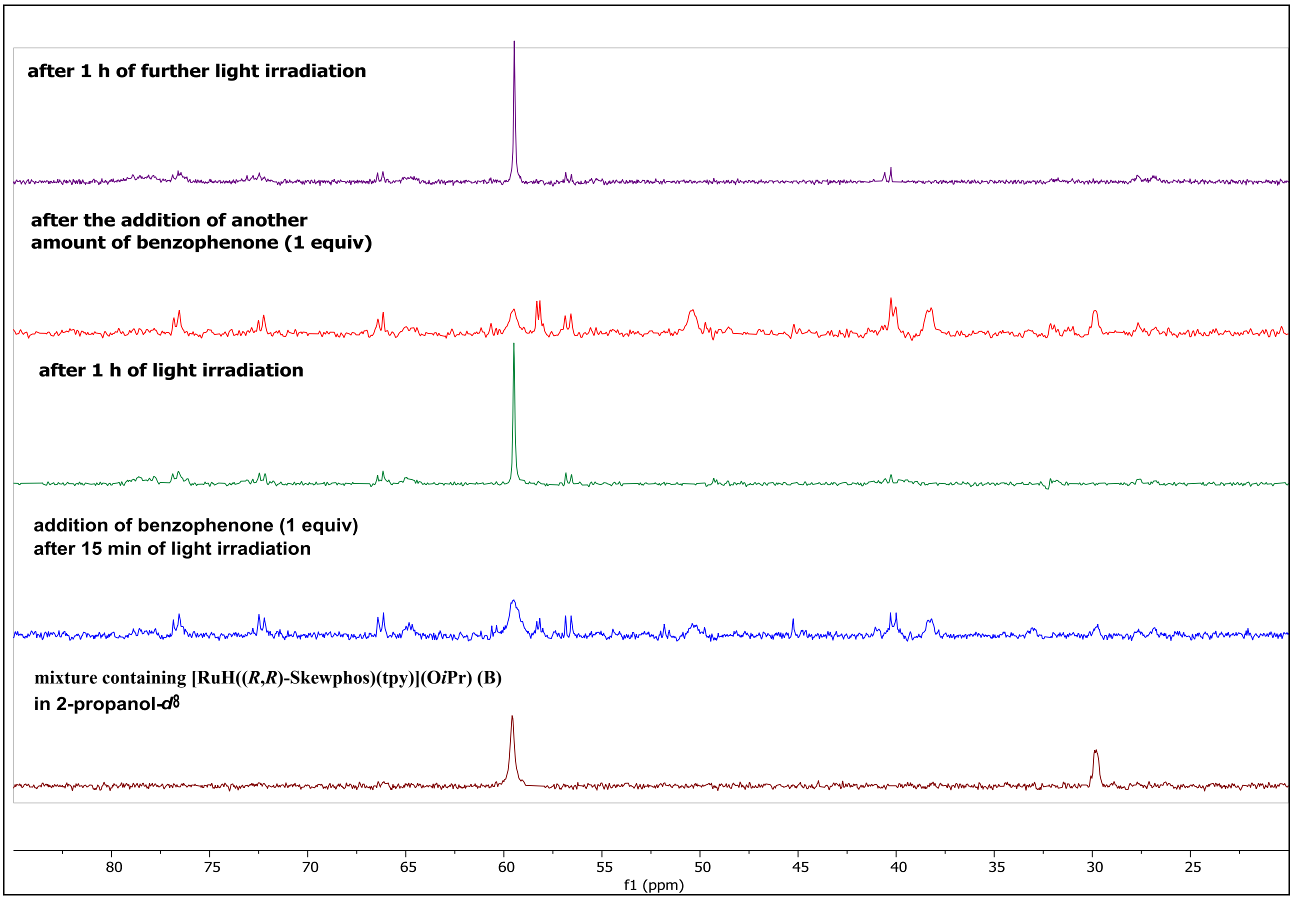

Figure S63. Effect of the addition of benzophenone (1-2 equiv) to the mixture containing [RuH((*R*,*R*)-Skewphos)(tpy)](O*i*Pr) (**B**) and after visible light irradiation in the $^{31}P\{^{1}H\}$ NMR spectrum (162.0 MHz) in 2-propanol-d^8 at 25 °C.

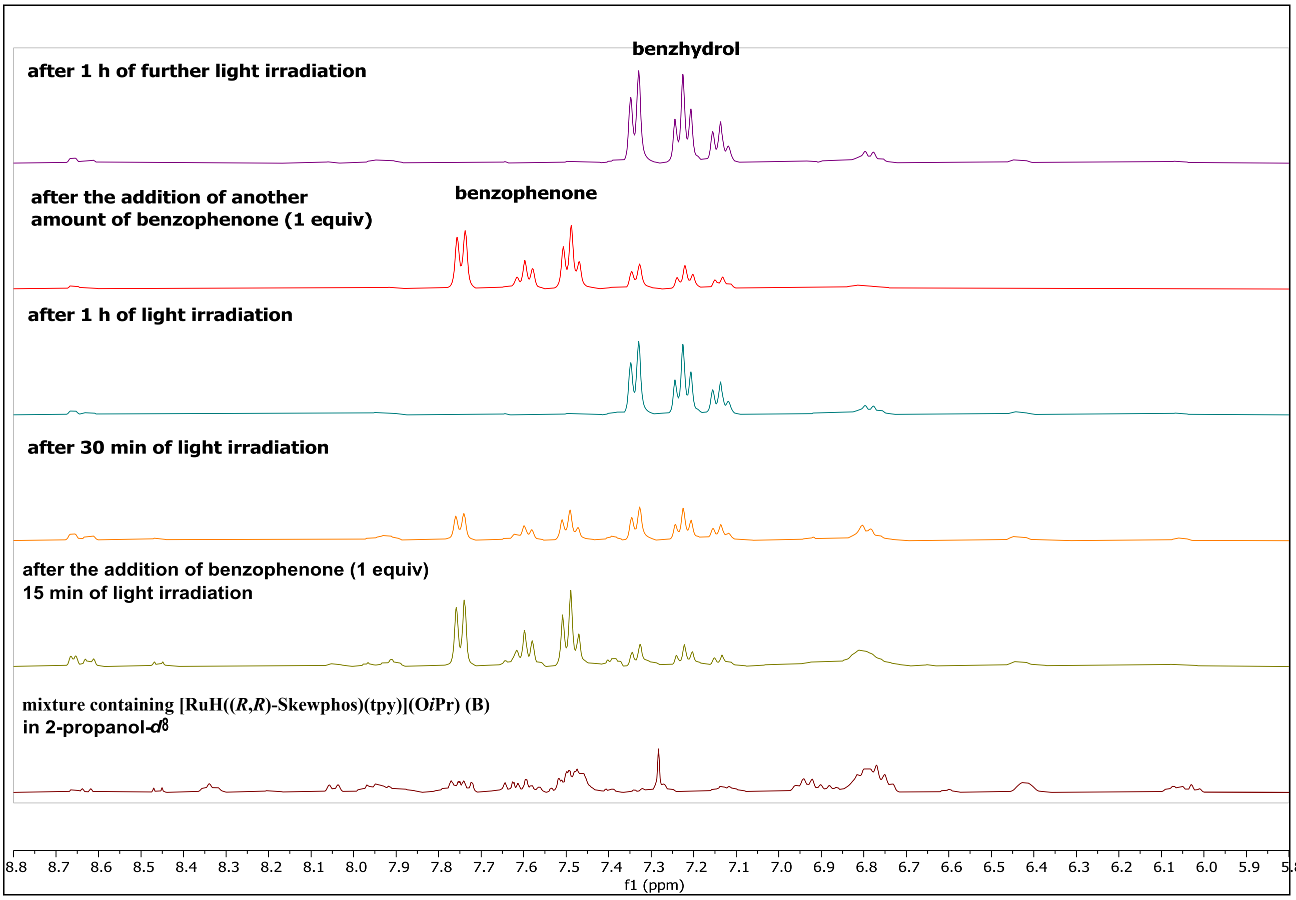

Figure S64. Effect of the addition of benzophenone (1-2 equiv) to the mixture containing [RuH((*R*,*R*)-Skewphos)(tpy)](O*i*Pr) (**B**) and after visible light irradiation in the aromatic region of the ^{1}H NMR spectrum (400.1 MHz) in 2-propanol-d^{8} at 25 °C.

Single Crystal X-Ray Structure Determination of Compounds 3a (CCDC 2165467) and 4a (CCDC 2165468)

General Data

Data were collected on a Bruker D8 Venture single crystal x-ray diffractometer equipped with a CPAD detector (Bruker Photon II), an IMS micro source with MoK_α radiation (λ = 0.71073 Å), and a Helios optic or a CMOS detector (Bruker Photon-100), a TXS rotating anode with MoK_α radiation, and a Helios optic using the APEX3 software package.[1] Measurements were performed on single crystals coated with perfluorinated ether. The crystals were fixed on top of a kapton micro sampler and frozen under a stream of cold nitrogen. A matrix scan was used to determine the initial lattice parameters. Reflections were corrected for Lorentz and polarisation effects, scan speed, and background using SAINT.[2] Absorption correction, including odd and even ordered spherical harmonics was performed using SADABS.[2] Space group assignments were based upon systematic absences, E statistics, and successful refinement of the structures. The structures were solved using SHELXT with the aid of successive difference Fourier maps, and were refined against all data using SHELXL in conjunction with SHELXLE.[3,4,5] Hydrogen atoms were calculated in ideal positions as follows: Methyl hydrogen atoms were refined as part of rigid rotating groups, with a C–H distance of 0.98 Å and $U_{iso(H)} = 1.5 \cdot U_{eq(C)}$. Non-methyl hydrogen atoms were placed in calculated positions and refined using a riding model, with methylene and aromatic C–H distances of 0.99 Å and 0.95 Å, respectively, and other C–H distances of 1.00 Å, all with $U_{iso(H)} = 1.2 \cdot U_{eq(C)}$. Non-hydrogen atoms were refined with anisotropic displacement parameters. Full-matrix least-squares refinements were carried out by minimizing $\Sigma w(F_o^2 - F_c^2)^2$ with the SHELXL weighting scheme.[3] Neutral atom scattering factors for all atoms and anomalous dispersion corrections for the non-hydrogen atoms were taken from *International Tables for Crystallography*.[6] The unit cell of **4a** contains several mixed and disordered solvent molecules which were treated as a diffuse contribution to the overall scattering without specific atom positions using the PLATON/SQUEEZE procedure.[7] Images of the crystal structures were generated with Mercury.[8] CCDC 2165467-2165468 contain the supplementary crystallographic data for this paper. These data are provided free of charge by The Cambridge Crystallographic Data Centre.

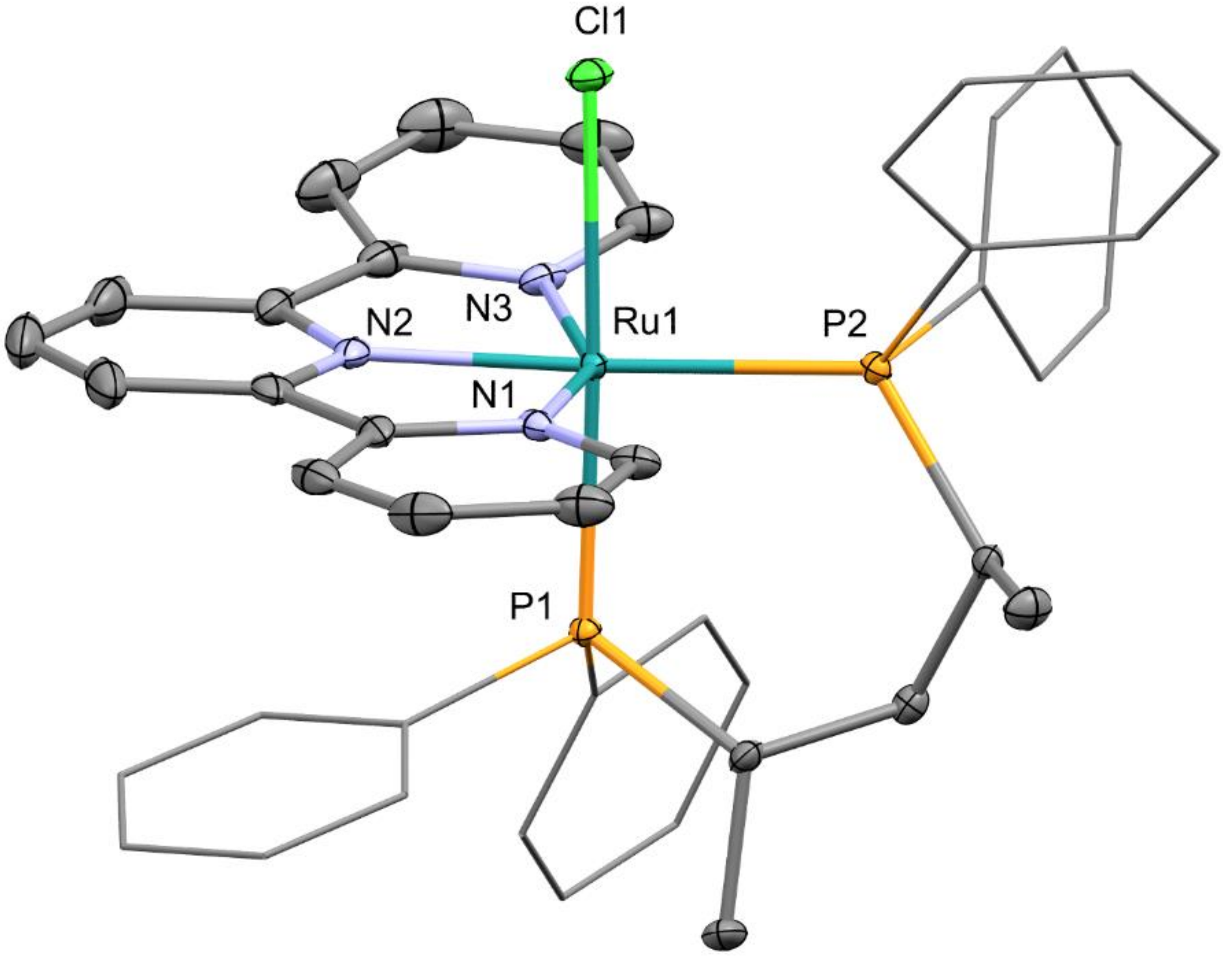

Figure S65. ORTEP style plot of compound **3a** in the solid state (CCDC 2165467). Ellipsoids are drawn at the 50% probability level. Hydrogen atoms, co-crystallized solvent molecules, and the PF_6 counterion are omitted, and phenyl groups are simplified as wireframes for clarity. Selected bond lengths [Å] and angles [°]: Ru1–N1 2.107(2), Ru1–N2 2.020(2), Ru1–N3 2.148(2), Ru1–P1 2.2893(8), Ru1–P2 2.3745(8), Ru1–Cl1 2.4804(7), N2–Ru1–N1 78.20(10), N2–Ru1–N3 77.21(11), N1–Ru1–N3 154.97(10), N2–Ru1–P1 91.14(7), N1–Ru1–P1 88.90(7), N3–Ru1–P1 96.21(7), N2–Ru1–P2 175.52(8), N1–Ru1–P2 98.76(7), N3–Ru1–P2 105.49(7), P2–Ru1–P1 92.11(3), N2–Ru1–Cl1 86.44(7), N1–Ru1–Cl1 92.93(7), N3–Ru1–Cl1 80.93(7), P1–Ru1–Cl1 176.60(3), P2–Ru1–Cl1 90.44(3).

Single Crystal X-Ray Structure Determination of Compound 3a (CCDC 2165467).

Detailed Crystallographic Data.

Diffractometer operator:	C. Jandl
Scanspeed	1-10 s per frame
dx	40 mm
Frames:	2045 measured in 11 XYZ data sets
phi-scans with delta phi	0.5
omega-scans with delta omega	0.5
shutterless mode	

Crystal Data:

$[C_{44}H_{41}ClN_3P_2Ru]PF_6 \cdot 2(CH_3OH)$	D_x = 1.545 Mg m^{-3}
M_r = 1019.31	Melting point: ? K
Orthorhombic, $P2_12_12_1$	Mo *K*α radiation, λ = 0.71073 Å
Hall symbol: P 2ac 2ab	Cell parameters from 9580 reflections
a = 14.4507 (15) Å	θ = 2.8–26.8°
b = 17.0565 (17) Å	μ = 0.60 mm^{-1}
c = 17.7838 (18) Å	*T* = 100 K
V = 4383.3 (8) $Å^3$	Fragment, red-orange
Z = 4	0.17 × 0.12 × 0.12 mm
F(000) = 2088	

Data collection:

Bruker D8 Venture diffractometer	8960 independent reflections
Radiation source: TXS rotating anode	8681 reflections with $I > 2\sigma(I)$
Helios optic monochromator	R_{int} = 0.046

Detector resolution: 16 pixels mm^{-1}	θ_{max} = 26.4°, θ_{min} = 2.4°
phi– and ω–rotation scans	h = −18 18
Absorption correction: multi-scan *SADABS* 2016/2, Bruker	k = −21 21
T_{min} = 0.681, T_{max} = 0.745	l = −22 22
88154 measured reflections	

Data refinement:

Refinement on F^2	Hydrogen site location: inferred from neighbouring sites
Least-squares matrix: full	H-atom parameters constrained
$R[F^2 > 2\sigma(F^2)]$ = 0.023	W = $1/[\Sigma^2(FO^2) + (0.0288P)^2 + 3.2036P]$ WHERE $P = (FO^2 + 2FC^2)/3$
$wR(F^2)$ = 0.058	$(\Delta/\sigma)_{max}$ = 0.001
S = 1.06	$\Delta\rho_{max}$ = 0.88 e Å^{-3}
8960 reflections	$\Delta\rho_{min}$ = −0.63 e Å^{-3}
565 parameters	Extinction correction: none
0 restraints	Extinction coefficient: -
0 constraints	Absolute structure: Flack, Parsons[9,10]
Primary atom site location: iterative	Absolute structure parameter: 0.008 (6)
Secondary atom site location: difference Fourier map	

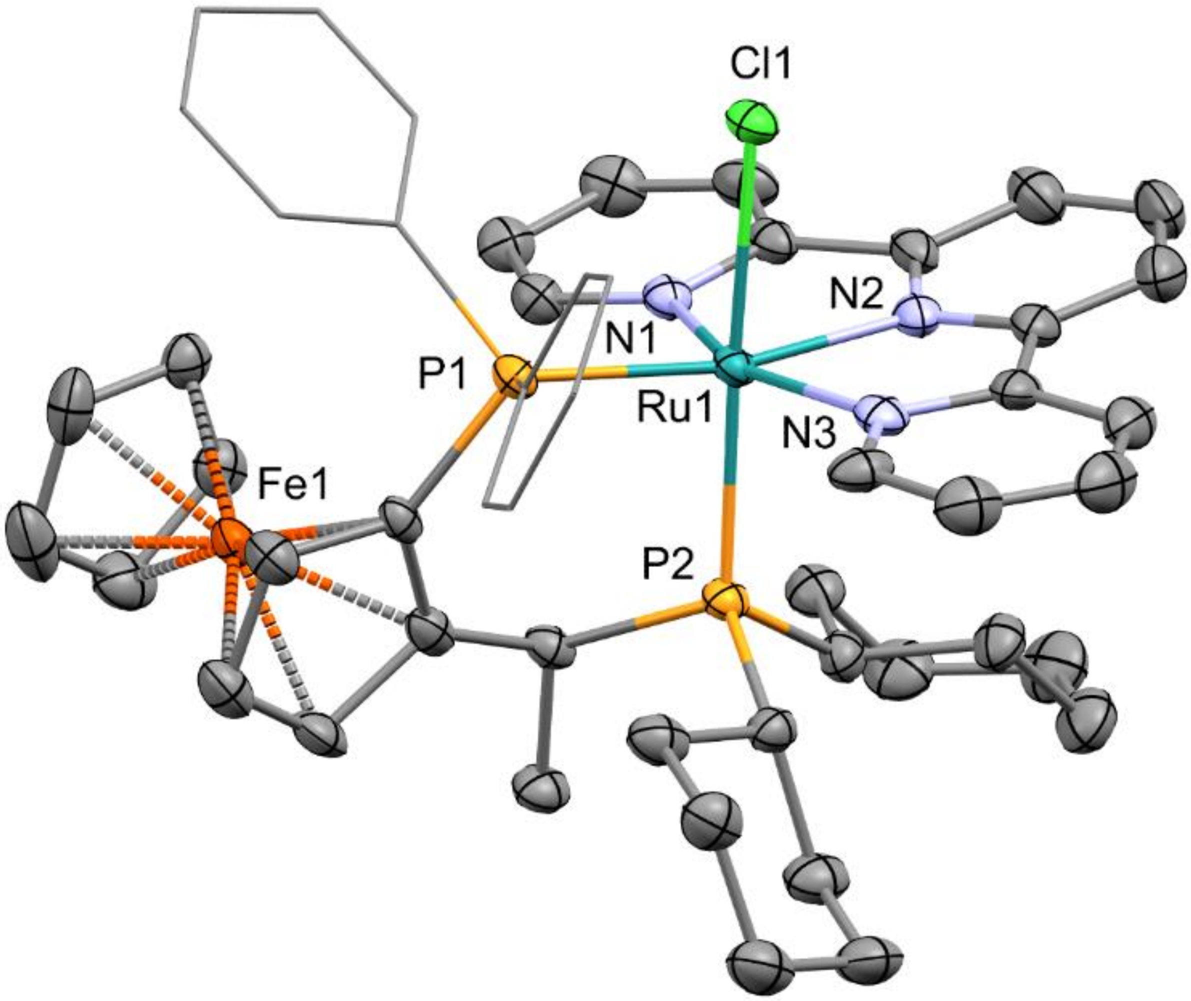

Figure S66. ORTEP style plot of compound **4a** in the solid state (CCDC 2165468). Ellipsoids are drawn at the 50% probability level. Hydrogen atoms, co-crystallized solvent molecules, and the PF_6 counterion are omitted, and phenyl groups are simplified as wireframes for clarity. Selected bond lengths [Å] and angles [°]: Ru1–N1 2.083(3), Ru1–N2 2.009(3), Ru1–N3 2.129(3), Ru1–P1 2.3410(10), Ru1–P2 2.3287(10), Ru1–Cl1 2.4566(9), N2–Ru1–N1 78.57(13), N2–Ru1–N3 77.83(14), N1–Ru1–N3 156.40(13), N2–Ru1–P1 168.56(9), N1–Ru1–P1 98.36(9), N3–Ru1–P1 104.85(10), N2–Ru1–P2 97.83(9), N1–Ru1–P2 91.73(9), N3–Ru1–P2 91.19(9), P1–Ru1–P2 93.25(4), N2–Ru1–Cl1 81.11(9), N1–Ru1–Cl1 90.34(9), N3–Ru1–Cl1 86.32(9), P1–Ru1–Cl1 87.93(3), P2–Ru1–Cl1 177.45(4).

Single Crystal X-Ray Structure Determination of Compound 4a (CCDC 2165468).

Detailed Crystallographic Data.

Diffractometer operator:	C. Jandl
Scanspeed	2-20 s per frame
dx	60 mm
Frames:	4260 measured in 12 data sets
phi-scans with delta phi	0.5
omega-scans with delta omega	0.5
shutterless mode	

Crystal Data:

$[C_{51}H_{55}ClFeN_3P_2Ru]PF_6$	D_x = 1.251 Mg m^{-3}
M_r = 1108.97	Melting point: ? K
Trigonal, *R*3	Mo *K*α radiation, λ = 0.71073 Å
Hall symbol: R 3	Cell parameters from 9459 reflections
a = 26.5456 (13) Å	θ = 2.5–25.7°
c = 21.7085 (17) Å	μ = 0.68 mm^{-1}
V = 13247.9 (19) Å^3	*T* = 100 K
Z = 9	Fragment, red
F(000) = 5111	0.18 × 0.12 × 0.10 mm

Data collection:

Bruker D8 Venture diffractometer	10802 independent reflections
Radiation source: IMS microsource	10673 reflections with $I > 2\sigma(I)$
Helios optic monochromator	R_{int} = 0.047

Detector resolution: 7.5 pixels mm^{-1}	θ_{max} = 25.4°, θ_{min} = 2.0°
phi– and ω–rotation scans	h = −31 31
Absorption correction: multi-scan *SADABS* 2016/2, Bruker	k = −31 31
T_{min} = 0.708, T_{max} = 0.745	l = −26 26
196511 measured reflections	

Data refinement:

Refinement on F^2	Hydrogen site location: inferred from neighbouring sites
Least-squares matrix: full	H-atom parameters constrained
$R[F^2 > 2\sigma(F^2)]$ = 0.025	$W = 1/[\Sigma^2(FO^2) + (0.0238P)^2 + 24.1446P]$ WHERE $P = (FO^2 + 2FC^2)/3$
$wR(F^2)$ = 0.060	$(\Delta/\sigma)_{max}$ = 0.021
S = 1.15	$\Delta\rho_{max}$ = 0.30 e Å^{-3}
10802 reflections	$\Delta\rho_{min}$ = −0.55 e Å^{-3}
812 parameters	Extinction correction: none
1096 restraints	Extinction coefficient: -
0 constraints	Absolute structure: Flack, Parsons[9,10]
Primary atom site location: iterative	Absolute structure parameter: 0.037 (16)
Secondary atom site location: difference Fourier map	

References

1) *APEX suite of crystallographic software*, APEX 3, Version 2016-9.0, Bruker AXS Inc., Madison, Wisconsin, USA, 2016.

2) *SAINT*, Version 8.40A and *SADABS*, Version 2016/2, Bruker AXS Inc., Madison, Wisconsin, USA, 2016/2019.

3) G. M. Sheldrick, *Acta Crystallogr. Sect. A* **2015**, *71*, 3–8.

4) G. M. Sheldrick, *Acta Crystallogr. Sect. C* **2015**, *71*, 3–8.

5) C. B. Hübschle, G. M. Sheldrick, B. Dittrich, *J. Appl. Cryst.* **2011**, *44*, 1281-1284

6) *International Tables for Crystallography, Vol. C* (Ed.: A. J. Wilson), Kluwer Academic Publishers, Dordrecht, The Netherlands, **1992**, Tables 6.1.1.4 (pp. 500–502), 4.2.6.8 (pp. 219–222), and 4.2.4.2 (pp. 193–199).

7) A. L. Spek, *Acta Crystallogr. Sect. C* **2015**, *71*, 9–18.

8) C. F. Macrae, I. J. Bruno, J. A. Chisholm, P. R. Edgington, P. McCabe, E. Pidcock, L. Rodriguez-Monge, R. Taylor, J. van de Streek, P. A. Wood, *J. Appl. Cryst.* **2008**, *41*, 466–470.

9) H. D. Flack, *Acta Crystallogr. Sect A* **1983**, *39*, 876–881.

10) S. Parsons, H. D. Flack, T. Wagner, *Acta Crystallogr. Sect B* **2013**, *69*, 249–259.
